# Standardised *Sonneratia apetala* Buch.-Ham. fruit extract inhibits human neutrophil elastase and attenuates elastase-induced lung injury in mice

**DOI:** 10.3389/fphar.2022.1011216

**Published:** 2022-12-07

**Authors:** Sayantan Sengupta, Nipun Abhinav, Sabita Singh, Joytri Dutta, Ulaganathan Mabalirajan, Karthigeyan Kaliyamurthy, Pulok Kumar Mukherjee, Parasuraman Jaisankar, Arun Bandyopadhyay

**Affiliations:** ^1^ Cardiovascular Disease and Respiratory Disorders Laboratory, Cell Biology and Physiology Division, CSIR-Indian Institute of Chemical Biology, Kolkata, India; ^2^ Department of Natural Products, National Institute of Pharmaceutical Education and Research (NIPER), Kolkata, India; ^3^ Molecular Pathobiology of Respiratory Diseases Laboratory, Cell Biology and Physiology Department, CSIR-Indian Institute of Chemical Biology, Kolkata, India; ^4^ Academy of Scientific and Innovative Research (AcSIR), Ghaziabad, India; ^5^ Central National Herbarium, Botanical Survey of India, A.J.C.B. Indian Botanic Garden, Howrah, India; ^6^ Institute of Bioresources and Sustainable Development, Imphal, India; ^7^ Laboratory of Catalysis and Chemical Biology, Department of Organic and Medicinal Chemistry, CSIR-Indian Institute of Chemical Biology, Kolkata, India

**Keywords:** *Sonneratia apetala* Buch.-Ham., neutrophil elastase, lung injury, COPD, ellagic acid, LC-MS-MS, HPLC, emphysema

## Abstract

Chronic obstructive pulmonary disease (COPD) along with asthma is a major and increasing global health problem. Smoking contributes to about 80%–90% of total COPD cases in the world. COPD leads to the narrowing of small airways and destruction of lung tissue leading to emphysema primarily caused by neutrophil elastase. Neutrophil elastase plays an important role in disease progression in COPD patients and has emerged as an important target for drug discovery. *Sonneratia apetala* Buch.-Ham. is a mangrove plant belonging to family Sonneratiaceae. It is widely found in the Sundarban regions of India. While the fruits of this plant have antibacterial, antifungal, antioxidant and astringent activities, fruit and leaf extracts have been shown to reduce the symptoms of asthma and cough. The aim of this study is to find whether hydro alcoholic fruit extracts of *S. apetala* inhibit neutrophil elastase and thus prevent the progression of neutrophil elastase-driven lung emphysema. The hydroalcoholic extract, ethanol: water (90:10), of the *S. apetala* Buch.-Ham. fresh fruits (SAM) were used for neutrophil elastase enzyme kinetic assay and IC_50_ of the extract was determined. The novel HPLC method has been developed and the extract was standardized with gallic acid and ellagic acid as standards. The extract was further subjected to LC-MS^2^ profiling to identify key phytochemicals. The standardized SAM extract contains 53 μg/mg of gallic acid and 95 μg/mg of ellagic acid, based on the HPLC calibration curve. SAM also reversed the elastase-induced morphological change of human epithelial cells and prevented the release of ICAM-1 *in vitro* and an MTT assay was conducted to assess the viability. Further, 10 mg/kg SAM had reduced alveolar collapse induced by neutrophil elastase in the mice model. Thus, in this study, we reported for the first time that *S. apetala* fruit extract has the potential to inhibit human neutrophil elastase *in vitro* and *in vivo*.

## Graphical Abstract

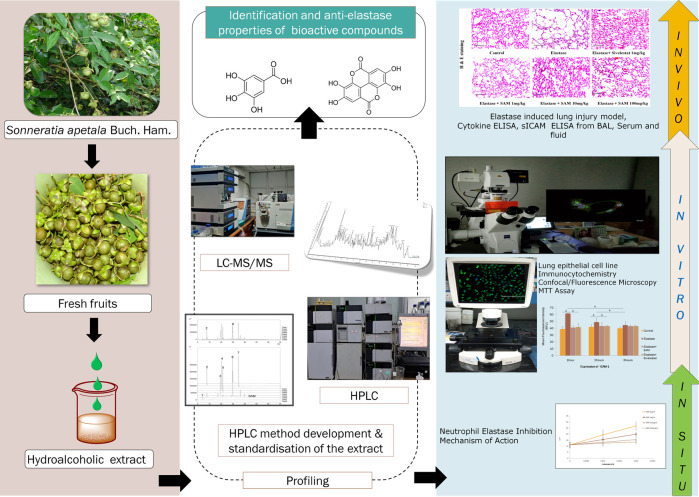

## 1 Introduction

The chronic obstructive pulmonary disease (COPD) is a significant global health issue with rising morbidity and mortality rates. The prevalence of COPD is thought to be around 7% of the population of 30 years and above. Adults over the age of 65 may have a prevalence of up to 10%. (Fukuchi et al., 2004; Menezes et al., 2005; Halbert et al., 2006; Buist et al., 2007; Ford et al., 2013). Due to under diagnosis and under recognition of COPD, the true prevalence appears to be higher than that which has been recorded (Lopez et al., 2006) and at the moment it ranks as the third largest cause of death globally (Quaderi and Hurst, 2018). The long-term exposure to toxic materials or gases is linked to COPD through a persistent inflammatory response in the peripheral airways and lung parenchyma. The majority of COPD instances are caused by harmful tobacco smoke particles, which cause ongoing chronic inflammation. The pulmonary element is characterised through airflow channel that is not always very well reversible and typically progressive (Hogg, 2004). Due to emphysematous lesions, the small airways’ increased resistance and the lung’s increased compliance together led to the airflow difficulties (Hogg et al., 2004). The foremost signs and manifestations of COPD are dyspnoea and persistent production of sputum. Exertional dyspnoea is a classic early COPD symptom. Although different factors are worried in inflicting dyspnoea, narrowing of airways is a crucial element related to it (O’Donnell and Webb, 1993; O’Donnell et al., 2001). Fibrosis of the small airways causes thickening of internal wall resulting in the narrowing of the airways in COPD together with collapse of the small airways during expiration due to loss of radial elasticity and rupture of the pulmonary alveolar tissue (Hogg et al., 1968; McDonough et al., 2011). Goblet cell proliferation and mucus gland hyperplasia are the causes of increased sputum production (Lai and Rogers, 2010). Because COPD cases present with irreversible limitations in proper airflow in lungs and their disease often progresses throughout life, pharmaceutical and non-pharmacological treatments should work to reduce symptoms, enhancing the quality of life, increase exercise tolerance, stop the course of the disease and concomitant conditions, and enhance prognosis (Braido et al., 2015). Pharmacological therapy and non-pharmacological interventions, such as quitting smoking, reducing other risk factors, immunisation, oxygen therapy, and pulmonary rehabilitation, should be used to reach these goals. The disease severity should be appropriately assessed, and there should be ongoing evaluation to see if the patient is having the desired therapeutic response. Spilling of inflammatory mediators from the lungs to circulation is very common in COPD patients which may trigger and worsen diabetes, ischemic normocytic anaemia, heart disease, depression osteoporosis, heart failure and lung cancer (Barnes, 2009). Therefore, it is crucial for COPD patients to have proper comorbidity control (Agustí et al., 2003; Sorianoet al., 2005). As current therapies do not change the progressive burden of the disease, it is necessary to develop efficient therapeutic strategies (Barnes, 2000). By finding new targets and developing target specific small molecule inhibitors or using herbal extracts as a whole or isolating new compounds from those extracts can be beneficial for treating COPD (Barnes, 2013).

Neutrophil elastase plays a significant role in the progression of COPD. Neutrophil elastase targeting may be helpful in the prevention of inflammatory lung disease, according to numerous clinical observations. The lung inflammation in COPD occurs when the lungs react to dangerous particles and gases, leading to an abnormal rise in airway inflammatory cells like neutrophils, T lymphocytes, and macrophages. Neutrophils are directly recruited from the bloodstream and cause destruction to the parenchymal layer by releasing human neutrophil elastase (Donnelly and Rogers, 2003). A cytokine network that stimulates neutrophil recruitment leads to an imbalance in the protease-antiprotease ratio (Abboud and Vimalanathan, 2008). Human Neutrophil Elastase (EC 3.4.21.37), is a serine protease that is largely stored in the azurophilic granules of multinuclear neutrophils and is a member of the chymotrypsin superfamily. Neutrophils produce excess elastase which is secreted extracellularly during the inflammatory stages of COPD. With 218 amino acids and four disulfide bridges holding it together, human neutrophil elastase weighs between 29 and 33 kDa (Sinha et al., 1987). Its catalytic triad, which consists of Ser195, His57, and Asp102 residues, regulates their activity. The trio is widely separated in the primary sequence, but they are united at the active site in the tertiary structure (Bode et al., 1989; Korkmaz et al., 2008). Human Neutrophil Elastase is a multifaceted enzyme that works against pathogens to control inflammation and maintain tissue homeostasis (Pham, 2006). HNE may hydrolyze cleavable amide bonds using its active serine and can break down a variety of structural proteins, including collagen, elastin, laminin proteoglycans, and fibronectin (Chua and Laurent, 2006; Pham, 2008). Through the breakdown of adhesion molecules, HNE plays a significant part in chemotaxis and aids in neutrophil migration (Cepinskas et al., 1999; Hermant et al., 2003). Under physiological circumstances that control inflammatory processes and prevent the negative consequences of extracellular HNE, endogenous inhibitors, such as Alpha-1-antitrypsin (α1AT), 2-macroglobulin, elafin, and secretory leukocyte protease inhibitors (SLPI) lessen tissue damage (Tremblay et al., 2003; Ohmoto et al., 2001; Heutinck et al., 2010). Acute lung injury (ALI) (Kawabata et al., 2002), cystic fibrosis (Gifford and Chalmers, 2014), asthma (Guay et al., 2006), and acute respiratory distress syndrome (ARDS), among others, are all caused by extracellular HNE. HNE has essential functions in a multitude of other conditions, including psoriasis and other skin problems (Meyer-Hoffert et al., 2004; Marto et al., 2018), rheumatoid arthritis (Cawston and Young, 2010), atherosclerosis (Henriksen and Sallenave, 2008), and various cancers affecting lungs and breasts (Hunt et al., 2013; Albrengues et al., 2018). Recent reports suggest the role of major components of neutrophil innate immunity as assessed in patients suffering from COVID-19 (Guéant et al., 2021) and the possible involvement of neutrophil elastase in COVID-19 related lung diseases (Mohamed et al., 2020). So, human neutrophil elastase is viewed as a potentially effective therapeutic target (Henriksen 2014).

Neutrophil elastase inhibitors can be naturally obtained from herbal sources or chemically synthesised in laboratory. Though a large number of synthetic drugs undergo clinical trials, only Sivelestat (ONO-5046) is approved in Japan as human neutrophil elastase inhibitor used to treat patients with ARDS (Iwata et al., 2010) and recently Sivelestat has been proposed to treat acute lung injury in COVID patients (Sahebnasagh et al., 2020). The creation of innovative medications to prevent and treat disorders brought on by the overactive elastase or the pathological breakdown of elastin could be greatly impacted by the identification of new elastase inhibitors from natural sources, including plants, animals, fungus, bacteria, and sponges (Marinaccio et al., 2022). A naturally occurring macrocyclic peptide called Cyclotheonellazole A (CTL-A) has been described as a powerful elastase inhibitor (Cui et al., 2022). An unusual elastase inhibitor, ShSPI, has been discovered from the poisonous gland of the centipede *Scolopendra hananum* (Luan et al., 2019). Human neutrophil elastase can be inhibited by the 57 amino acid long, cysteine-rich polypeptide guamerin, which has a K_i_ value of 8.1 × 10^−14^ M (Jung et al., 1995). Strong anti-elastase activity was found in compounds derived from marine cyanobacteria (Keller et al., 2020).

Due to its affordability and little side effects, the use of plants based natural products in the treatment of diseases has gained increased attention in recent years (Lahouar et al., 2015; Uchida et al., 2017). The mangrove plant *S. apetala*, commonly known as mangrove apple (Keora in West Bengal and Bangladesh), belonging to Sonneratiaceae family and is found in coastal areas of India, Bangladesh, Malaysia, China, New Guinea, Myanmar and other countries (Patra et al., 2015; Hossain et al., 2016a; Nasrin et al., 2017). *S. apetala* is an exotic mangrove species that was brought to China from Bengal (India) and Sri Lanka (Ren et al., 2009). It is the predominant species of mangroves in the southeast coastal parts of the country due to its excellent adaptability, fast growth, enhanced crop setting rate, and other characteristics (Jia et al., 2014). Keora fruit is edible and is typically prepared for food by people residing in coastal regions of its origin. It is also processed and marketed as fermented juice, soft drinks, and other products (Mollik et al., 2010; Shefa et al., 2014). Additionally, due to its pharmacological value it is frequently used as medication by locals to heal ailments including hepatitis, diarrhoea, bruises, *etc.* Additionally, Bangladeshi traditional healers prescribe *S. apetala* as anti-inflammatory remedy to reduce gastrointestinal complications like diarrhoea, dysentery, and stomach cramps (Bandaranayke 2002; Shefa et al., 2014; Teja and Ravishankar, 2013). In addition to leaf and flower, *S. apetala*’s fruit is used as medication for internal ailments in China to treat coughs and sprains (Ji et al., 2005). It has been demonstrated that the fruits and bark of *S. apetala* are effective in treating fevers, bleeding, wheezing, swellings, ulcers, and sprains (Bandaranayake, 1998). Fruit extracts from *S. apetala* have been shown to possess a variety of biological activities, including antioxidant, anti-diabetic, anti-cancer, and antibacterial effects (Jaimini et al., 2011; Patra et al., 2015). The majority of the bioactive components in this mangrove plant are polyphenols (Sonneradon A, Gallic acid, Ellagic acid, Caffeic acid, Betulinic acid *etc.*), tannins (Catechin, Epicatechin), flavonoids (Apigenin, Luteoline), and carbohydrates, which contribute to the plant’s antioxidant activity and make it a possible source of natural antioxidants (Lin et al., 2009; Mun et al., 2010; Cao et al., 2015; Hossain et al., 2016a; Yi et al., 2017). Additionally found in *S. apetala* are steroids, lactones carboxylic acids, and triterpenes, (Bandaranayake, 2002; Cao et al., 2015).

It has been reported previously that plant flavonoids have anti-elastase activities (Jakimiuk et al., 2021). Neutrophil elastase also plays a direct role in mucus hyper production and mucin over secretion in lung disorders by directly activating the MUC5AC gene (Voynow et al., 1999). As fruits of *S. apetala* is rich in flavonoids and consumed traditionally to treat wheezing, cough and asthma we hypothesized that it has the potential to inhibit human neutrophil elastase. In this study, we demonstrated for the first time the effect of fruit extract of *S. apetala* on human neutrophil elastase and its implications in mouse lung injury.

## 2 Materials and methods

### 2.1 Reagents and instruments

HPLC grade Methanol was purchased from Finar Ltd. India; LC/MS grade Acetonitrile by Fisher Chemicals. Ascorbic acid, Gallic acid, vanillic acid, Syringic acid Ellagic acid was supplied by (TCI India) chemical Pvt. Ltd. and Quercetin by MP Biomedicals LLP, Germany. ULC/MS grade formic acid was supplied by Biosolve B.V., Netherlands. Ethanol was supplied by Merck. The HPLC analysis was carried out using Shimadzu Prominence UFLC system (Shimadzu, Kyoto, Japan) equipped with LC-20AD and LC-20AT prominence liquid chromatography pumps, DGU-20A3 prominence degasser, CBM-20A prominence communications bus module, SPD-20A prominence UV/VIS detector. LC-MS/MS experiments were performed in LTQ XL™ Linear Ion Trap Mass Spectrometer.

### 2.2 Plant material

The plant was collected and identified by Dr. K. Karthigeyan (Scientist-E at Central National Herbarium, Botanical Survey of India, A.J.C.B. Indian Botanic Garden, and Howrah, India). The plant was collected at Hero—1, Jharkhali area of Sundarbans, West Bengal, India (GPS coordinates: 22 01. 448, 88 39. 88). The field specimen number is 65153 assigned by Central National Herbarium (CAL).

### 2.3 Extraction

500 gm of fresh fruit was accurately weighed; the whole fruit was washed and crushed. The crushed material was extracted with hydroalcoholic mixture (Ethanol: water 90:10, 5 L) for 24 h (3 times) at room temperature. The extract was further collected and filtered through Type- 1 qualitative Whatman filter paper and filtrate was dried in rotary evaporator. The extract was further lyophilized to obtain 18 gm of brown amorphous powder. The extracted sample was stored at (−20°C) for further use (Hossain et al., 2016b; Liu et al., 2019; Yi et al., 2020).

### 2.4 HPLC analysis and method development for simultaneous detection of gallic acid and ellagic acid in *S.apetala* fruit extract

**FIGURE 1:**
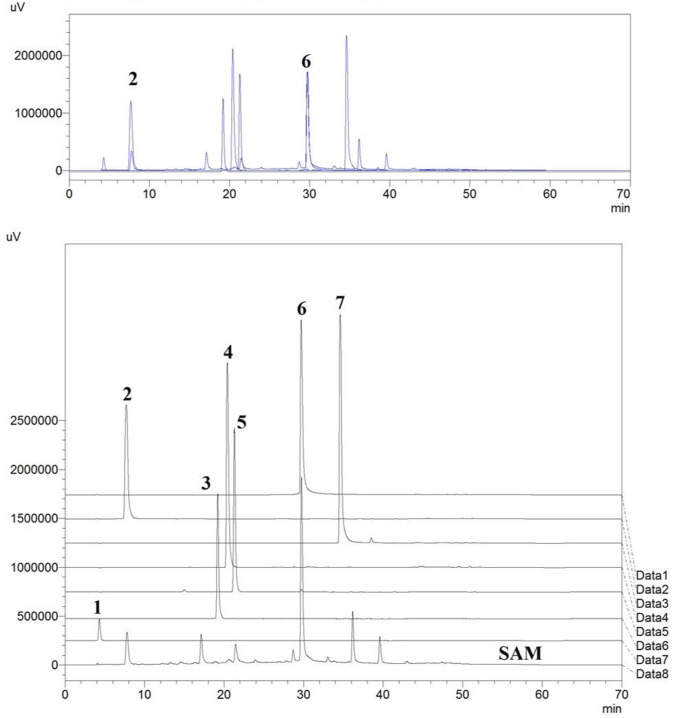
Standards vis. (1) Ascorbic acid, (2) Gallic acid, (3) Vanillic acid, (4) Caffeic acid, (5) Syringic acid, (6) Ellagic acid, (7) Quercetin overlapped with the HPLC chromatogram of extract of fruits of *S. apetala*.

All seven standard compounds which are reported to be present in fruit, vis. 1) Ascorbic acid (AA), 2) Gallic acid (GA), 3) Vanillic Acid (VA), 4) Caffeic acid (CA), 5) Syringic acid (SA), 6) Ellagic acid (EA) and 7) Quercetin (QR), were accurately weighed and transferred to volumetric flasks and dissolved in HPLC grade Methanol to achieve 1 mg/ml concentration (Hossain et al., 2016a; Islam, 2019; Liu et al., 2019; Yi et al., 2020). The overlap of HPLC chromatogram of standard compounds (1-7) and SAM (5 mg/ml) showed that the standard compound 2) Gallic acid and 6) Ellagic acid were present in this extract (Hossain et al., 2016b), which was further used for the method development and standardization purpose (Figure 1). The extract (SAM) was dissolved in HPLC grade MeOH to achieve a 5 mg/ml concentration and filtered through a 0.22 µM filter. This same stock concentration was used throughout the study. The standard 2) and 6) were then serially diluted to achieve the six concentrations vis 50 μg/ml, 100 μg/ml, 250 μg/ml, 500 μg/ml, 750 μg/ml, 1,000 μg/ml which were used for calibration and method development. Injection volume for standards and SAM was identical throughout the HPLC study i.e., 20 µl. The aliquots of 20 µl were injected using SIL-20AC HT prominence auto sampler. The separation was achieved on a Phenomenex reverse phase HPLC column (Luna^®^ RP C_18_ column 4.6 mm × 260 mm), 5 µ particle size, and elution was carried out using a mobile phase consisting of Methanol (A) and Water (0.1% Formic Acid) (B) using a gradient system with a flow rate of 0.8 ml/min as described in (Table 1). The eluate was monitored at 260 nm. Data analysis was performed in LC solution post-run analysis software (version 1.25) (Shimadzu, Kyoto, Japan).

**TABLE 1:** *S. apetala* fruit extract HPLC gradient program.

Time(min.)	A (methanol)	B (Water+ 0.1% FA) %
0	20	80
30.00	65	35
35.00	70	30
40.00	90	10
50.00	90	10
55.00	20	80
70.00	20	80

### 2.5 LC-MS/MS spectroscopy

The SAM sample was prepared by dissolving 5 mg extract in LC-MS grade MeOH and filtered through a 0.22 µM filter. The Injection volume was 5 µl. The LC-MS was equipped with Hhypersil Gold C_18_ column (diameter 100 mm × 2.1 mm, particle size1.9 μM). LC analysis used gradient of solvent A (acetonitrile +0.1% formic acid) and solvent B (HPLC grade H_2_O+ 0.1% formic acid). The analyzer was set to positive mode, capillary temperature set to 320°C, source voltage was 5.00 kV, Capillary voltage 45.00 V, Tube Lens Voltage (V) = 110.04. MS Run Time (min): 32.00. Data analysis was performed in Thermo Scientific Xcalibur™ Software.

### 2.6 Measurement of elastase activity and IC_50_ determination

200 μg/ml human neutrophil elastase (Calbiochem, United States) and 1.2 mM N-(OMe-succinyl)-Ala-Ala-Pro-Val-p-nitroanilide (Sigma-Aldrich), all of which are prepared using 0.1 M Tris-NaCl buffer, were used to test the activity of inhibiting the human neutrophil elastase (pH 7.5). A 96-well microtiter plate was pre-incubated at room temperature for 15 min with SAM (1 μl), 50 μl elastase (as prepared above), and 24 μl Tris-NaCl (pH 7.5). 25 μl of substrate solutions were added to each well at the end of incubation. Calculating the absorbance at 405 nm every 30 s for a total of 2 hours allowed researchers to track the reaction. For gallic acid and ellagic acid a stock concentration of 1 mg/ml was prepared and similar experiments were performed. Using GraphPad Prism version 8.4, percentage inhibition was obtained after removing the blank from each and compared to the standard (without the extract).

To ascertain the mechanism of inhibition, we incubated 4 μg/ml HNE with three distinct concentrations (100 mM, 500 mM, and 1,000 mM) of the substrate N-(OMe-succinyl)-Ala-Ala-Pro-Val-p-nitroanilide and concentrations 0 μg/ml, 1 μg/ml, 100 μg/ml and 1,000 μg/ml of the fruit extracts (100 µl total volume). The identical procedure as previously described was used to perform the reaction using the Spectramax multimode plate reader. Utilizing GraphPad Prism version 8.4, the reaction velocities were calculated after blank correction. The subsequent double reciprocal plots and substrate titration plots were created (Budnjo et al., 2014; Milicaj et al., 2022).

### 2.7 General cell culture procedures

A549 cells were procured from NCCS, Pune, and cultured at 37°C in a humid atmosphere using F12K medium (Gibco) containing 10% fetal bovine serum (Life Technologies).

### 2.8 Cell viability assay

In a 96-well plate, 5,000 A549 cells were placed each well. After 24 h of seeding, the cells were exposed to 100 μM elastase or vehicle. After that, these were also given SAM, sivelestat (Sigma-Aldrich), or DMSO. The cells were then cultured for a further 12 h before the MTT reagent (Sigma-Aldrich) at 0.5 μg/ml concentration per well was added. After 4 h of MTT incubation, DMSO was added as a sholubilising agent, and the absorbance at 550 nm was measured.

### 2.9 Cell detachment assay

A549 cells were sown on 6 cm cell culture dishes, and they received the treatments vehicle, elastase, elastase + SAM, and elastase + Sivelestat. After 12 h, the medium was gathered, and centrifugation was used to pellet the separated cells. Trypsin (Thermo) was used to collect attached cells, and the cells were subsequently pelleted. Trypan blue (0.04%) was added after the cell pellet had been redissolved in a new medium. A 10 µl aliquot of the sample was used to count the cells using a haemocytometer. Based on the number of detached cells and the total number of cells present at the start of the experiment, the percent detachment was computed. Prism software was used to create the graphs.

### 2.10 Immunocytochemistry

Cell staining was performed using a well established protocol with slight modifications as reported. The cells were seeded and cultured in 6-well dishes on cover slips, serum starved for 12 h and treated with neutrophil elastase and SAM/Sivelestat. After definite time point culture media was collected and the cells were fixed in 4% paraformaldehyde for 20 min, and permeabilized with 0.1% Triton X-100 for 10 min, followed by blocking with 3% BSA for an hour at room temperature. The cells were incubated with primary antibodies against ICAM-1(Santa Cruz; Cat No. 7891) overnight. Cells were washed in 1x Phosphate Buffer Saline (PBS) three times and stained with Alexa Fluor TM Plus 647 conjugated secondary antibody (Invitrogen; Ref A32795) for 90 min. Cells were washed 3 times after each step with 1 × PBS. Stained cells were mounted with ProLongTM Glass Antifade Mountant with NucBlue TM (Invitrogen, CA), and images were captured within 24 h using Confocal Microscopy at 60× magnification (ZEISS LSM 980) (Mukherjee et al., 2020). Image J software was used to process the image and calculation of Mean fluorescence intensity (MFI).

For live cell experiments the A549 cells were seeded on 6-cm dishes. The cells were treated with vehicle, elastase, elastase + SAM, and elastase + Sivelestat. Cells were treated with two drops of DAPI (Thermo Fischer) after 1, 2 and 3 hours, and washed with PBS after 5 min. Cells were also stained with WGA (Thermo Fischer) in accordance with the instruction booklet, and cell morphology was examined under a Fluorescence microscope (EVOS FL, Thermo Fischer) to check for the morphological changes after each hour.

### 2.11 Measurement of soluble ICAM protein

A549 cells were plated on 6 cm dishes. Three different experimental setups were prepared and the cells were treated with vehicle, elastase, elastase + SAM, and elastase + Sivelestat. After an interval of 1, 2 and 3 h media was collected from the dishes. Media was centrifuged to pellet any debris and cells. The supernatant was collected and used to measure the concentration of soluble ICAM in cells using sICAM ELISA Kit (Invitrogen, Cat. No. EHICAM1) according to manufactures’ instructions (Liu et al., 2018). Similar experiments were performed with mouse serum and BAL fluid as per manufacturer’s instructions.

### 2.12 Mice grouping

The male C57/BL6 mice, which were about 8–10 weeks old, were obtained from the in-house breeding facility at CSIR-Indian Institute of Chemical Biology (IICB), Kolkata and were acclimatized for 1 week at the animal house facility, IICB. All the animal experiments were formally approved by the Institutional animal ethics committee at IICB (Reference Number IICB/AEC/Meeting/July/2021/6) and all the animal experiments were performed following the guidelines of CPCSEA (Committee for the Purpose of Control and Supervision of Experiments on Animals guidelines). Mice were randomly divided into five groups: 1) VEH/VEH (intratracheal vehicle, Tris buffer pH 7.4, administered and vehicle, 50% DMSO diluted with saline, treated mice), 2) NE/VEH (intratracheal neutrophil elastase administered and vehicle-treated mice) and NE/*Sonneratia apetala* extracts—1 mg/kg (c), 10 mg/kg/day 4) and 100 mg/kg/day 5) Intratracheal neutrophil elastase administered and extract treated mice.

### 2.13 Induction of emphysema in mice with human neutrophil elastase

Human neutrophil elastase (Calbiochem, cat no. 324681) was reconstituted in tris buffer pH 7.4. For the induction of elastase-induced emphysema, mice were first anesthetized briefly with isoflurane fumes. Anesthetized mice were placed on wood support at an angle of 45° and carefully grasped the tongue with an upward and leftward position using blunt end forceps. These mice were then instilled with 10 µg human neutrophil elastase (to both NE/VEH and NE/SAM groups) or vehicle (VEH/VEH group) on day 1 through theorotracheal routeusing a pipette as shown in Supplementary Figure S39. Mice were maintained in the same position for 15 s and then placed onward pad for recovery. SAM or vehicle was administered from day 1 to day 6, twice a day. Mice were euthanized on day 6 and blood, BAL fluid and lungs were collected.

Each animal’s H & E-stained lung slices were photographed using a Magnus MLX-i microscope with a ×10 objective lens. The photos were obtained at equal intervals, meandering systematically over the surface of the lung sections. A quadratic test system with 64 points and 8 horizontal lines and 8 vertical lines totaling µm (*d* = µm) was used to statistically examine the photos, using the software STEPanizer (Tschanz et al., 2011). Based on P_ref_ (all points hitting the parenchyma), P_sep_ (points hitting the alveolar septa), and I (intersections of the test line system with the alveolar surface), the mean linear intercept of the airspaces (Lm) of each lung slide was computed as follows: Lm = 2*d* (Σ P_ref_ − Σ P_sep_/Σ I) (Salaets et al., 2020).

As the alveolar collapse is strongly associated with an increase in pro-inflammatory mediators relevant to neutrophil recruitment and activity, we wanted to measure key inflammatory indicators. So mouse lungs were homogenized in a modified RIPA lysis buffer, centrifuged, and the supernatants were stored at −80°C until subsequent analyses. The levels of IL-17, and chemokine C-X-C motif ligand 1 (CXCL1)/KC were quantified using ELISA.

The kits were purchased from R&D System (Minneapolis, MN). The lung tissue homogenates in duplicate were used and ELISA was performed following the manufacturers’ protocol. Results were expressed in picograms and normalized by protein concentrations.

### 2.14 Statistical analysis

Utilizing the GraphPad Prism (version 8.4) and OriginPro 8.5.0, the statistical analysis and graph plotting was performed. The statistically significant differences were determined using an unpaired, two-tailed Student’s t-test. Using one-way ANOVA and the Dunnet *t-*test, comparisons between more than two groups were made. *p < 0.05, **p < 0.01 and ***p < 0.001 are all deemed statistically significant.

## 3 Results

### 3.1 HPLC analysis and method development for simultaneous detection of gallic acid and ellagic acid in *S. apetala* fruit extract

Total 5 parameters were studied to establish the HPLC method including 1) Linearity and Sensitivity 2) Accuracy 3) Intra-day precision 4) Inter-day precisions 5) Robustness. Following are the results obtained for each parameter.

#### 3.1.1 Linearity and sensitivity

HPLC chromatograms were obtained using standards GA and EA in concentration ranging from 50 μg/ml, 100 μg/ml, 250 μg/ml, 500 μg/ml, 750 μg/ml, to 1,000 μg/ml. Calibration curve was plotted between peak area (*Y* axis) and concentration (*X* axis). Chromatogram for each concentration was in acquired triplicate (*n* = 3); Table 2 and (Supplementary Figures S1–S12; Supplementary Tables S1,S2) in supplementary information.

**TABLE 2:** Linearity and sensitivity of gallic acid and ellagic acid.

	R^2^ value	Linear equation	LOD^a^	LOQ^a^
Gallic acid	0.995	Y = 29947.75X + 534827.20	50 μg/ml	50 μg/ml
Ellagic acid	0.955	Y = 85746.32X + 830136.36	50 μg/ml	50 μg/ml

^a^ Based on the signal-to-noise ratio.

#### 3.1.2 Accuracy

Accuracy was estimated by spiking the stock SAM extract (5 mg/ml) with standard gallic acid (GA) with the equivalent of 125 μg/ml, 500 μg/ml and 1,000 μg/ml additional concentrations and 125 μg/ml, 250 μg/ml and 500 μg/ml additional concentrations of Ellagic acid (EA). The recovered amount was calculated as % recovery= (Spiked- Non-Spiked/Spiked) *100. Chromatogram for each concentration was acquired in triplicate (*n* = 3). (Table 3; Supplementary Figures S13–S17; Supplementary Table S3).

**TABLE 3:** Accuracy of gallic acid and ellagic acid.

	Spike added	% Recovery (spike found)	%RSD
Gallic acid	125 μg/ml	98.66	0.017
	500 μg/ml	91.6	0.021
	1,000 μg/ml	92.8	0.041
Ellagic acid	125 μg/ml	93.0	0.021
	250 μg/ml	107.06	0.09
	500 μg/ml	102.8	0.015

#### 3.1.3 Intra-day precision

Intraday precision was obtained by calculating the % RSD of the peak area of gallic acid (500 μg/ml) and ellagic acid (750 μg/ml) in the morning and evening (*n* = 3) for three consecutive days. Intra-day precision was found to be 5%–15% RSD of peak area for gallic acid and 4%–8.8% RSD of peak area of ellagic acid (Supplementary Figures S18–S30; Supplementary Table S4).

#### 3.1.4 Inter-day precision

Intraday precision was obtained by calculating the % RSD of mean peak area of gallic acid (500 μg/ml) and ellagic acid (750 µg/ml)for three consecutive days each day (*n* = 6). Inter-day precision was found to be 13.7% RSD of peak area for gallic acid and 6.91% RSD of peak area of ellagic acid (Supplementary Figures S18–S30; Supplementary Table S4).

#### 3.1.5 Robustness

Robustness of the method was estimated by acquiring chromatogram by deliberately changing method parameters. Changing the UV/V is detector wavelength from original 260 nm to 255 nm and flow rate to 0.80 ml/min to 0.60 ml/min for each gallic acid (500 μg/ml) and ellagic acid (750 µg/ml) (*n* = 3). Wavelength change produced 5.20 % RSD in peak area for Gallic acid and 3.18% RSD of peak area for Ellagic acid. Change in flow rate produced 21.66% RSD of peak area for Gallic acid and 2.5% RSD of peak area for Ellagic acid (Supplementary Figures S31–S36; Supplementary Table S5).

### 3.2 LC-MS/MS profiling of SAM

**FIGURE 2:**
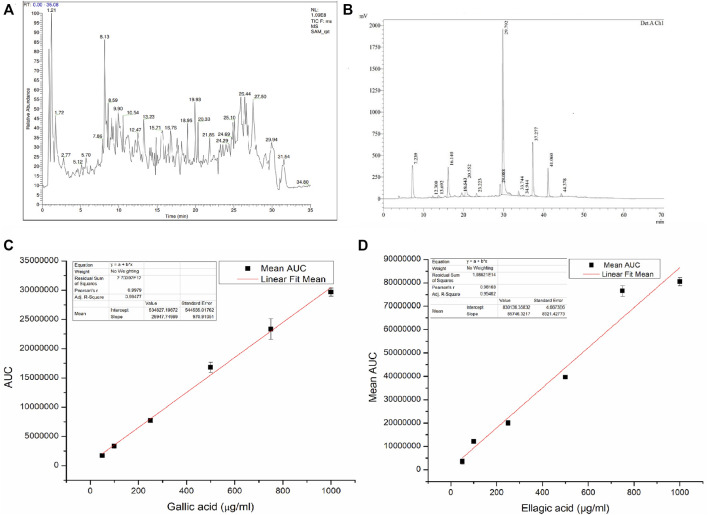
**(A)** LC-MS/MS spectrum of *S. apetala* fruit extract, **(B)** HPLC profile of *S. apetala* extract, **(C)** Linear regression analysis for the HPLC calibration curve for gallic acid, and **(D)** Linear regression analysis for the HPLC calibration curve for ellagic acid.

From the LC-MS/MS spectrum of SAM (Figure 2A), total of 10 compounds were identified using the previous literature, matching the obtained fragments in the LC-MS/MS of SAM. LC-MS/MS confirmed the presence of gallic acid, ellagic acid as well as other polyphenols, fatty esters and terpenoids. Identified compounds are enlisted in (Table 4). It is established by comparing HPLC profile of SAM (5 mg/ml) (Figure 2B) and calibration (Figures 2C,D), that the gallic acid is present 53 µg/mg of extract and ellagic acid are present 95 μg/mg of extract.

**TABLE 4:** Compounds identified from *S. apetala* fruit extract by LC-MS/MS spectroscopy.

S.N.	*m/z* [M+H]^+^	MS/MS fragments	Compound identified
1	295.23	263.16, 255.15, 236.16, 221.09	8,11-Octadecadienoic acid, methyl ester
2	455.22	478.30, 437.19, 427.08, 418.94, 395.25	Stigmasta-5,22-dien-3-ol, acetate
3	375.29	357.27, 343.18, 319.31, 251.17, 119.31	Sonneradon A
4	307.23	289.20, 275.13, 243.02, 151.11	Sonneradon C
5	277.13	259.13, 241.12, 221.07, 207.09, 175.02	Ranuncoside
6	171.03	143.01, 135.01, 125.85, 124.85, 113.84	Gallic acid
7	303.25	275.25, 257.06, 229.09, 215.08, 201.05	Ellagic acid
8	287.05	268.96, 244.99, 179.03	Luteoline
9	457.37	439.30, 411.24, 393.26, 275.16, 249.20, 217.16	Ursolic acid
10	413.38	395.35, 367.31, 297.20, 283.25, 255.16, 241.14, 201.17	Stigmasterol

### 3.3 Elastase activity and IC_50_ determination

**FIGURE 3:**
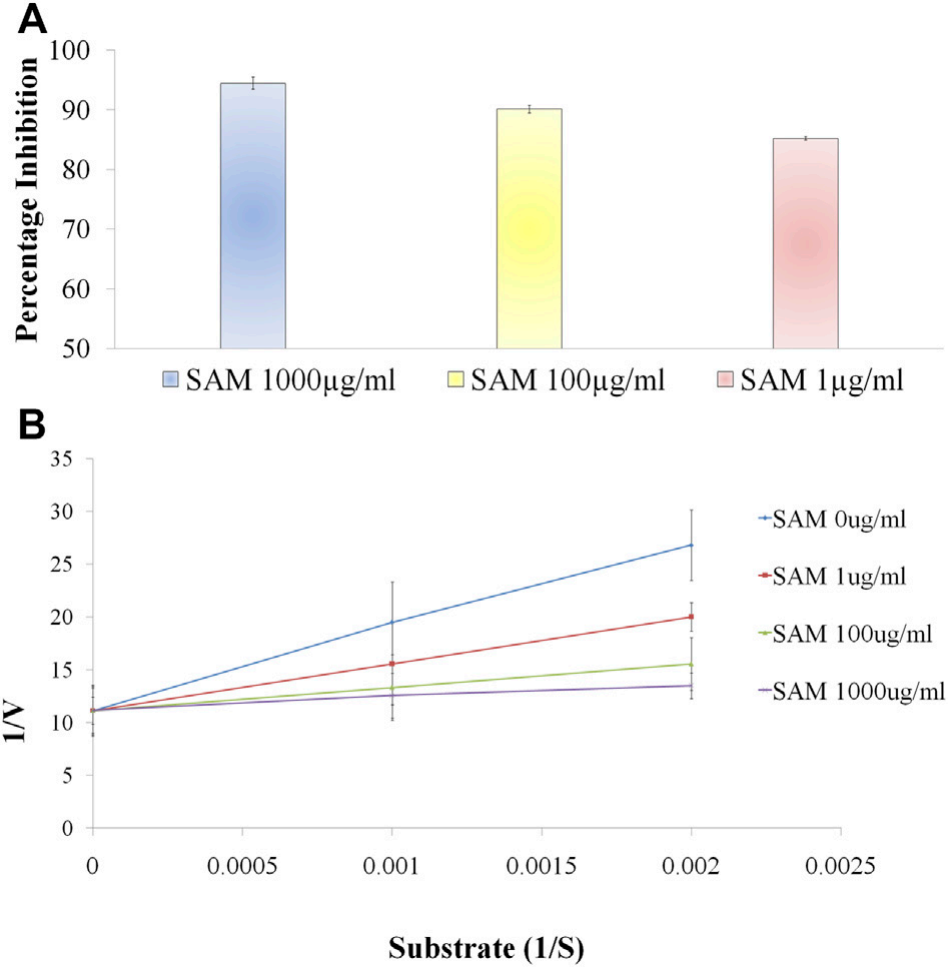
*S. apetala* fruit extract (SAM) inhibits human neutrophil elastase *in situ* **(A)** Enzyme inhibitory activity of various concentrations of SAM against Human Neutrophil Elastase. Enzyme inhibition assay was performed using Human neutrophil elastase incubated with SAM (1 μg/ml, 10 μg/ml and 100 μg/ml) to evaluate the percentage inhibition of the extract. **(B)** Enzyme kinetics to ascertain the mechanism of action of SAM on neutrophil elastase. Substrate titration of steady-state rate of HNE in the presence of SAM Data is presented in double reciprocal format. Data is represented from three independent experiments (*n* = 3).

Dose dependent inhibition was seen when various concentrations of SAM was incubated with human neutrophil elastase enzyme together with known elastase substrate N-(O-Mesuccinyl) Ala-Ala-Pro-Val-p-nitroanilide (Figure 3A)From the inhibitory data IC_50_was calculated and found to be 371.8 ± 3.73 ng/ml as compared to control drug Sivelestat with 65.1 ± 1.13 ng/ml (Table 5).

**TABLE 5:** IC_50_ values of *S. apetala* fruit extract and Sivelestat.

Extract/Compound	IC_50_ (ng/ml)
SAM	371.8 ± 3.73
Sivelestat	65.1 ± 1.13

To comprehend the mode of inhibition of synthetic drugs against elastase inhibition, kinetic experiments were carried out. SAM was used to ascertain the method of inhibition based on the IC_50_. The 1/V Lineweaver Burk plot of the enzyme’s kinetics for the substrate N-(O-Mesuccinyl) Ala-Ala-Pro-Val-p-nitroanilide 1/(S) in the presence of different inhibitor doses showed a sequence of straight lines. SAM’s Lineweaver Burk plot demonstrated that V_max_ stayed constant without drastically changing the gradient. Even though V_max_ is somewhat different, K_m_ stays the same as concentration increases. This behavior suggests that the SAM is a competitive inhibitor of the enzyme (Figure 3B) (Sengupta et al., 2022).

### 3.4 *In vitro* studies

**FIGURE 4:**
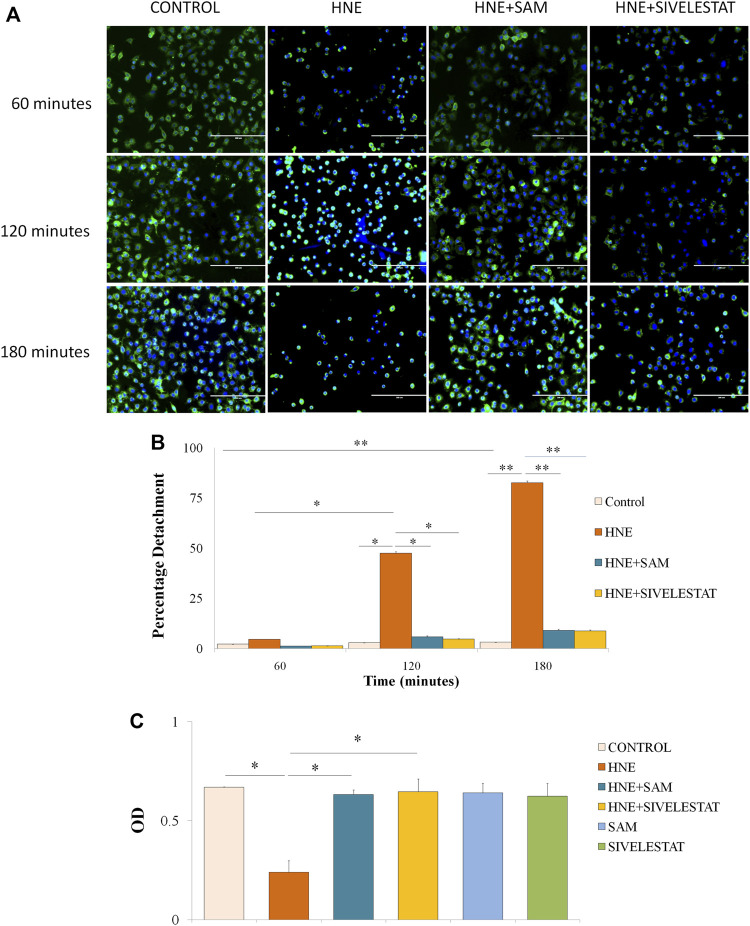
*S. apetala* fruit extract exerts inhibitory effect on elastase-induced human lung epithelial cells **(A)** A549 cells were treated with elastase. After 2 hours of incubation, elastase triggered the rounding of A549 cells. The combination of SAM or sivelestat therapy reduced the impact of elastase, as shown under a fluorescence microscope at a 10× magnification with WGA (green) staining the cell membrane and DAPI (blue) staining the nucleus. **(B)** Percentage cell detachment from culture plate after treatment with elastase after every 1 hour. **(C)** MTT Assay was done to ascertain the cell viability caused by elastase and/or SAM. Elastase and SAM/Sivelestat treated cells were incubated with MTT reagent followed by solubilising agent and subsequent OD was measured. All results are obtained from three different sets of experiments (*n* = 3) (*p < 0.05, **<0.01).

As compared to controls, A549 cells began to congregate after 1 h of neutrophil elastase treatment and began to separate from the cell culture dish after 2 h. The above incident was fully avoided by co-treatment with SAM or sivelestat. In contrast, the cells are entirely detached from the plate after 3 h of treatment, with the exception of a few, indicating a possibility of cell death or apoptosis. Treatment with SAM or sivelestat for 2 h as opposed to 3 h in certain cases prevented the cells from desquamating, although there was a suggestion of apoptosis, indicating that SAM is reversible and a free enzyme is the cause of the activity (Figure 4A). The most foretelling and rapid occurrence that occurred following elastase therapy was the morphological transformation of A549 cells from the epithelium to a slightly oval/rounded, constricted appearance (Figure 4A). Elastase’s action is visible within two to 3 h, which suggests that its early onset is independent to cell death. Comparisons were made with the elastase inhibitor sivelestat as a reference. After each hour cell detachment due to elastase and its prevention by SAM and sivelestat was calculated. Significant cell detachment was observed after 120 min with nearly 48% cells were found floating in the media. At the end of 3 h the detachment was significantly increased to 82% which was successfully reversed by co-treatment of fruit extract (Figure 4B). The difference in percentage detachment with every passing hour is also significant. The MTT assay was used to determine the viability of the cells, and co-treatment with SAM and sivelestat restored the cells’ viability after 12 h of elastase treatment. Interestingly, sivelestat or SAM had no discernible impact on cells alone (Figure 4C).

**FIGURE 5:**
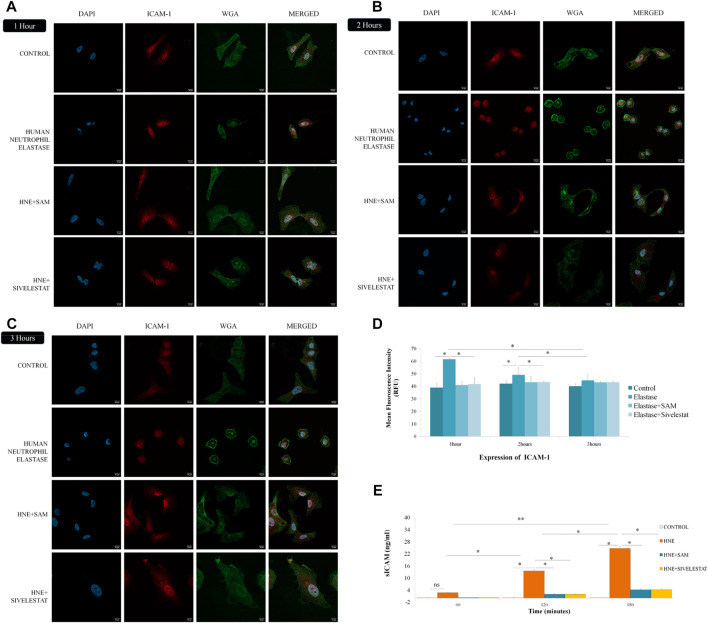
*S. apetala* fruit extracts ameliorates ICAM-1 release induced by neutrophil elastase *in vitro* **(A)** The ICAM-1 expression in lung epithelial cells was significantly increased after 1 h of elastase treatment. **(B)** Increase in ICAM-1 expression after 2 h with deformation in cell shape was restored in SAM/Sivelestat groups. **(C)** Complete rounding of elongated epithelial cells with increased ICAM-1 expression reversed by concomitant treatment of SAM/Sivelestat **(C)** as assessed under Confocal microscope at 60× magnification stained with DAPI (blue). anti-ICAM-1 antibody (red), and WGA (green). **(D)** Mean fluorescence intensity (MFI) was calculated for ICAM-1 expression at each time point. **(E)** sICAM levels were measured after 1, 2 and 3 h of elastase treatment by ELISA. Significant increase in sICAM levels were observed after second and third hour which was reversed by treatment with SAM/Sivelestat.

As observed under Confocal microscope the control cells showed healthy columnar shape which when treated with neutrophil elastase changed moderately into a rounded appearance. The expression of ICAM-1 (stained in red), remained unaltered after 1 h of neutrophil elastase treatment (Figure 5A). Thereafter, the protein expression gradually decreased with every passing hour. The levels of ICAM-1 and shape of cells (cell membrane stained with green WGA) were restored as compared to control in cells treated with SAM/Sivelestat along with elastase (Figures 5B,C). The mean fluroscence intensity as calculated is shown in Figure 5D. As the ICAM-1 within the cells decreased, it was perceptible that it was released in cell culture supernatants. sICAM is secreted in the cell culture media as a result of elastase treatment, which results in the cell detachment. As observed before the levels of sICAM were significantly increased to 13.49 ng/ml at the end of second hour. By the end of the experiment the levels rose to 24.64 ng/ml with elastase treatment and thus proportionally increased with passage of time (Figure 5B). These results validated that ICAM-1 is induced by elastase which is released in the media causing cells to desquamate.

**FIGURE 6:**
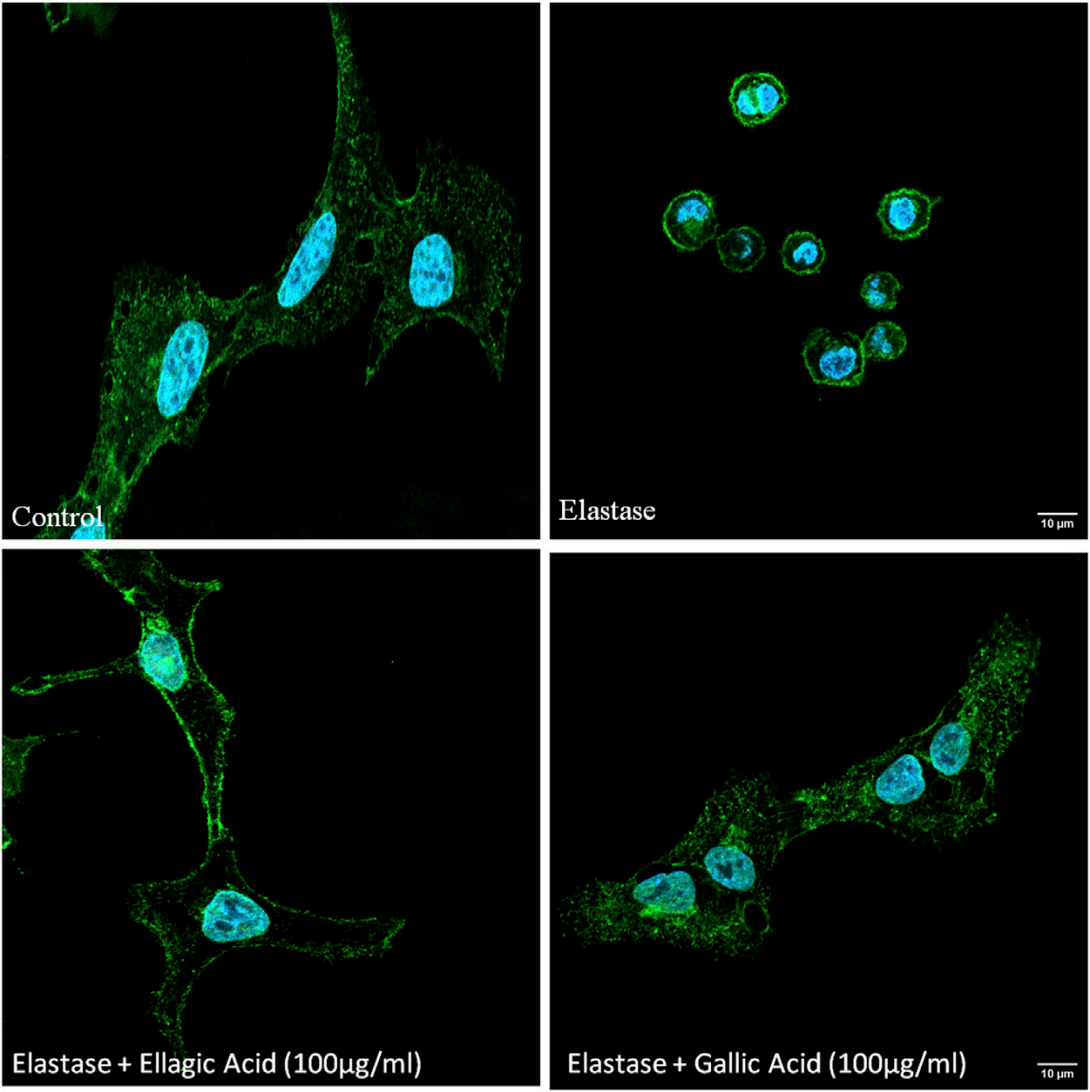
Ellagic acid and gallic acid have anti-elastase effects and protect against elastase induced lung morphological change. Inhibitory effects of gallic acid and ellagic acid on human neutrophil elastase induced lung epithelial cells. Cell membrane was stained with WGA (green) and nucleuii were counterstained with DAPI (blue) and assessed under Confocal microscope (×60).

The effect of Ellagic acid and gallic acid on human neutrophil elastase was determined using a enzyme substrate assay similar to the whole fruit extract. IC_50_ values of ellagic acid and gallic acid was found to be 962 ± 2.43 ng/ml and 841 ± 5.93 ng/ml (Table 6) respectively. Both the bioactive compounds also prevented elastase induced structural abnormalities in lung epithelial cells as assessed after 3 h of treatment (Figure 6).

**TABLE 6:** IC_50_ values of ellagic acid and gallic acid.

Compound	IC_50_ (ng/ml)
Ellagic acid	841 ± 5,93
Gallic acid	962 ± 2.43

### 3.5 *In vivo* assay

**FIGURE 7:**
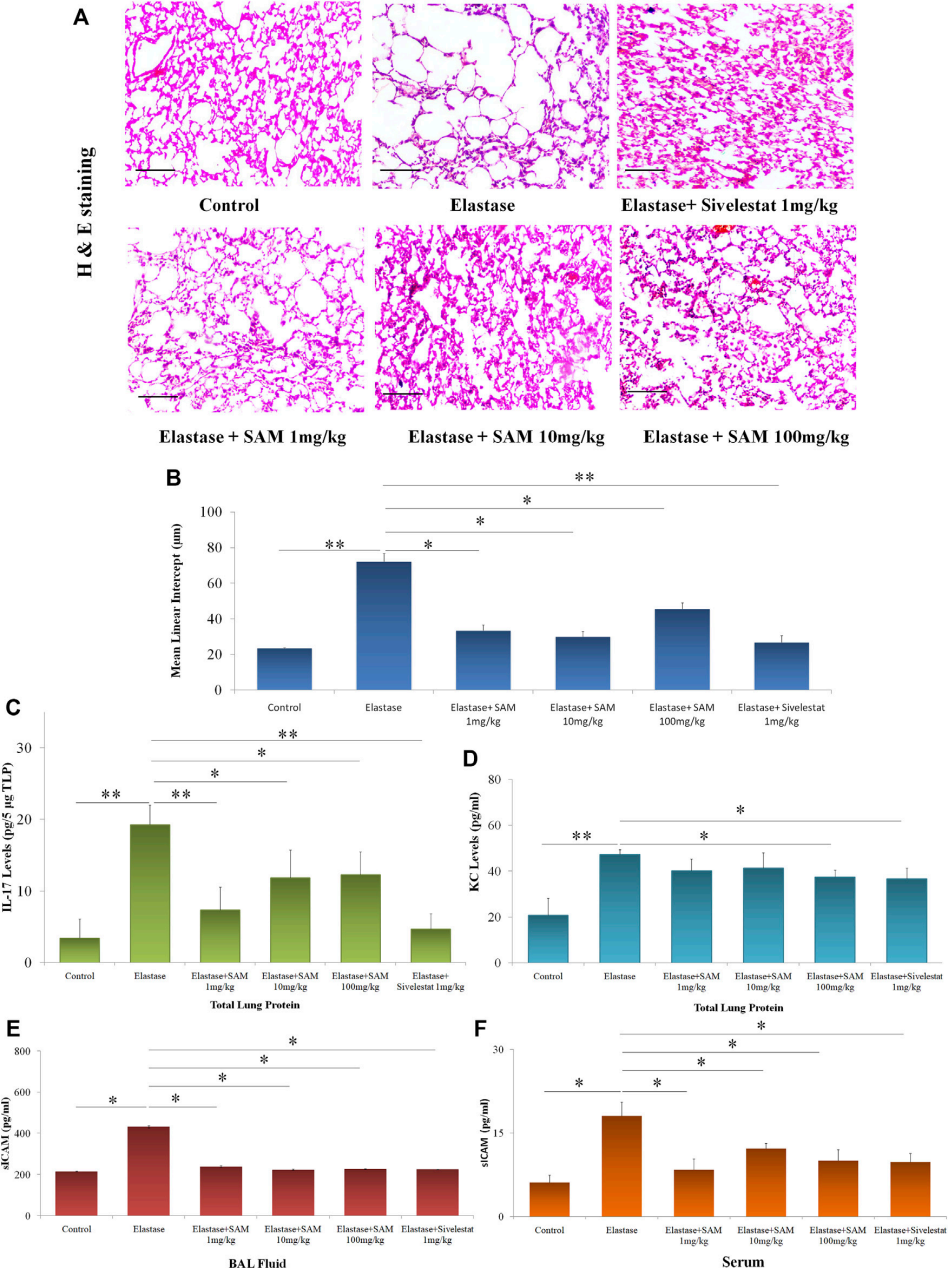
*S. apetala* fruit extract reverses emphysematous changes *in vivo* **(A)** HnE Staining of mouse lung section before and after treatment as compared to control mouse. **(B)** Mean linear intercept (MLI) of mice lungs as assessed from sectioning. **(C)** IL-17A and **(D)** KC levels were measured in lung tissue homogenate of mice by ELISA. *n* = three to five per group; *<0.05 compared to Control, **<0.01 compared to NE. **(E)** BAL sICAM levels and **(F)** serum sICAM levels of elastase and elastase + SAM/Sivelestat treated mice were measured by ELISA as compared to control. All experiments were conducted thrice (*p < 0.05).

We decided to examine the effects of SAM in a mouse model of elastase-induced emphysema since we discovered that it can effectively inhibit neutrophil elastase and diminishes elastase-induced cell detachment. We used the well-known elastase-induced emphysema model, depicted in Supplementary Figure S38, as previously described, to ascertain the same. Elastase + VEH group experienced alveolar collapse compared to control mice, as illustrated in Figure 7A. However, therapy with SAM/Sivelestat with varying concentrations decreased this collapse. The mean linear intercept assay provided proof of this. Figure 7B demonstrates that elastase + VEH mice exhibited significantly higher Mean Linear Intercepts than control mice and mice treated with co-treatment of elastase and SAM/Sivelestat. To investigate the levels of inflammatory markers and sICAM levels associated with COPD, we performed ELISA in the lung tissue homogenates and serum and BAL fluid respectively. Significantly increased IL-17 protein expression was detected in homogenates in NE treated mice compared to the control mice which received saline. However, there was a marked reduction in the IL-17 levels in the NE mice group administered with SAM 1 mg/kg. The IL-17 levels were also reduced in the other NE mice groups treated with SAM concentrations of 10 mg/kg and 100 mg/kg (Figure 7C). In addition, we measured KC levels, the mouse ortholog of IL-8, in lung tissue. The KC levels were notably upregulated in mice administered with NE in comparison to the control mice. A decrease in KC levels was detected in the NE + SAM groups compared to the NE mice group (Figure 7D). Significant increase in sICAM levels was also observed in serum and BAL of mice with elastase treatment which is subsequently reduced with concomitant SAM treatment (Figures 7E,F).

## 4 Discussion

Smoking, exposure to air pollution, and the combustion of biomass fuels are the key contributors to COPD, the third biggest cause of mortality in the world today. Similar to Cystic Fibrosis, neutrophilic inflammation is a prominent feature of COPD. An imbalance in the protease-antiprotease ratio brought on by tobacco smoke and other irritants promotes neutrophil recruitment, which in turn sets off a remorseless cycle of airway alteration and inflammation (Barnes and Celli, 2009). Acute bronchitis exacerbations brought on by bacterial or viral infections, which are associated with greater NE levels, are the main cause of afflictions and deaths in COPD (Beasley et al., 2012; Thulborn et al., 2019). It has been demonstrated that peripheral airway dysfunction and sputum neutrophil counts are correlated with the deterioration of lung function (Stănescu et al., 1996; O'Donnellet al., 2004). Patients with various respiratory disorders have remarkably elevated levels of neutrophil elastase in their bronchoalveolar lavage, sputum, and fluid (Oriano et al., 2020; Keir and Chalmers, 2022; Kummarapurugu et al., 2022; Margaroli et al., 2022); as a result, this enzyme contributes to tissue destruction, which is primarily linked to respiratory disorders (Chua and Laurent, 2006; Pham 2008). The pathogenic effects of neutrophil elastase include hyperresponsiveness in small airways (Suzuki et al., 1996), metaplasia concerning secretary cells (Lucey et al., 1990) and secretion of mucus (Schuster et al., 1992). Leukocyte adhesion modulation (Cai and Wright 1996), alteration in interleukin-8 gene expression (Nakamura et al., 1992), and proliferation of smooth muscle cell (Thompson and Rabinovitch, 1996) are just a few indications. Together, in the COPD airway, the protease-antiprotease activity is largely regulated by NE and other proteases. For instance, there is strong evidence of the development of emphysema after smoking exposure which depends on MMP-12/Macrophage elastase (Hautamaki et al., 1997). Emphysema is brought on by NE through activating MMPs and cysteine cathepsins, while NE maintains MMP activity by degrading TIMP-1, a significant MMP inhibitor (Murphy 2011). Uncontrolled NE activity results from the oxidation and inactivation of α-1-antitrypsin by reactive oxygen radicals (Taggart et al., 2000). The activation of neutrophil extracellular trap (NET) release into the airway environment by NE and MPO spreads the proteolytic and pro-inflammatory actions of NE and neutrophil granules (Genschmer et al., 2019). Reduced lung capacity, more exacerbations, and reduced neutrophil phagocytosis are all linked to neutrophil extracellular trap (NET) abundance in the airway (Dickeret al., 2018). In order to promote tobacco smoke-induced COPD lung pathology, NE and MMP-12 (macrophage elastase) work in concert. MMP-12 breaks down the NE inhibitor α-1 antitrypsin (Hautamaki et al., 1997) and NE breaks down the MMP-12 inhibitor TIMP1 (Jacksonet al., 2010), leading to unrestrained protease activities. These results indicate that neutrophil elastase is a prospective target for the treatment of respiratory diseases, and that elastase inhibition will eventually contribute to the reduction of pathological and functional abnormalities.

The search for inhibitors that either directly inhibits the enzyme or prevent its release from neutrophils, using natural substances may be an interesting source. Phenolic compounds, flavonoids and tannins, present in plant extracts exhibit potent inhibitory capacity against human neutrophil elastase (Melzig et al., 2001). A well-known anti-inflammatory plant source is *Lonicera japonica* (Lee et al., 1998). Traditional medicine makes extensive use of the entire plant, including the leaves and blossoms, as an anti-inflammatory drug, particularly for the treatment of disorders affecting the upper airways. In ancient literatures, *L. japonica* is a component of numerous intricate prescriptions for lung inflammatory sickness. Its primary ingredients, iridoids and flavonoids, exhibit strong anti-inflammatory effect (Lee et al., 1995). Additionally, rats exposed to low doses of LPS experienced an inhibition of ALI caused by the alkaloid portions of *Alstonia scholaris* and *Aconitum tanguticum*. At modest doses, *Ginkgo biloba* leaf extract significantly reduced lung inflammation in LPS-induced ALI (Huang et al., 2013), and the leaves also have an anti-asthmatic action (Babayigit et al., 2009). Several plant extracts, including *Schisandra chinensis*, *Callicarpa japonica, Juglans regia*, *Azadirachta indica*, *Euterpe oleracea*, *Stemona tuberosa*, *Galla chinensis*, and *Cnidiummonnieri* were discovered to inhibit inflammatory changes in the lung when used during the COPD model developed using cigarette smoke (Qamar and Sultana, 2011; Koul et al., 2012; Moura et al., 2012; Kwak and Lim, 2014; Lee et al., 2014; Zhong et al., 2015). In Asia and Europe, bronchitis is widely treated with *Pelargonium sidoides* (Matthys and Funk, 2008), *Hedera helix* (Guo et al., 2006), and *Echinacea purpurea* (Sharma et al., 2006; Agbabiaka et al., 2008).

*S. apetala*, a fast-growing evergreen tree, can reach heights of up to 15 m, with some examples reaching 20 m, and have trunks that are 20–30 cm in diameter. The common local names for *S. apetala* are Keora (Bengali), Chipi (Marathi), and Khirwa (Oriya), MaramaMaram (Tamil), Kyalanki (Telugu) *etc.* Its fruits are edible and used to prepare pickles and juices. It is rich in polyphenols like flavonoids, tannins and are mostly found in the Indian subcontinent along the coastal lines of the Bay of Bengal in India and Bangladesh, parts of Sri Lanka, Myanmar and southern China. The various parts of the plant are used for different pharmacological activities. For a long period, the fruits of the plant are used to treat various diseases. Interestingly, traditionally the fruits help in the prevention of wheezing and cough due to lung infection and asthma and are used as a folklore medicine for symptomatic treatment. There is no scientific study about it, establishing its bio-mechanism and phytochemicals responsible for inhibiting elastase. In the present study, we reported for the first time that the fruit extracts could be a potential inhibitor of human neutrophil elastase tested *in situ*, *in vitro* and *in vivo* elastase-induced lung injury model.

We collected fresh fruits of *S. apetala* and extracted them with the help of hydroalcoholic extract (Ethanol: Water). We tested the anti-elastase activity with the extract and found that it is exerting inhibitory activity against human neutrophil elastase at various concentrations (Figure 3A). To find the IC_50_ value of the extract we employed a series of dilutions of the fruit extract and found that the IC_50_ is 0.3718 ng/ml as compared with the control drug Sivelestat (Table 5), which is better as compared to IC_50_ of some common herbal extracts with anti-elastase activities like that of *Centenella asiatica* (14.547 μg/ml) (Nema et al., 2013), polyphenols from grape pomace extract (14.7 μg/ml) (Wittenauer et al., 2015), methanolic (ME) extracts from *Washingtonia filifera* (10.76 μg/ml) (Era et al., 2021) and *Tagetes erecta* Linn flower extract (4.13 μg/ml) (Maity et al., 2011).

Data analysis methods that linearize fundamentally nonlinear relationships are frequently used to characterize the inhibition process (either uncompetitive, noncompetitive or competitive). To ascertain the mechanism by which the fruit extract is binding to the enzyme we performed a substrate dilution assay and found that the extract follows a competitive mode to inhibit the human elastase (Figure 3B), as shown in Lineweaver-Burk plot. Several plant triterpenes (ursolic, oleanolic and 18β-glycyrrhetinic acids) show competitive inhibition (Ríos et al., 2000).

To clarify the impact of elastase on lung epithelial cell line, we also carried out various cell-based experiments. Under fluorescence microscopy, it was found that fruit extract was effective against cell apoptosis and desquamation. It was also noted that extracts and sivelestat therapy inhibited the rounding of the cell that is induced by elastase. (Figure 4A). Cell detachment assay was carried out to check how fruit extracts were able to prevent the shedding of cell as compared to the control drug in presence of neutrophil elastase (Figure 4B). These findings were in line with the already reported effects of Thai herbal extract on lung cancer treatment (Poofery et al., 2020). Cell viability was tested using an MTT assay. Both fruit extracts and sivelestat prevented the viability of the cells as a result of elastase on its own. It is interesting to note that both the test and control inhibitor itself has no cellular toxicity (Figure 4C). Since some of the previous studies have reported of toxicity in herbal extracts (Nemati et al., 2013), fruit extracts of *S. apetala* are indeed good source of compounds which can later be isolated and formulated into semi synthetic molecule.

Reports have emerged about the possible detachment of lung cells due to less elasticity and decrease in the adhesion molecules like ICAM-1. Serum ICAM-1 (soluble) are known marker of inflammation and was reported to be higher in COPD patients and active smokers (Grzelewska-Rzymowska and Pietrzkowicz, 2004; Lopez-Campos et al., 2012) and the effect of cigarette smoke on cultured human bronchial epithelial cells resulted in increased sICAM levels. ICAM-1 is released in the blood stream of COPD patients (Liu et al., 2018) as sICAM and serum levels of ICAM-1 were also elevated in patients with vascular inflammation (Jublanc et al., 2011). Together with the above observations and reports of neutrophil infiltration in COPD resulting in ICAM-1 upregulation in epithelial cells, we hypothesised that human neutrophil elastase might have an effect on ICAM-1 which in turn is released in the serum of patients as soluble ICAM (sICAM). So we checked the effects of SAM on the ICAM-1 expression in lung epithelial cells and subsequent released sICAM levels in culture media after elastase treatment in cells as compared to the control drug. We observed that the expression of ICAM-1 increases in the first hour of treatment which is reversed by the addition of SAM or Sivelestat (Figure 5A). These results are in accordance with a previously reported study on endothelial cells where elastase increased ICAM-1expression (Ishihara et al., 2006). Interestingly ICAM-1 expression decreased after 2 h of elastase treatment (Figures 5B,C) suggesting either a release into extracellular environment or breakdown of the protein. After further investigation we found that there is an increase in sICAM levels in cell culture media after 2 h of elastase treatment indicating the release of cellular ICAM-1 into cell culture media as soluble ICAM (Figure 5E). Thus epithelial cells shed the excess of ICAM-1 in circulating blood or extracellular fluid, produced as a result of elastase activity, possibly to reduce their susceptibility to toxicity. The increase in sICAM levels after 1 h as compared to control was non-significant suggesting that though morphological changes have started to occur ICAM-1 is not released into media as sICAM. Interestingly SAM when co-treated with elastase stopped the release of sICAM, thereby preventing lung cells to detach away as similar to control drug Sivelestat.

The LC-MS/MS profiles corroborate well with the HPLC spectrum and support the presence of gallic acid ellagic acid, along with other phenolic compounds in the extract (Table 4). This establishes that *S. apetala* fruit extract attenuated the damage caused by HNE in COPD disease, polyphenols such as Gallic acid, Ellagic acid, Luteoline, Ursolic acid and further Sonneradon A and Sonneradon C *etc.* may play a potential role in the inhibition of HNE. out of the ten compounds identified by LC-MS/MS, ellagic acid and gallic acid are known to reduce emphysema in mice and rats. Studies conducted by various groups showed that ellagic acid can prevent inflammation and oxidative stress in elastase-induced emphysema model in rat (Mansouri et al., 2020; Sohrabi et al., 2021) and reduce oxidative stress in pulmonary cells induced by elastase (Bayati et al., 2022)**.** Previous reports also show that gallic acid can ameliorate COPD linked lung inflammation and emphysema in mice (Singla et al., 2020; Wu et al., 2022). It can also prevent COPD-associated exacerbation in mice (Singla et al., 2021). Hence we checked for the inhibitory effects of ellagic acid and gallic acid on neutrophil elastase. our results showed that ellagic acid and gallic acid can inhibit human neutrophil elastase with an IC_50_ of 841 ng/ml and 962 ng/ml respectively *in situ* (Table 6) and prevents cellular morphological changes induced by elastase on lung epithelial cells *in vitro* (Figure 6). So ellagic acid and gallic acid are among the bioactive compounds present in *S. apetala* fruit extracts which contribute towards the inhibitory effects of the extract against human neutrophil elastase. The synergistic effects of ellagic acid and gallic acid along with other compounds contribute to the potent inhibitory effects with the IC_50_ (Table 5) comparable to the standard Sivelestat. Thus our study showed that ellagic acid and gallic acid strongly inhibits human neutrophil elastase which may ameliorate the emphysematous features in elastase induced mice as reported before.Figures 7A,B from studies in mice with an elastase-induced lung damage model demonstrate a considerable improvement in lung deterioration. Here, we found that at 1 mg/kg/day; 10 mg/kg/day and 100 mg/kg/day fruit extract of *S. apetala* provides shield to the damage caused by elastase and can be compared to other studies related to COPD where at 10 mg/kg/day, Paeonol, a key component of *Paeonia suffruticosa*, specifically reduced inflammation of the lungs in mice with a COPD model (Liu et al., 2014). When administered orally, petroleum ether fraction of *Viola yedoensis* and extracts of *Taraxacum officinale* exhibited strong inhibitory efficacy against LPS-induced lung inflammation at low doses of 3 mg/kg (Liu et al., 2010; Li et al., 2012). Thus, the fruit extracts of *S. apetala* like many other herbal formulations have a good amount of inhibitory potential against elastase-induced mice model of lung diseases. As lung damage in the form of alveolar collapse is associated with an increase in the formation of pro-Inflammatory mediators, Expectedly, we have observed an increase in the levels of key pro-inflammatory mediators like CXCL1/KC, murine IL-8 homologue, and IL-17A; both are essential in causing alveolar damage by recruiting neutrophils, in the lung homogenates of mice. However, SAM treatment had reduced the levels of these IL-17A and KC. It is important to note that IL-8 and IL-17A have a high correlation with sICAM-1 and disease severity in COPD patients (Hollander et al., 2007). In a corroborative manner, we also found a reduction in the levels of sICAM-1 in *in-vitro* studies with SAM treatment. So we checked for the levels of sICAM in BAL fluid and serum of control and treated mice. We found an increase in sICAM levels both in BAL fluid and serum suggesting that elastase increased the expression of ICAM-1 in lung cells which gradually released into circulation. SAM and Sivelestat significantly prevented the elastase induced release of ICAM-1 in BAL and serum.

Recent reports suggest that neutrophil elastase inhibitors can be used in patients suffering from deadly COVID-19 (Mohamed et al., 2020). Therefore, SAM can be further studied for a potential therapeutic candidate to combat the disease which took the lives of 6.5 million people in early 2020 across the world (https://covid19.who.int/).

To conclude, fruit extract of *S. apetala* (SAM) has a potential inhibitory effect against human neutrophil elastase and exerts its action competitively. It also protects human lung epithelial cells from any elastase-induced toxicity and morphological changes exerting its pharmacological effects by preventing elastase from inducing ICAM-1 levels *in vitro* and also reverses emphysematous changes of elastase-induced mice indicating that it can act as a protective barrier inside lungs in diseased conditions. Further studies established that gallic acid and ellagic acid are present in the extract as bioactive compounds, where ellagic acid is comparably more potent inhibitor of human neutrophil elastase.

## Data Availability

The raw data supporting the conclusion of this article will be made available by the authors, without undue reservation.

## Abbreviations

ALI, Acute Lung Injury; ARDS, Acute Respiratory Distress Syndrome; BAL, Broncho alveolar Lavage; COPD, Chronic Obstructive Pulmonary Disease; HNE, Human Neutrophil Elastase; ICAM-1, Intercellular Adhesion Molecule 1; LPS, Lipopolysaccharide; MLI, Mean Linear Intercept; MMP, Matrix Metalloproteinase; MPO, Myeloperoxidase; NE, Neutrophil Elastase; NET, Neutrophil Extracellular Trap; RFU, Relative Fluorescence Unit; TIMP, Tissue inhibitors of metalloproteinases; SAM, *S. apetala* Buch.-Ham. fruit extract.
