# Corrigendum: Standardised *Sonneratia apetala* Buch.-Ham. fruit extract inhibits human neutrophil elastase and attenuates elastase-induced lung injury in mice

**DOI:** 10.3389/fphar.2023.1175091

**Published:** 2023-04-13

**Authors:** Sayantan Sengupta, Nipun Abhinav, Sabita Singh, Joytri Dutta, Ulaganathan Mabalirajan, Karthigeyan Kaliyamurthy, Pulok Kumar Mukherjee, Parasuraman Jaisankar, Arun Bandyopadhyay

**Affiliations:** ^1^ Cardiovascular Disease and Respiratory Disorders Laboratory, Cell Biology and Physiology Division, CSIR-Indian Institute of Chemical Biology, Kolkata, India; ^2^ Department of Natural Products, National Institute of Pharmaceutical Education and Research (NIPER), Kolkata, India; ^3^ Molecular Pathobiology of Respiratory Diseases Laboratory, Cell Biology and Physiology Department, CSIR-Indian Institute of Chemical Biology, Kolkata, India; ^4^ Academy of Scientific and Innovative Research (AcSIR), Ghaziabad, India; ^5^ Central National Herbarium, Botanical Survey of India, Howrah, India; ^6^ Institute of Bioresources and Sustainable Development, Imphal, India; ^7^ Laboratory of Catalysis and Chemical Biology, Department of Organic and Medicinal Chemistry, CSIR-Indian Institute of Chemical Biology, Kolkata, India

**Keywords:** *Sonneratia apetala* Buch.-Ham., neutrophil elastase, lung injury, COPD, ellagic acid, LC-MS/MS, HPLC, emphysema

In the published article, there was an error in **Figure 5A** as published. The bottom 3 rows of iCAM 1 panel (red colour) are inadvertently swapped with WGA panel (green colour)**.** The corrected **Figure 5** and its caption appear below.

**FIGURE 5:**
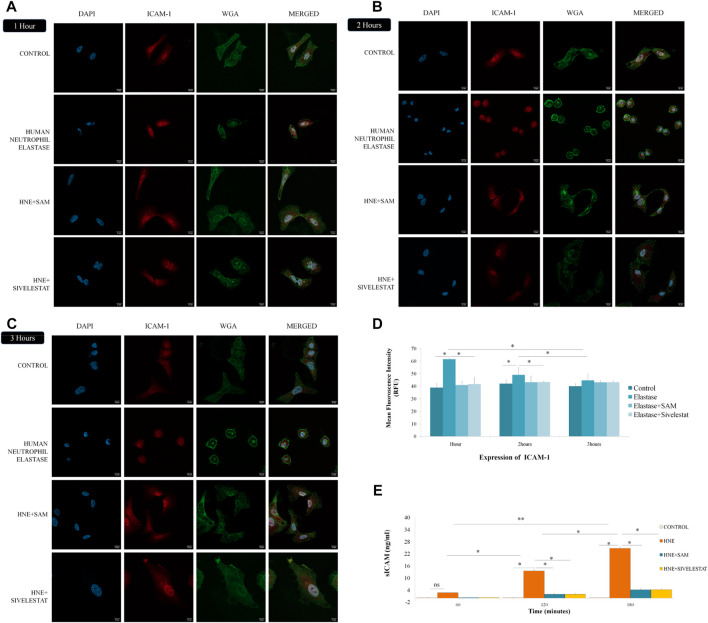
*S. apetala* fruit extracts ameliorates ICAM-1 release induced by neutrophil elastase *in vitro* **(A)** The ICAM-1 expression in lung epithelial cells was significantly increased after 1 h of elastase treatment **(B)** Increase in ICAM-1 expression after 2 h with deformation in cell shape was restored in SAM/Sivelestat groups **(C)** Complete rounding of elongated epithelial cells with increased ICAM-1 expression reversed by concomitant treatment of SAM/Sivelestat **(C)** as assessed under Confocal microscope at ×60 magnification stained with DAPI (blue). Anti-ICAM-1 antibody (red), and WGA (green) **(D)** Mean fluorescence intensity (MFI) was calculated for ICAM-1 expression at each time point **(E)** sICAM levels were measured after 1, 2 and 3 h of elastase treatment by ELISA. Significant increase in sICAM levels were observed after second and third hour which was reversed by treatment with SAM/Sivelestat.

The authors apologize for this error and state that this does not change the scientific conclusions of the article in any way. The original article has been updated.

