# Supplementary material for: Standardised Sonneratia apetala Buch.-Ham. fruit extract inhibits human neutrophil elastase and attenuates elastase-induced lung injury in mice
Source: Front Pharmacol. 2022 Dec 7;13:1011216. doi: 10.3389/fphar.2022.1011216 (PMC9768866; doi:10.3389/fphar.2022.1011216)
Supplement: Supplementary file 1 [file DataSheet1.pdf]

# *Supplementary Material*

## Standardised *Sonneratia apetala* Buch.-Ham. fruit extract inhibits human neutrophil elastase and attenuates elastase-induced lung injury in mice

Sayantan Sengupta[1, †], Nipun Abhinav[2,†], Sabita Singh[3, 4], Joytri Dutta[3, 4], Ulaganathan Mabalirajan[3, 4], Karthigeyan Kaliyamurthy[5], PulokMukherjee[6], Parasuraman Jaisankar[4, 7,*], Arun Bandyopadhyay[1, 4,*]

[1]Cardiovascular Disease and Respiratory Disorders Laboratory, Cell Biology & Physiology Division, CSIR-Indian Institute of Chemical Biology, Kolkata

[2]Department of Natural Products, National Institute of Pharmaceutical Education and Research (NIPER), Kolkata

[3]Molecular Pathobiology of Respiratory Diseases Laboratory, Cell Biology & Physiology Department, CSIR-Indian Institute of Chemical Biology, Kolkata

[4]Academy of Scientific and Innovative Research (AcSIR), Ghaziabad, India

[5]Central National Herbarium, Botanical Survey of India, A.J.C.B. Indian Botanic Garden, Howrah, India

[6]Institute of Bioresources and Sustainable Development, Imphal

[7]Laboratory of Catalysis and Chemical Biology, Department of Organic and Medicinal Chemistry, CSIR-Indian Institute of Chemical Biology, Kolkata

[†] These authors contributed equally to this work

**Corresponding authors:**

* Dr. Parasuraman Jaisankar (email: jaisankar@iicb.res.in)

* Dr. Arun Bandyopadhyay (email: arunb@iicb.res.in)

CSIR-Indian Institute of Chemical Biology,

4 Raja S. C. Mullick Road, Jadavpur,

Kolkata 700032, West Bengal, India

# Table of Content


| Section | Subsection | Description | Page no. |
|---|---|---|---|
| **1** | **Supplementary Figures** | | |
| | **1.1** | LC-MS/MS profiling of SAM extract | **3-24** |
| **2** | **HPLC method development for simultaneous detection of Gallic acid and ellagic acid in *S. apetala* fruit extract (SAM)** | | |
| | **2.1** | Linearity and Sensitivity (Gallic acid) | **25-28** |
| | **2.2** | Linearity and Sensitivity (Ellagic acid) | **29-31** |
| | **2.3** | Accuracy (Gallic acid) | **32-33** |
| | **2.4** | Accuracy (Ellagic acid) | **33-34** |
| | **2.5** | Intra-day and Inter-day Precision (Gallic acid) | **35-38** |
| | **2.6** | Intra-day and Inter-day Precision (Ellagic acid) | **39-41** |
| | **2.7** | Robustness | **42-45** |
| | **2.8** | HPLC chromatogram of S. apetala fruit extract (SAM) 5mg/ml (injection volume= 20µl) (n=3) | **46** |
| | **2.9** | Mouse treatment protocol | **46** |
| **3** | **Tables of peak area for HPLC standardization parameters** | | |
| | **3.1** | Table S1: Linearity and Sensitivity- Calibration curve for Gallic acid | **47** |
| | **3.2** | Table S2: Linearity and Sensitivity- Calibration curve for Ellagic acid | **47** |
| | **3.3** | Table S3: Accuracy: %Recovery study | **48** |
| | **3.4** | Table S4: Intra- day and inter-day variation in peak area | **49** |
| | **3.5** | Table S5: Robustness: Effect of change in Flow rate (FR) (to 0.6 ml/min) in Wavelength (WL) (to 255 nm) on Peak area of Gallic acid and Ellagic acid (n=3). | **50** |
| **5** | **References** | | **50** |

# 1 Supplementary Figures

## 1.1 LC-MS/MS profiling of SAM extract

From the LC-MS/MS spectrum of SAM (Figure 3), total of 10 compounds were identified using the previous literature, matching the obtained fragments in the LC-MS/MS of SAM. LC-MS/MS confirmed the presence of Gallic acid, Ellagic acid as well as other polyphenols, fatty esters and terpenoids. Identified compounds are enlisted in (table 2). The SAM sample was prepared by dissolving 5mg extract in LC-MS grade MeoH and filtered through 0.22µM filter. Injection volume was 5µl. The LC-MS was equipped with Hypersil Gold $C_{18}$ column (diameter 100 × 2.1 mm, particle size1.9 µm.). LC analysis used gradient of solvent A (acetonitrile + 0.1% formic acid) and solvent B (HPLC grade $H_2O$ + 0.1% formic acid). The analyzer was set to positive mode, capillary temperature set to 320ºC, source voltage was 5.00 kV, Capillary voltage 45.00 V, Tube Lens Voltage (V)=110.04. MS Run Time (min): 32.00. Data analysis was performed in Thermo Scientific Xcalibur™ Software.

### 1.1.1 LC-MS profile of *S. apetala* fruit extract (SAM)

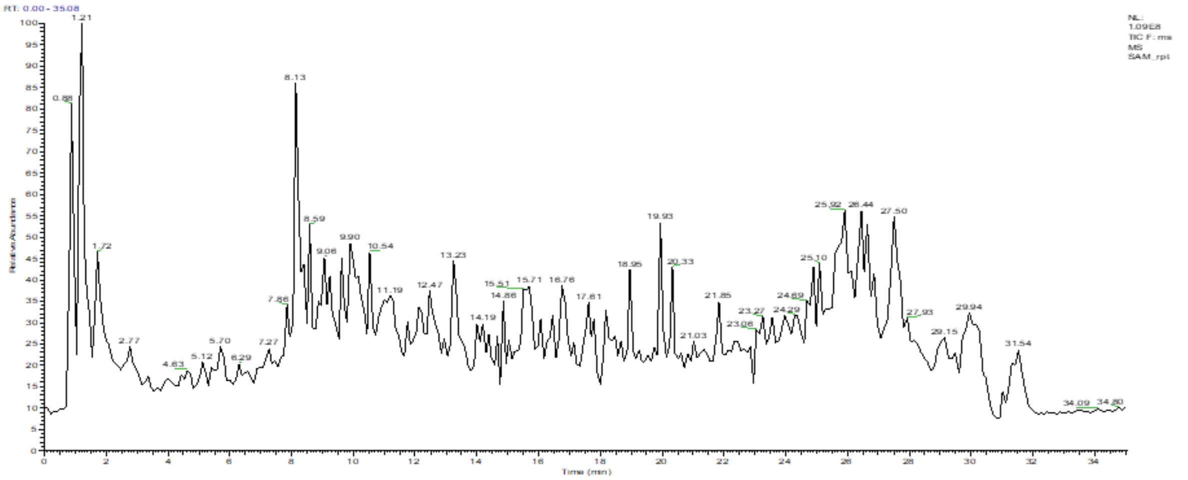


The following compounds were identified from LC-MS/MS spectrum after searchin and comparing the MS and $MS^2$ fragments with the literature.

| S.N. | *m/z* $[M+H]^+$ | MS/MS fragments | Compound identified |
|---|---|---|---|
| **1** | 295.23 | 263.16, 255.15, 236.16, 221.09 | 8,11-Octadecadienoic acid, methyl ester |
| **2** | 455.22 | 478.30, 437.19, 427.08, 418.94, 395.25 | Stigmasta-5,22-dien-3-ol, acetate |
| **3** | 375.29 | 357.27, 343.18, 319.31, 251.17, 119.31 | Sonneradon A |
| **4** | 307.23 | 289.20, 275.13, 243.02, 151.11 | Sonneradon C |
| **5** | 277.13 | 259.13, 241.12, 221.07, 207.09, 175.02 | Ranuncoside |
| **6** | 171.03 | 143.01, 135.01, 125.85, 124.85, 113.84 | Gallic acid |
| **7** | 303.25 | 275.25, 257.06, 229.09, 215.08, 201.05 | Ellagic acid |
| **8** | 287.05 | 268.96, 244.99, 179.03 | Luteoline |

| | | | |
|---|---|---|---|
| **9** | 457.37 | 439.30, 411.24, 393.26, 275.16, 249.20, 217.16 | Ursolic acid |
| **10** | 413.38 | 395.35, 367.31, 297.20, 283.25, 255.16, 241.14, 201.17 | Stigmasterol |

### 1.1.2 *MS and MS/MS spectrum of 8,11-Octadecadienoic acid, methyl ester*

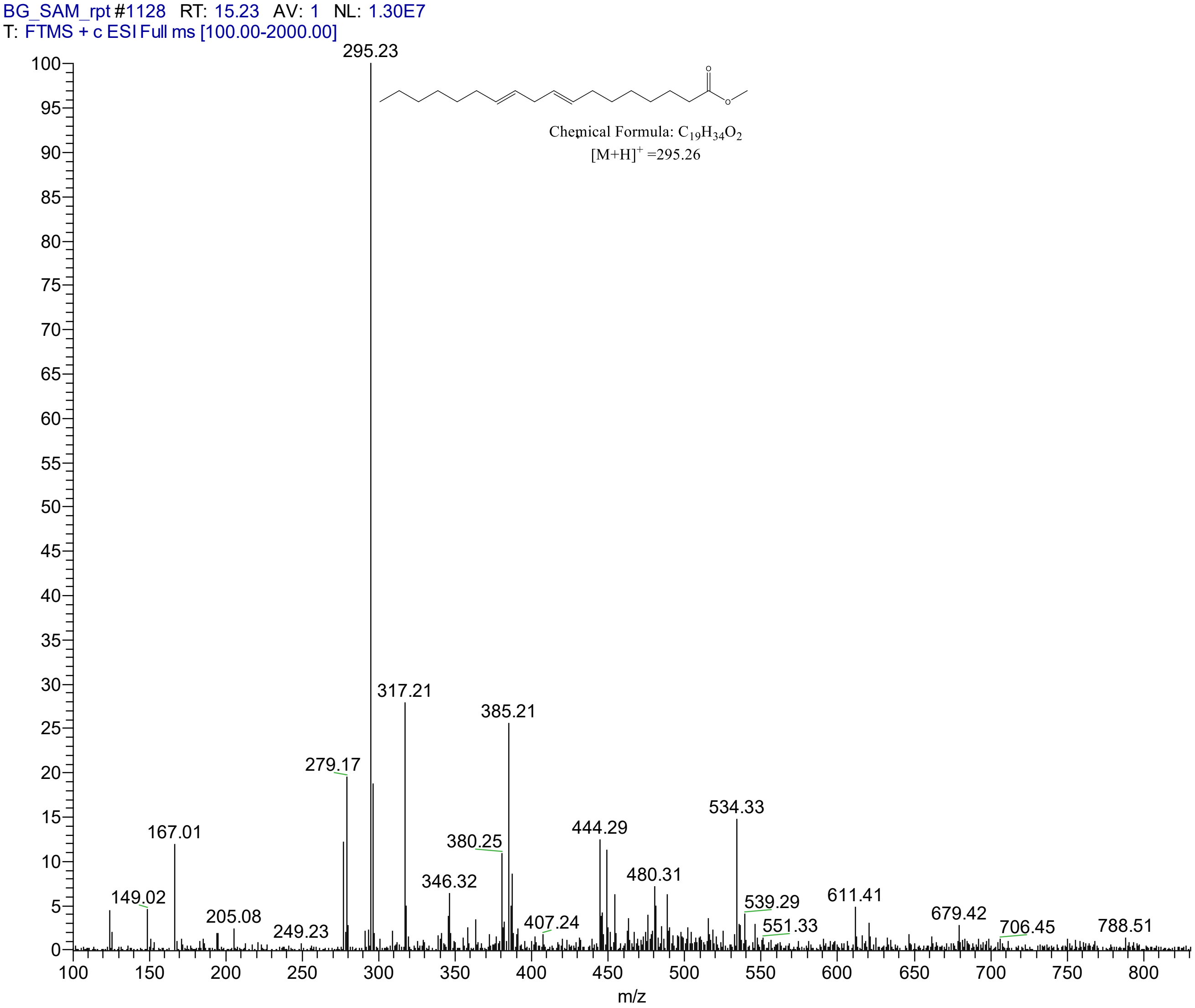

### *1.1.3 MS/MS spectrum of 8,11-Octadecadienoic acid, methyl ester*

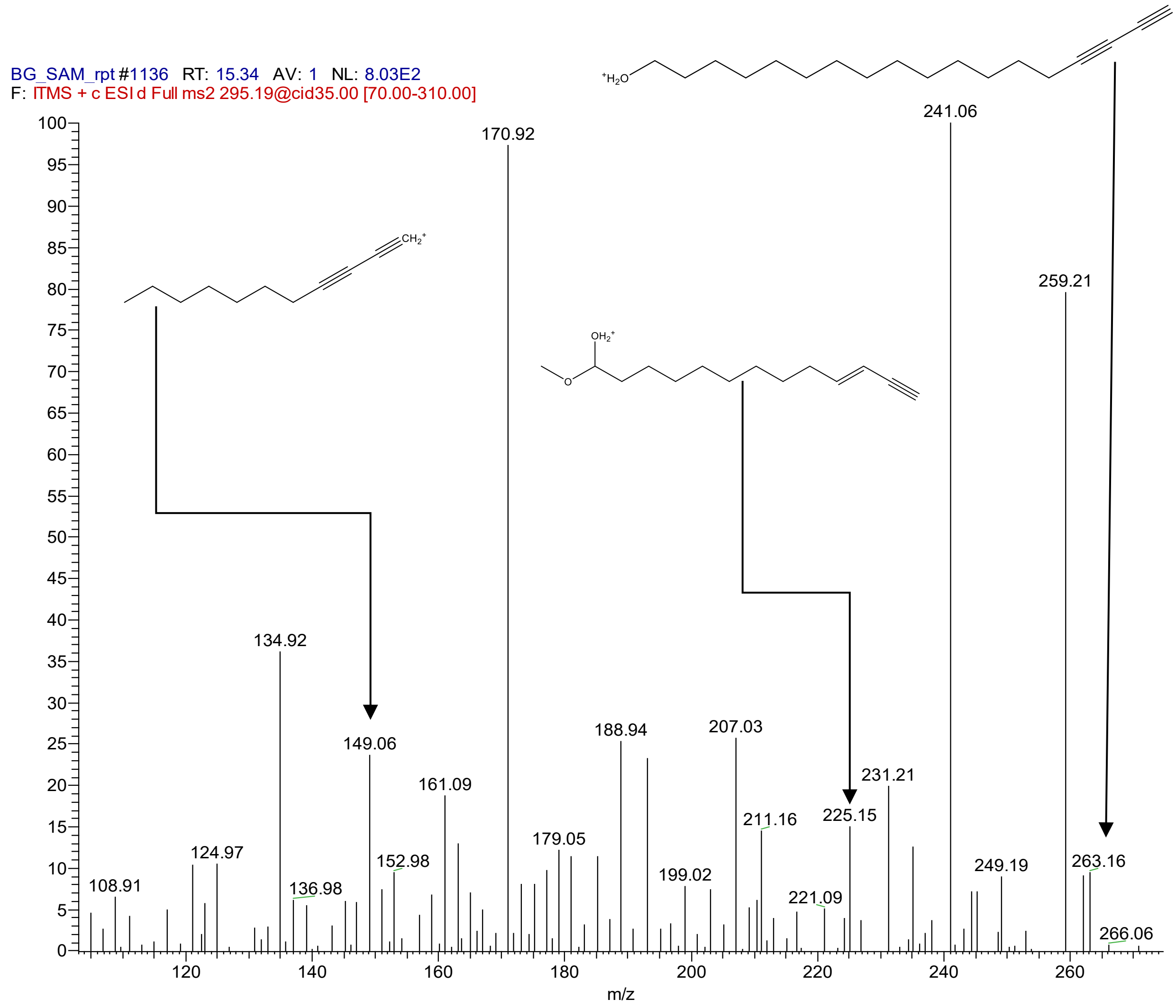


***Assigned MS and MS/MS fragments ion of 8,11-Octadecadienoic acid, methyl ester***

Molecular ion at *m/z*= 295.23 $[M+H]^+$. The loss of an O-$CH_3$ group from ester moiety resulted in fragment ion at *m/z*=263.16 $[M+H]^+$ and loss of $C_5H_9$ group resulted in fragment ion at *m/z* 225.15 $[M+H]^+$. Loss of $C_8H_{17}O_2$ group gave fragment ion ate 149.06. The compound was identified comparison with reported fragments in the literature (Hossain et al., 2017, Yang et al., 2020).

### 1.1.4 *MS spectrum of Stigmasta-5,22-dien-3-ol, acetate*

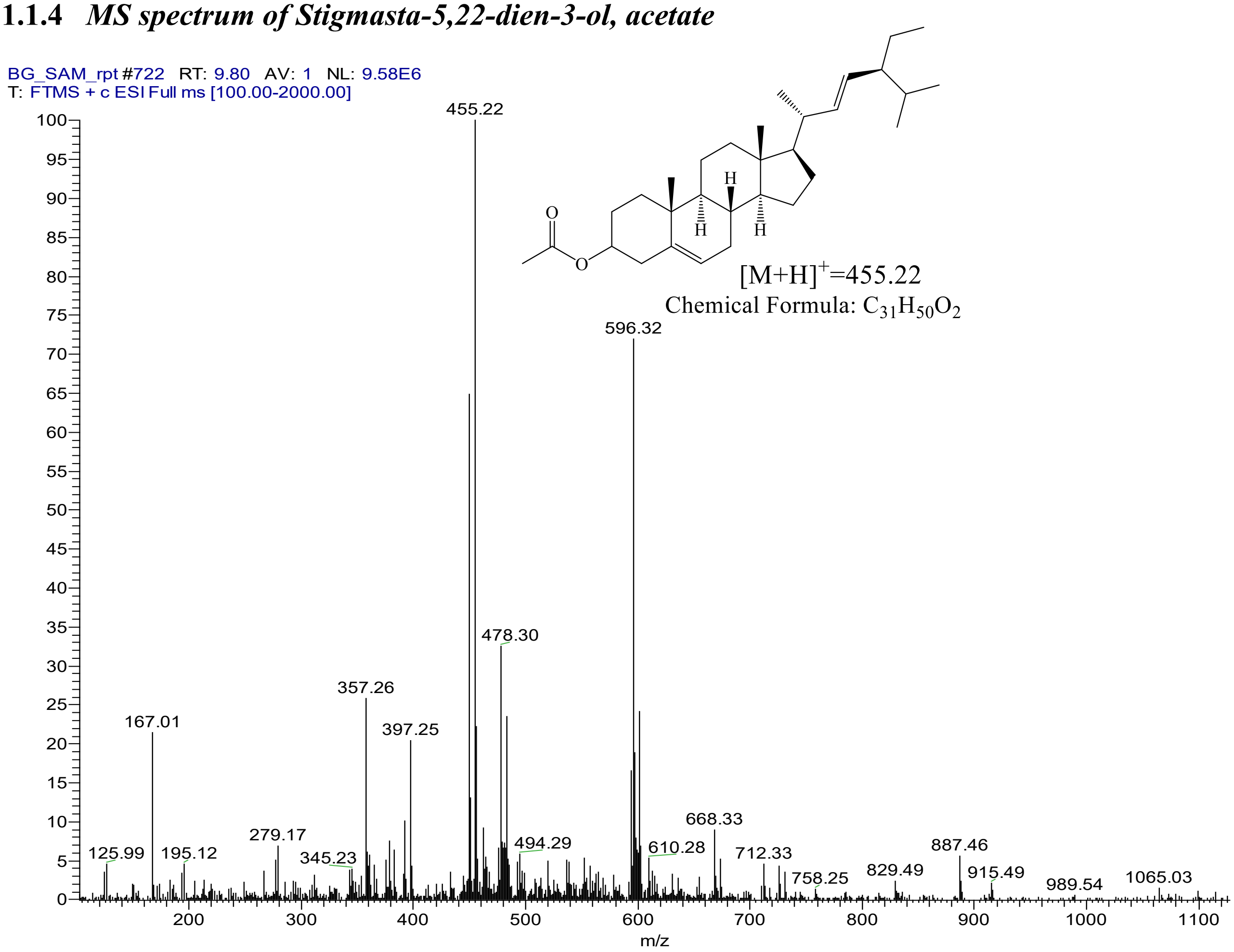

### *1.1.5 MS/MS spectrum of Stigmasta-5,22-dien-3-ol, acetate*

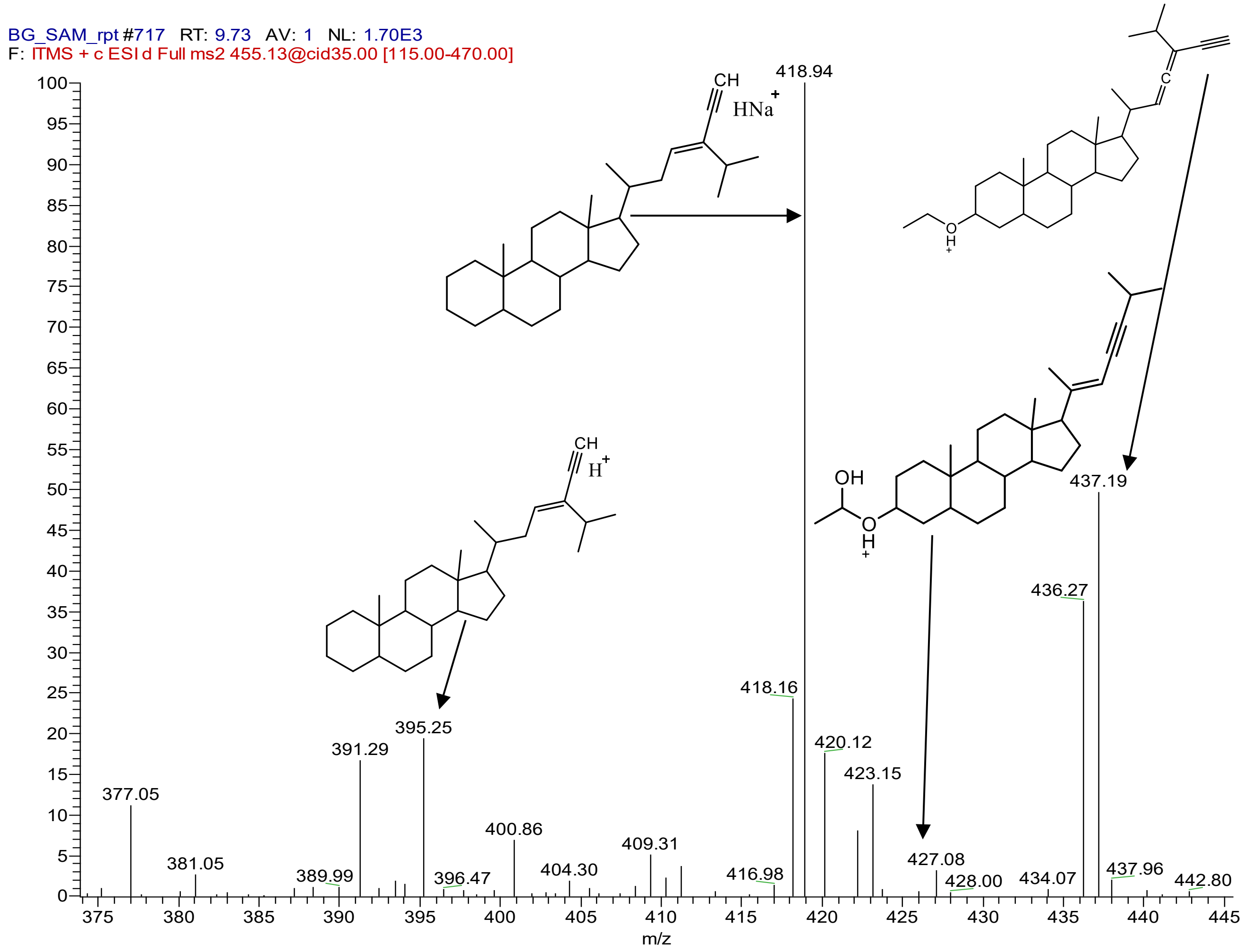


### *Assigned MS and MS/MS fragments ion of Stigmasta-5,22-dien-3-ol, acetate*

Molecular ion at *m/z*= 455.22 $[M+H]^+$ and at *m/z* 478.30 $[M+Na+H]^+$ . The loss of an OH group gave fragment ion *m/z* [437.19]. Loss of an ethene moiety resulted in fragment ion at *m/z* 427.08 $[M+H]^+$ and loss of Methyl acetate group resulted in fragment ion at *m/z* 418.94 $[M+Na]^+$ and at 395.25 $[M+H]^+$. The compound was identified by comparison with reported fragments in the literature (Chang and Kang, 2012, Hossain et al., 2017).

## 1.1.6 *MS spectrum of Sonneradon A*

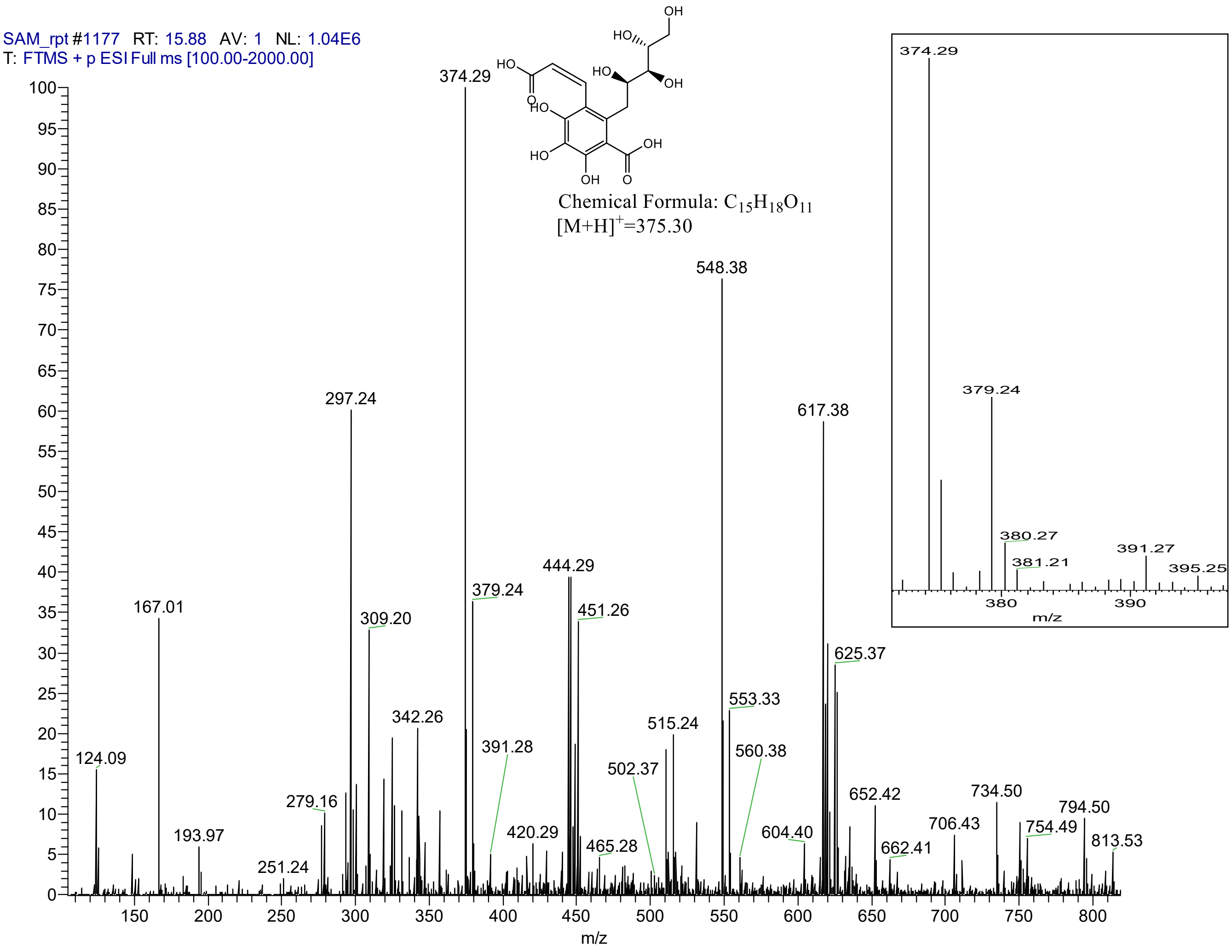

### 1.1.7 ***MS/MS spectrum of Sonneradon A***

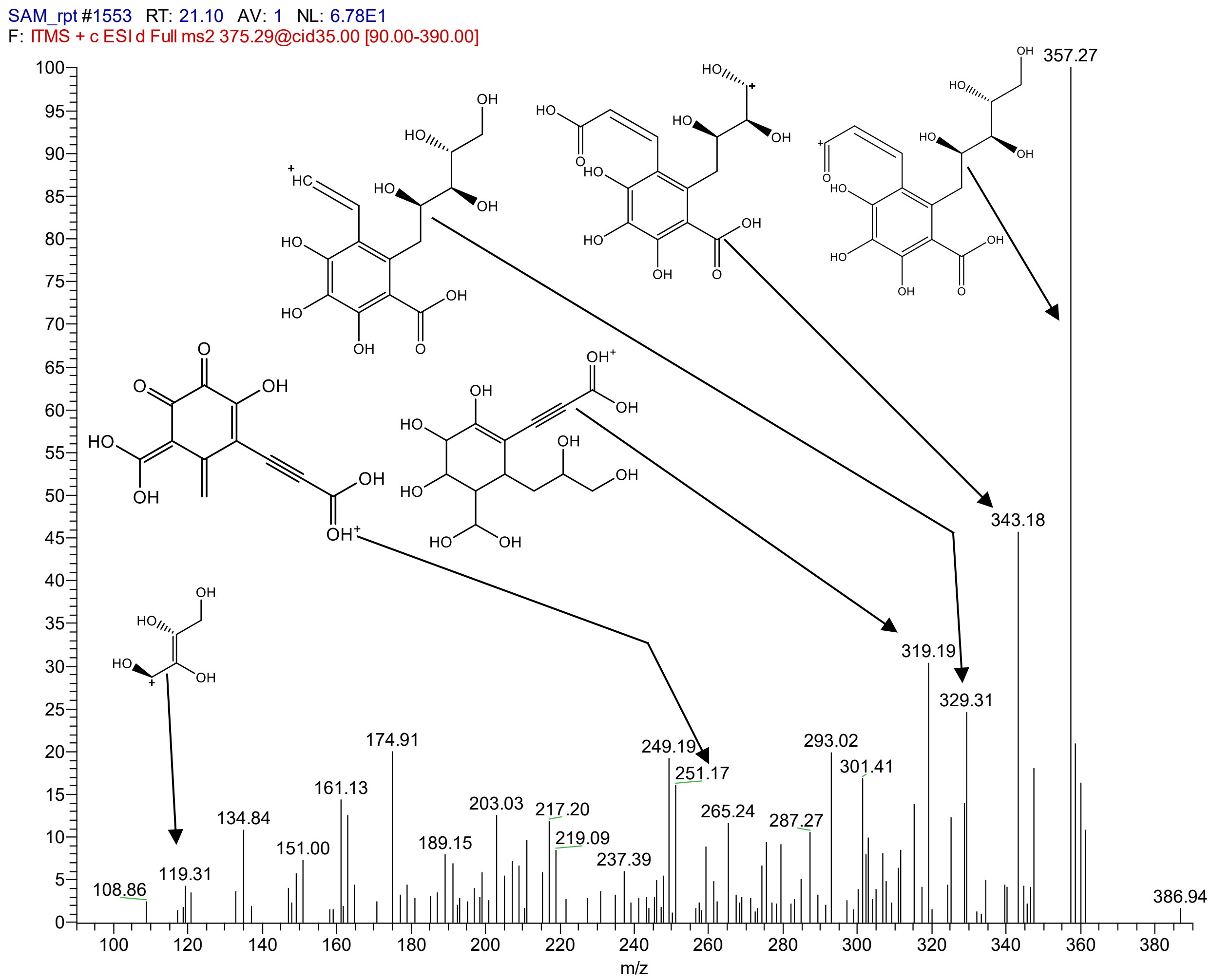


***Assigned MS and MS/MS fragments ion of Sonneradon A***

Molecular ion at *m/z*= 375.29 [M+H]$^+$ . The loss of an OH group gave fragment ion *m/z* 357.27[M+H]$^+$. Loss of an $CH_2OH$ group resulted in fragment ion at *m/z* 343.18 [M+H]$^+$ and loss of CHOH group resulted in fragment ion at *m/z* 319.31. Loss of tetrahydroxy-butene group from C-2 position resulted in fragment ion at *m/z*=251.17 [M+H]$^+$.The tetrahydroxy-butene fragment appeared at *m/z*=119.31[M=H]$^+$.The compound was identified by comparison with reported fragments in the literature (Yi et al., 2020, S et al., 2022).

## 1.1.8 *MS spectrum of Sonneradon C*

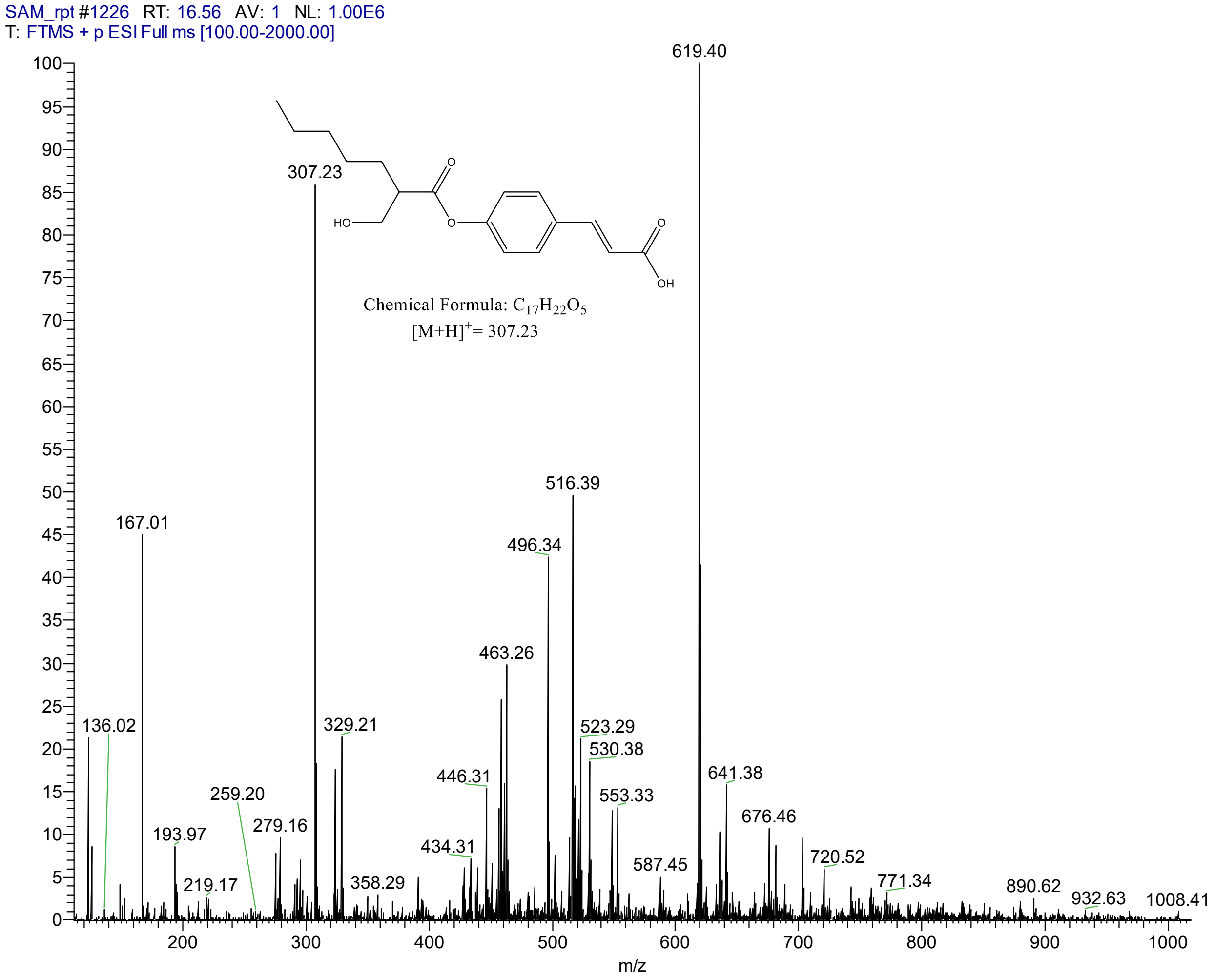

### *1.1.9 MS and MS/MS spectrum of Sonneradon C*

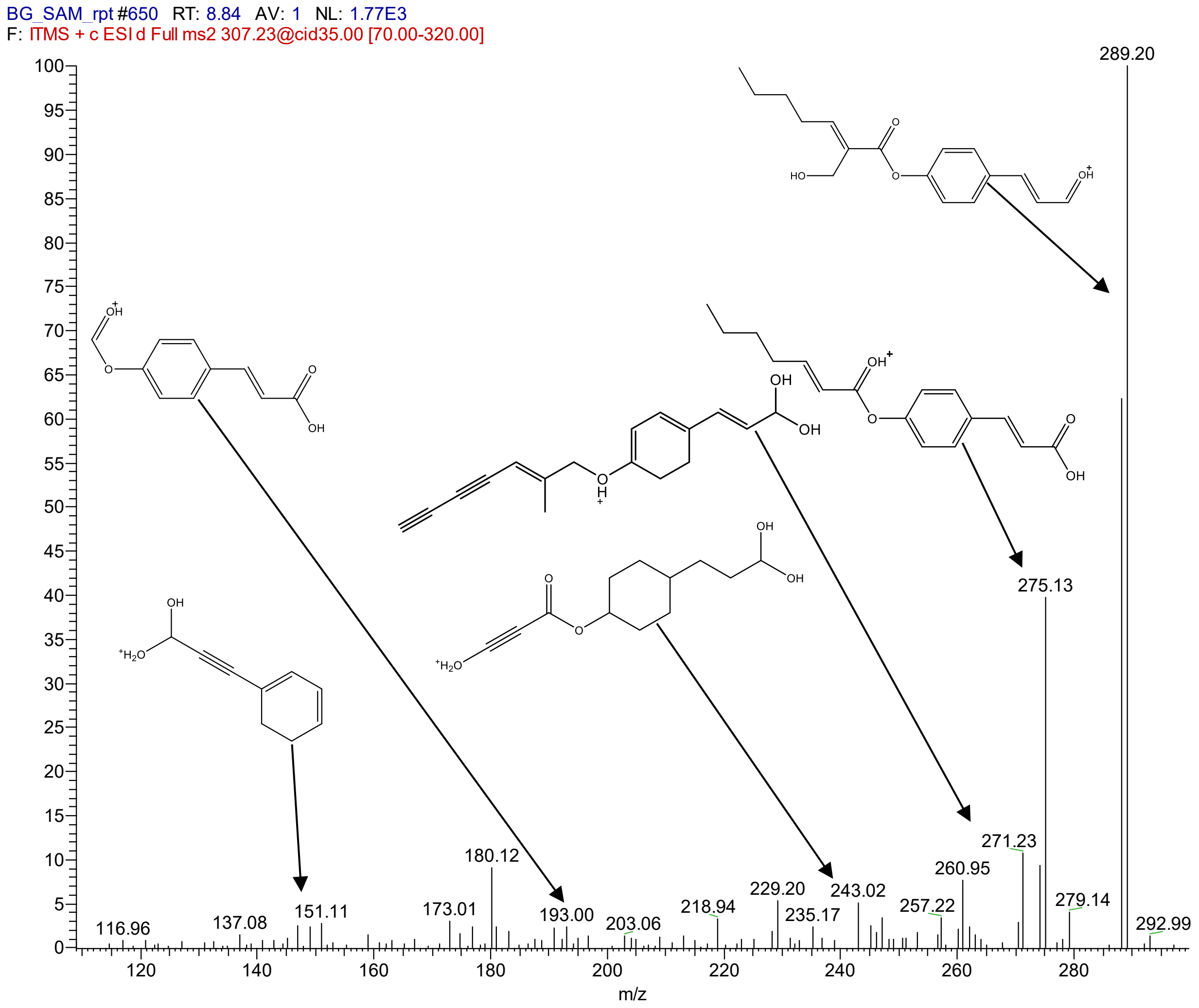


### *Assigned MS and MS/MS fragments ion of Sonneradon C*

Molecular ion at *m/z*= 307.23 [M+H]$^+$. The loss of an OH group gave fragment ion *m/z* 289.20 [M+H]$^+$. Loss of an OH group resulted in fragment ion at *m/z* 275.13 [M+H]$^+$.Loss of 2($H_2O$) molecules resulted in fragment ion *m/z* at 243.02. Fragmentation of C-O bond at C-1 resulted a fragment ion at *m/z* 151.11. The compound was identified by comparison with reported fragments in the literature (Yi et al., 2020, S et al., 2022).

### 1.1.10 *MS spectrum of Ranuncoside*

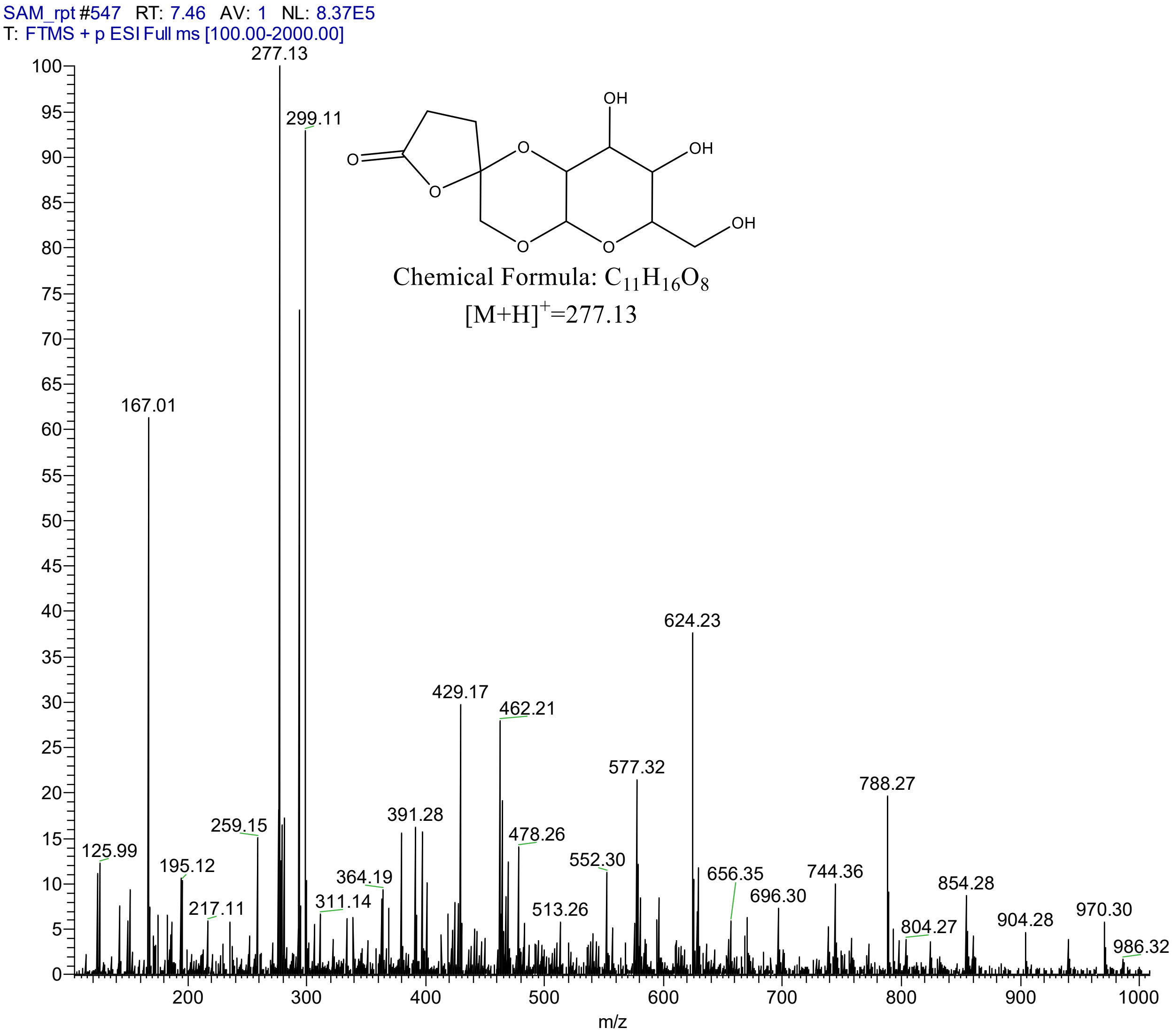

### 1.1.11 *MS/MS spectrum of Ranuncoside*

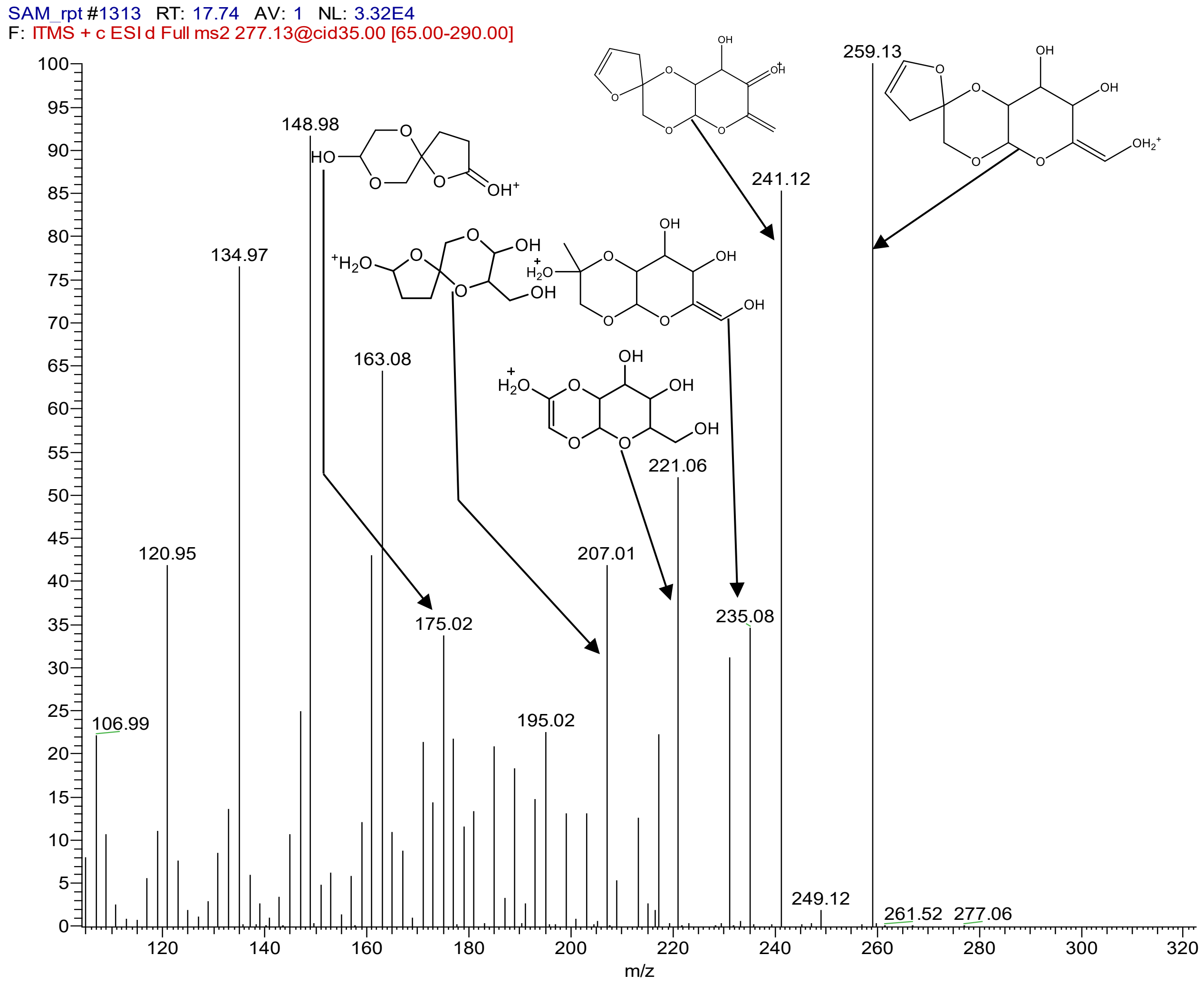


***Assigned MS and MS/MS fragments ion of Ranuncoside***

Molecular ion at *m/z*= 277.13 $[M+H]^+$. The loss of an OH group gave fragment ion at *m/z* 259.13 $[M+H]^+$. Loss of an $H_2O$ group resulted in fragment ion *m/z* at 241.12 $[M+H]^+$. Degradation of furan ring caused loss of ethane moiety resulted in fragment ion at *m/z* 235.08 $[M+H]^+$, which further degraded to give fragment ion at *m/z* 221.07 $[M+H]^+$due to loss of a methane group; subsequently loss of an OH group resulted in fragment ion at *m/z* 207.09 $[M+H]^+$. Degradation of pyran ring caused the loss of butanetriol group resulted in fragment ion at *m/z* 175.02 $[M+H]^+$ (Cuny and Klingler, 2022,Yi et al., 2020)

### 1.1.12 *MS spectrum of Gallic acid*

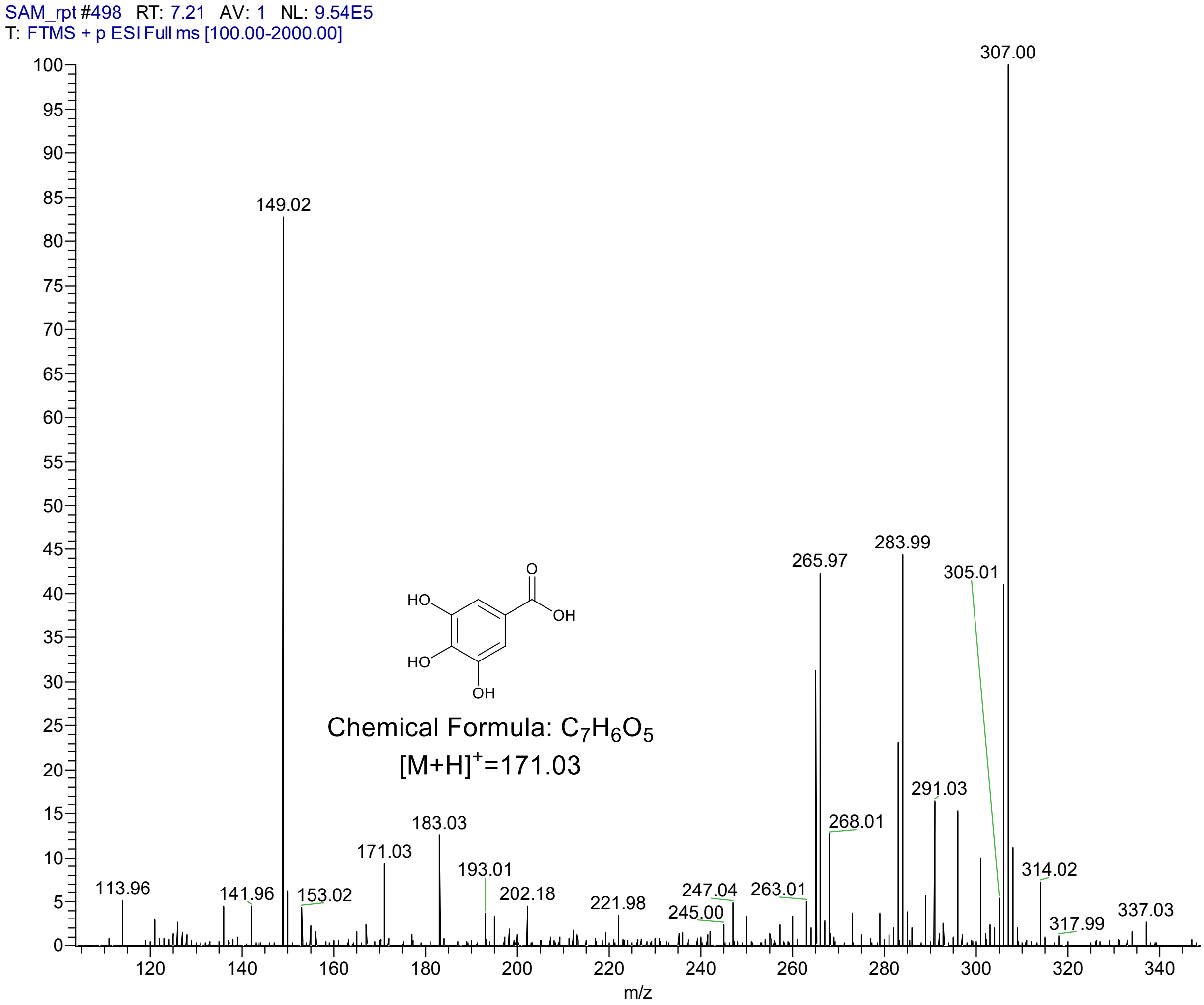

### 1.1.13 *MS/MS spectrum of Gallic acid*

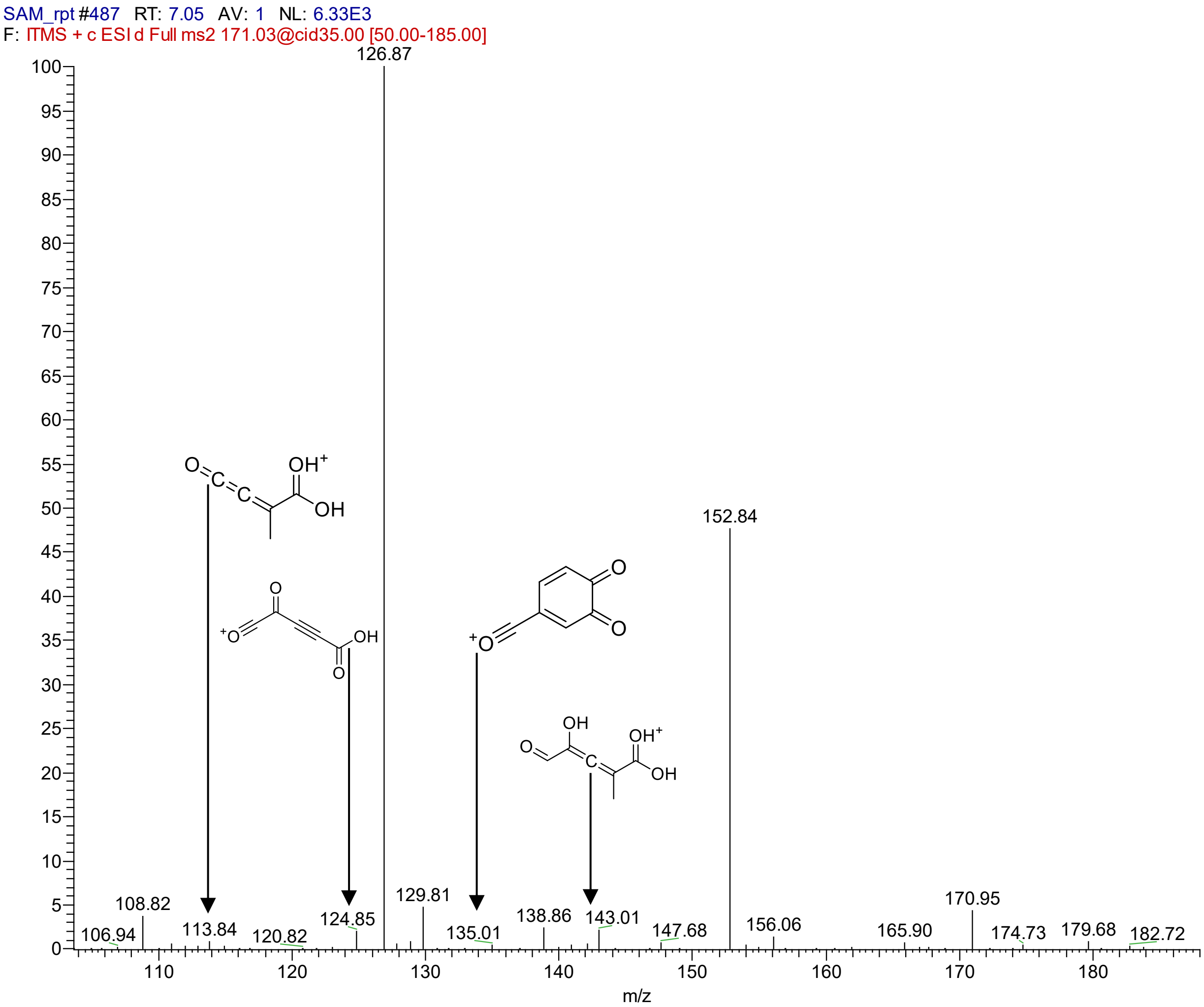


### *Assigned MS and MS/MS fragments ion of Gallic acid*

Molecular ion at *m/z*= 171.03 $[M+H]^+$. The loss of an OH group gave fragment ion *m/z* 153.02 $[M+H]^+$. Loss of an CHO group resulted in fragment ion at *m/z* 143.01 $[M+H]^+$. Simultaneous loss of an OH and $H_2O$ group resulted in fragment ion at 135.01 $[M+H]^+$. Loss of an $CH_3CHOH$ group from the aromatic ring resulted in fragment ion 124.85 *m/z* at $[M+H]^+$. Degradation of ring caused loss of COOCH moiety resulted in fragment ion at *m/z* 113.84 $[M+H]^+$ (Engström et al., 2015, Hossain et al., 2016).

## 1.1.14 *MS spectrum of Ellagic acid*

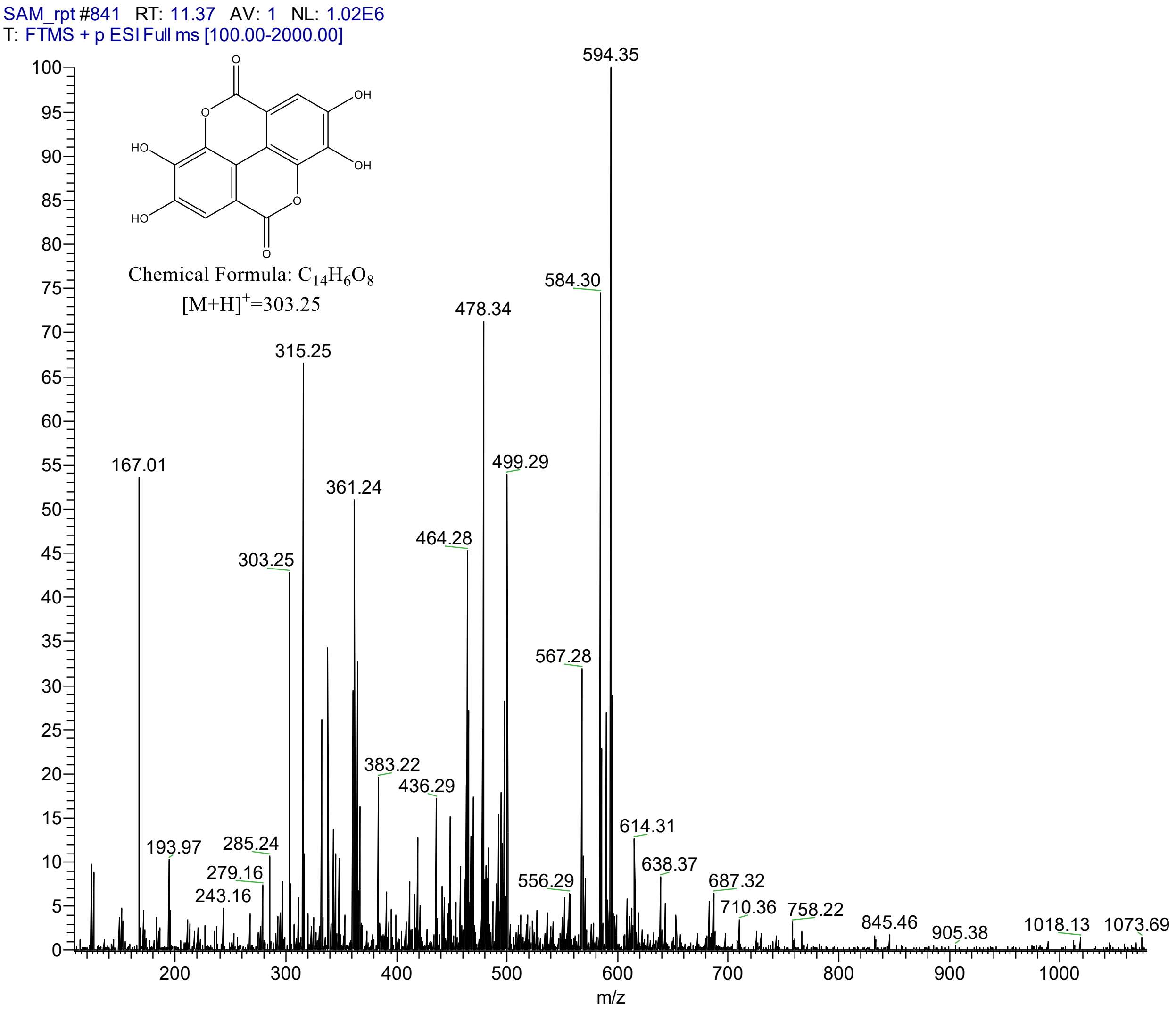

### 1.1.15 *MS/MS spectrum of Ellagic acid*

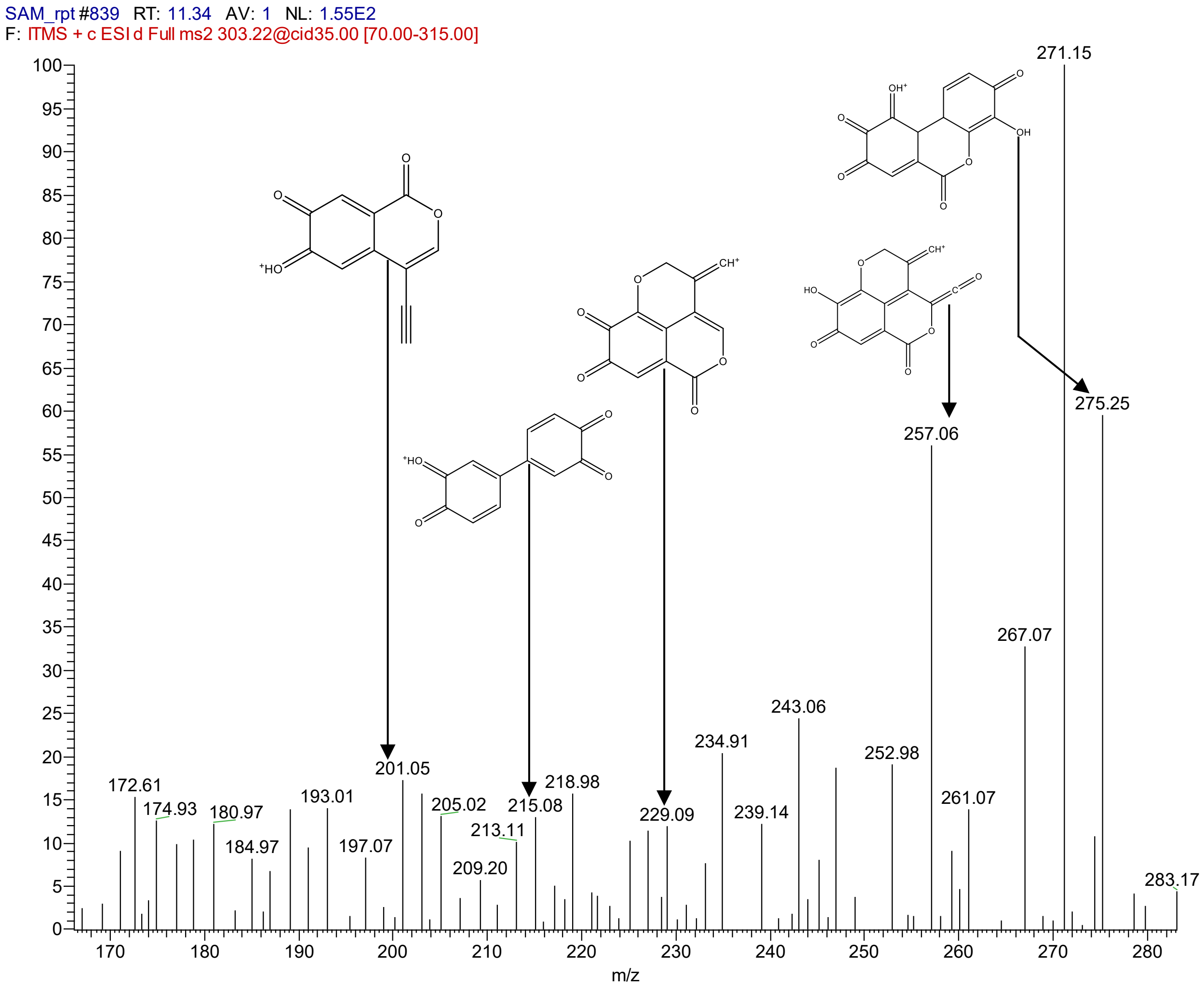


***Assigned MS and MS/MS fragments ion of Ellagic acid***

Molecular ion at *m/z*= 302.30 [M+H]$^+$. The loss of an CHO group gave fragment ion *m/z*=275.25 [M+H]$^+$. Loss of an COOH group resulted in fragment ion at *m/z* 257.06 [M+H]$^+$. Simultaneous loss of two OH and one CO group resulted in fragment ion at 229.09[M+H]$^+$. Degradation of two pyran rings resulted in fragment ion at *m/z* 215.08[M+H]$^+$. Chroman ring degradation resulted in fragment ion at *m/z*= 201.05[M+H]$^+$. (Singh et al., 2016, Engström et al., 2015)

### 1.1.16 *MS spectrum of Luteoline*

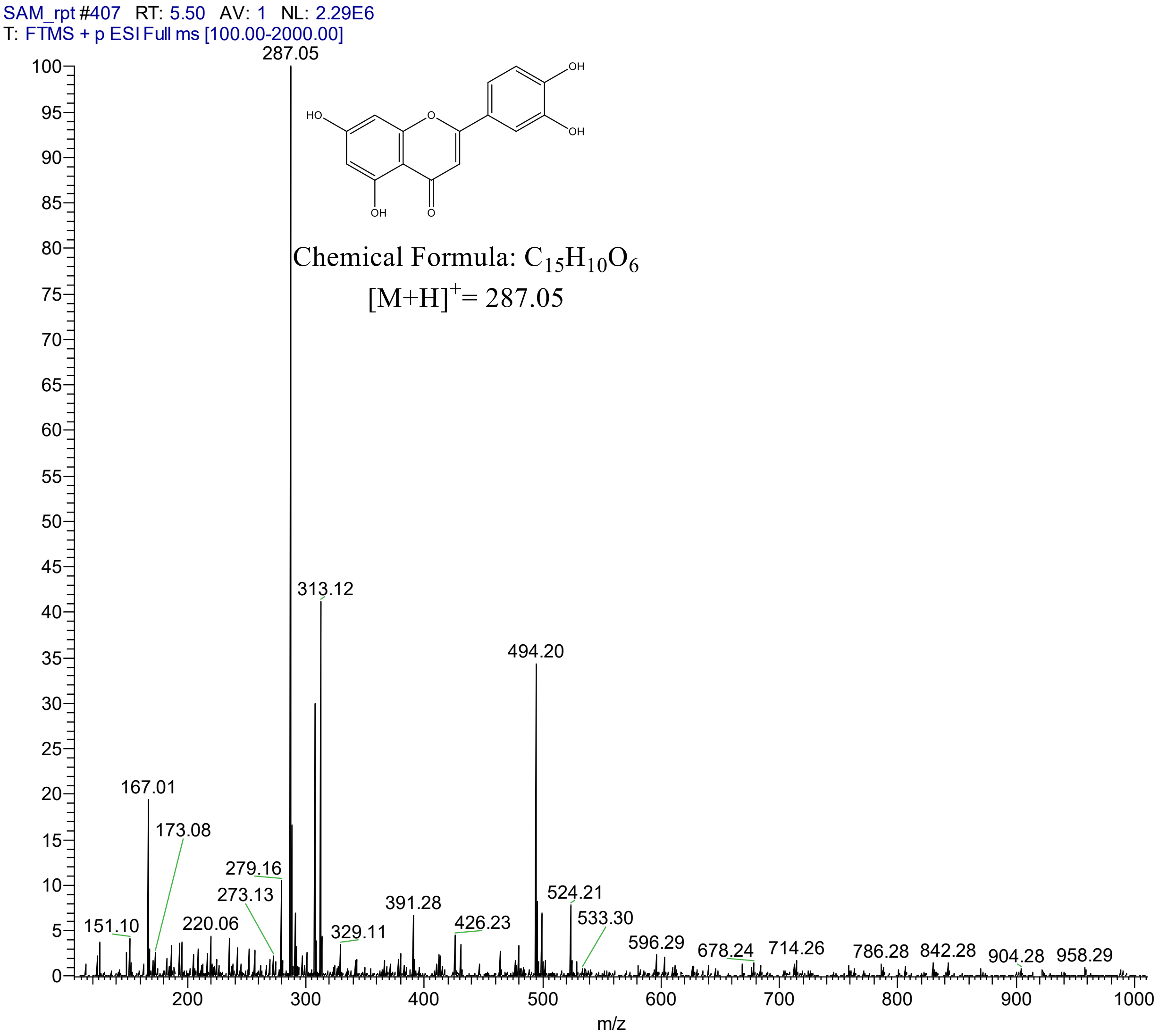

### 1.1.17 *MS/MS spectrum of Luteoline*

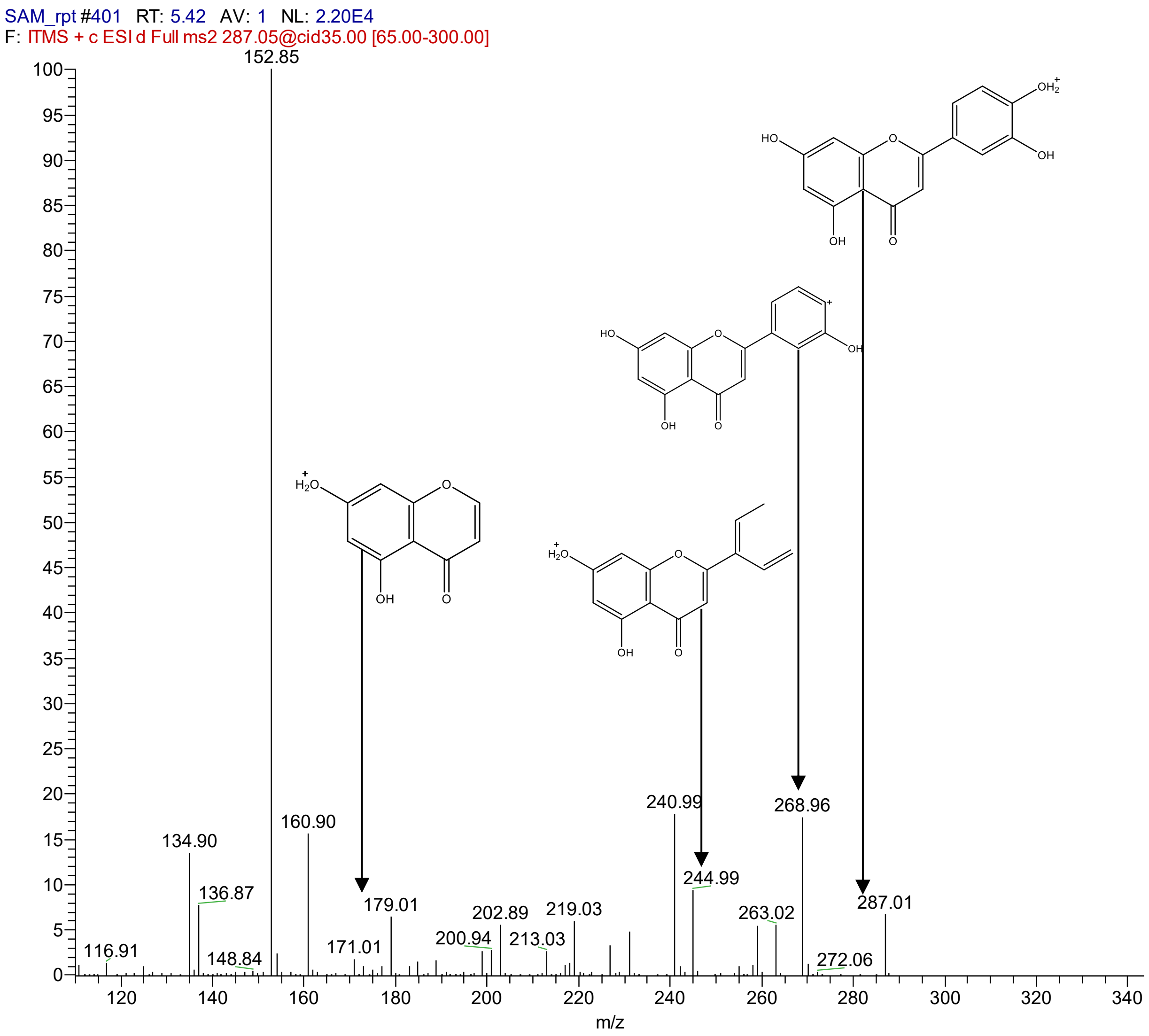


***Assigned MS and MS/MS fragments ion of Luteoline***

Molecular ion at *m/z*= 287.06 [M+H]$^+$ .was The loss of an OH group gave fragment ion *m/z* 268.96 [M+H]$^+$. Loss of an ethanediol ($C_2H_2O_2$) group from B ring resulted in fragment ion at *m/z* 244.99 [M+H]$^+$. Loss of dihydroxyphenyl B ring resulted in fragment ion at *m/z*=179.03[M+H]$^+$ (Tahir et al., 2012)**.**

### 1.1.18 *MS spectrum of Ursolic acid*

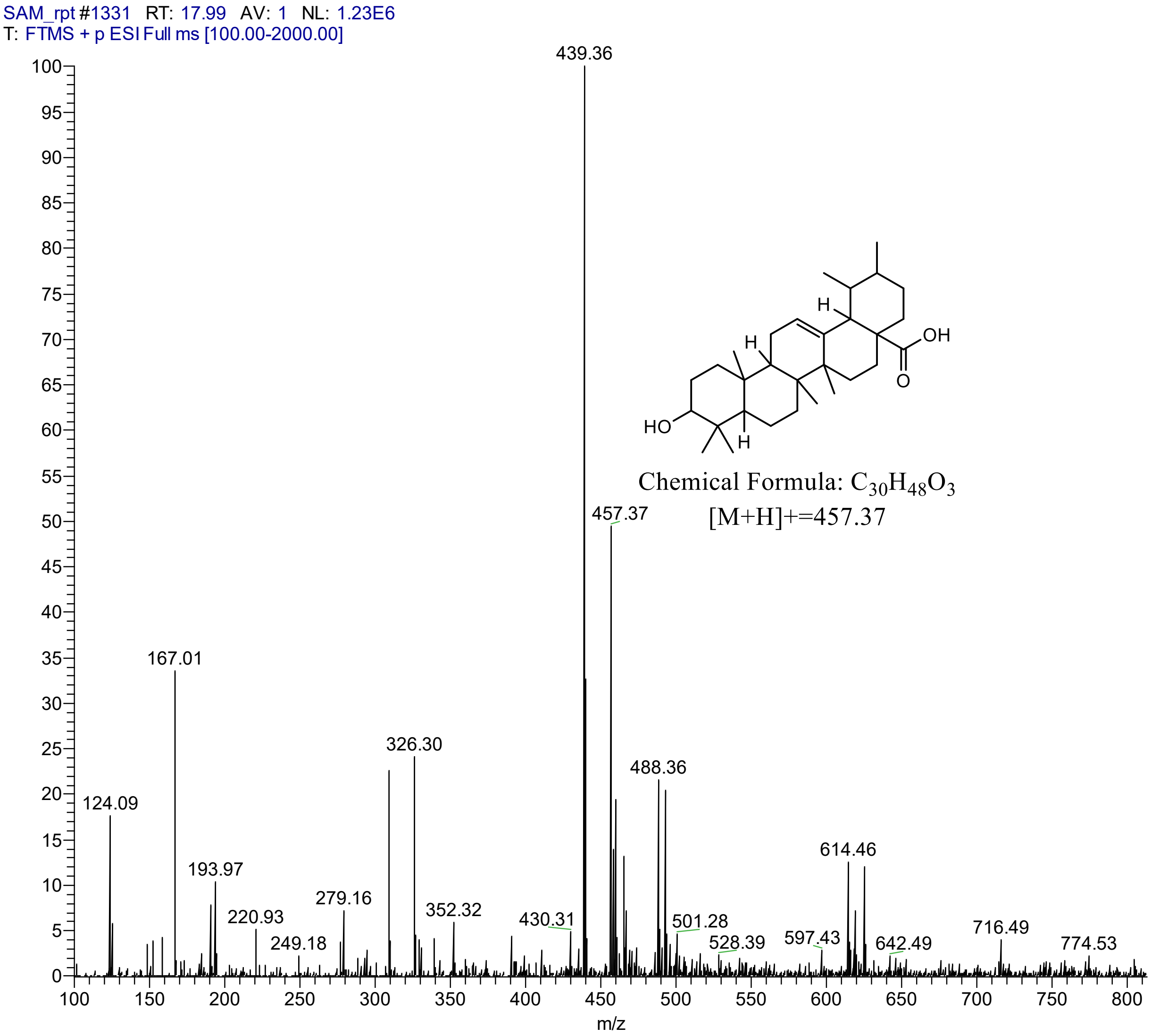

### 1.1.19 *MS/MS spectrum of Ursolic acid*

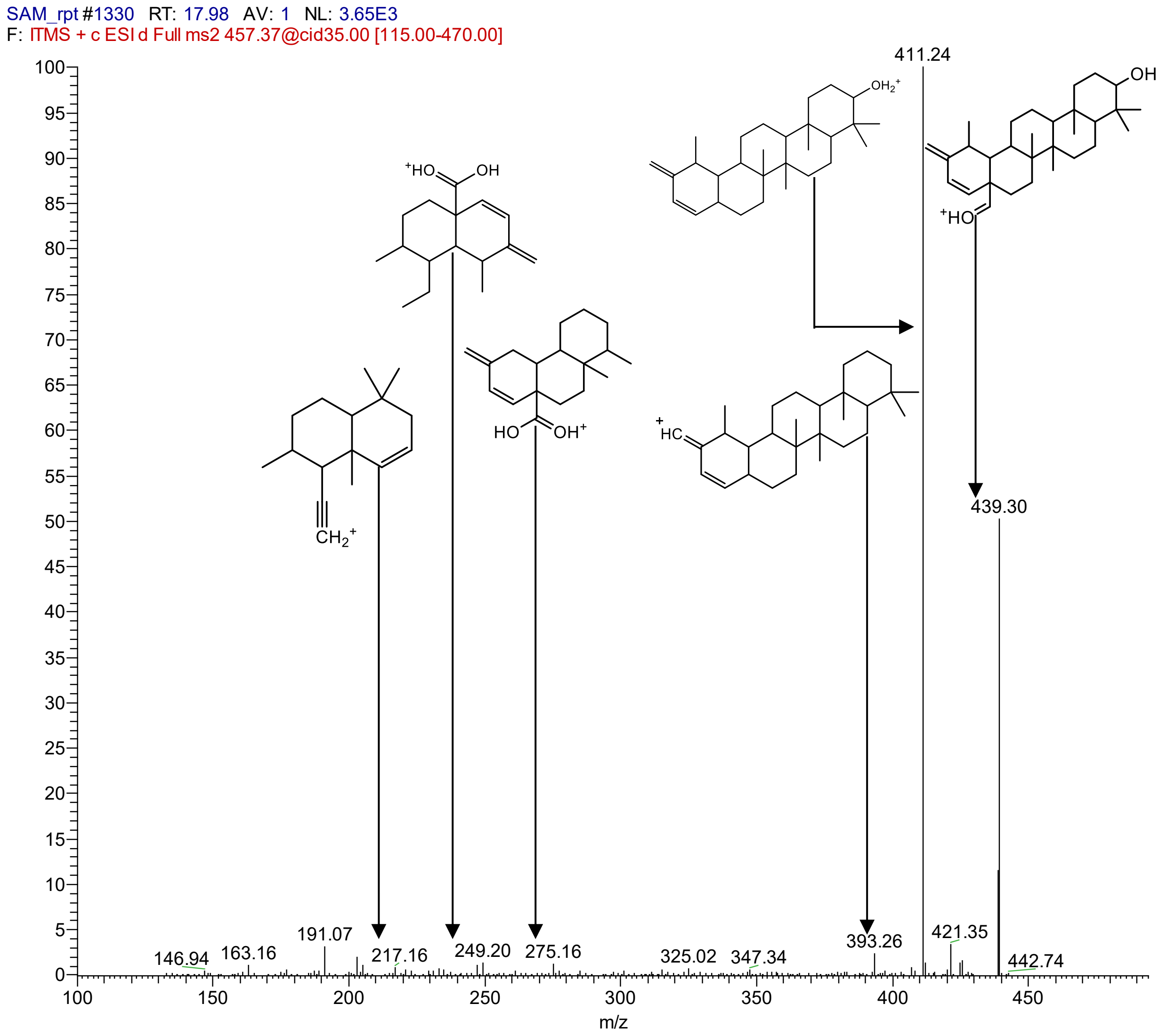


***Assigned MS and MS/MS fragments ion Ursolic acid***

Molecular ion at *m/z*= 457.37 [M+H]$^+$ .The loss of an OH group gave fragment ion *m/z* 439.30 [M+H]$^+$. Loss of an COOH resulted in fragment ion at *m/z* 411.24 [M+H]$^+$, further loss of an OH group resulted in fragment ion *m/z* 393.26 [M+H]$^+$. Loss of ring A and, B resulted in fragment ion at *m/z*= 275.16[M+H]$^+$, further degradation of ring C resulted in fragment ion at *m/z*= 249.20[M+H]$^+$. The residual fragments of ring A and B resulted in fragment ion at *m/z*=217.16[M+H]$^+$ (Razboršek et al., 2008, Novotny et al., 2003).

### 1.1.20 *MS spectrum of Stigmasterol*

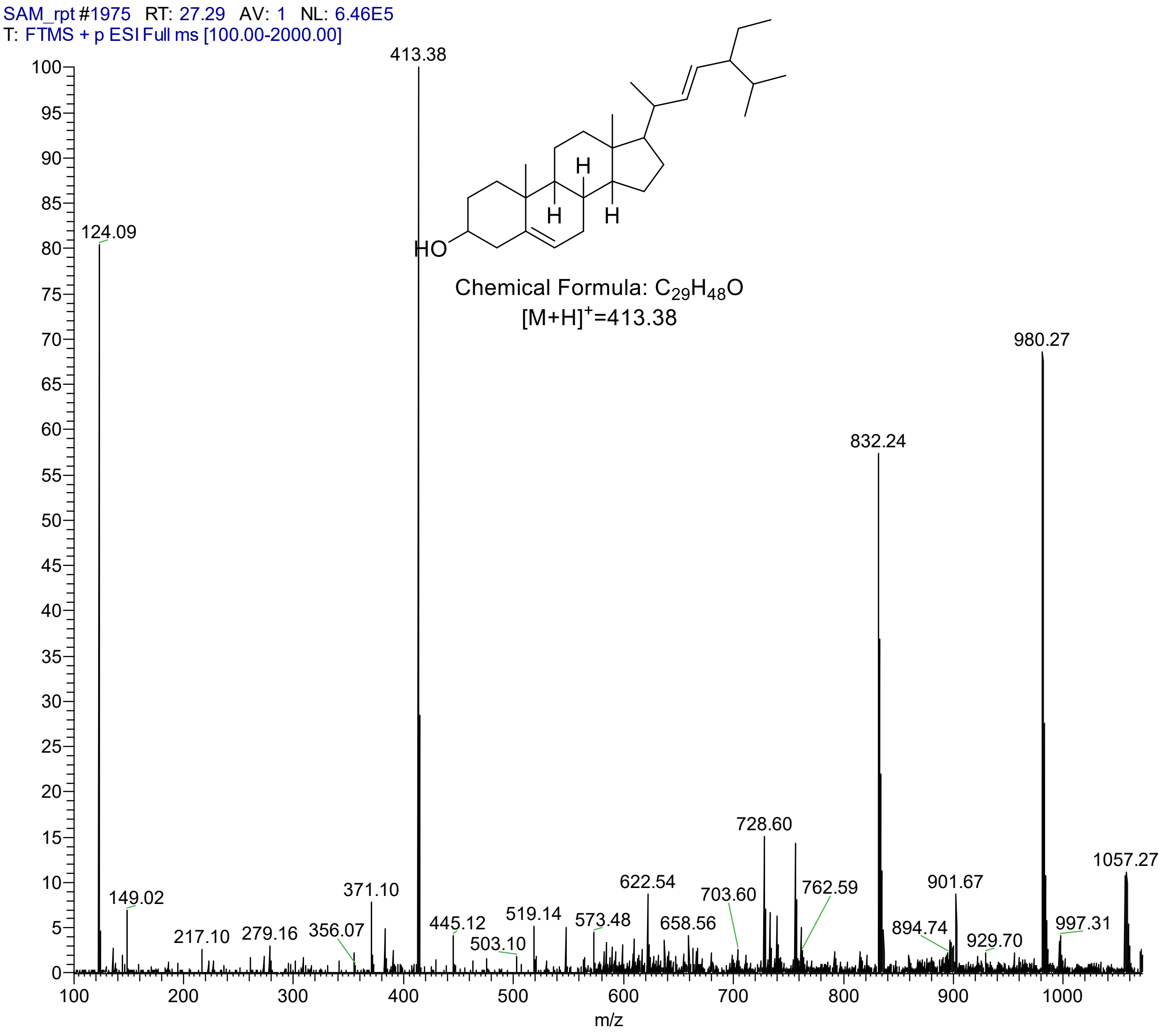

### 1.1.21 *MS/MS spectrum of Stigmasterol*

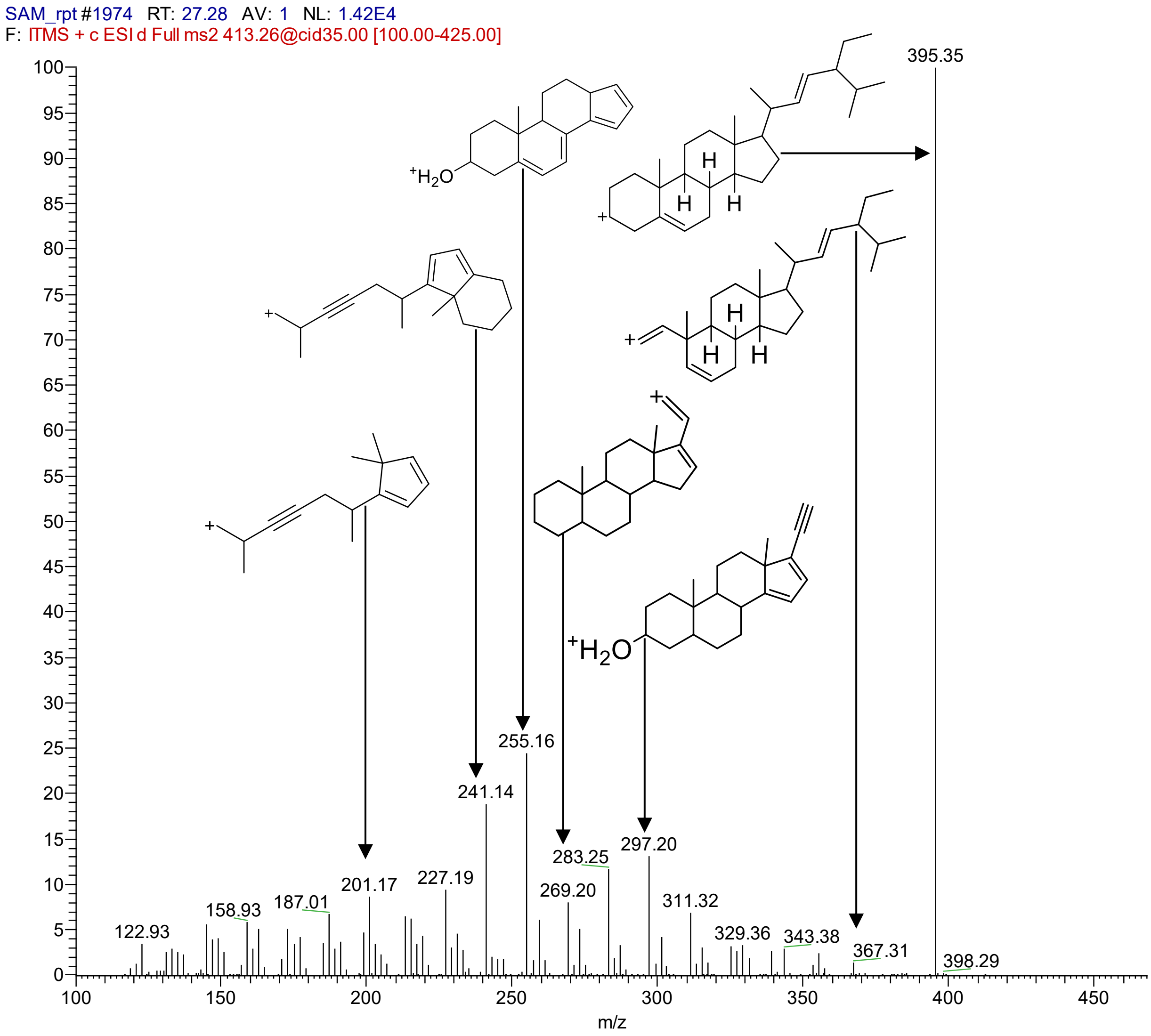


***Assigned MS and MS/MS fragments ions of Stigmasterol***

Molecular ion at *m/z*= 413.38 [M+H]$^+$ .The loss of an OH group gave fragment ion *m/z* 395.35 [M+H]$^+$. Loss of an ethanol $C_2H_4OH$ moiety due to degradation of ring A resulted in fragment ion *m/z* 367.31 [M+H]$^+$. Loss a methyl-pentene group from alkyl chain resulted in fragment ion at *m/z*= 297.20 [M+H]$^+$, which further gave fragment ion at *m/z*= 283.25 [M+H]$^+$ due to loss of an OH group. Total loss of alkyl chain resulted in fragment ion at *m/z*= 255.16 [M+H]$^+$. Simultaneous loss of ring A and B resulted in fragment ion at *m/z*=241.14, further loss of ring C resulted in fragment ion at *m/z*= 201.17 [M+H]$^+$ respectively (Jiang et al., 2019, Rozenberg et al., 2003).

## 2 HPLC method development for simultaneous detection of Gallic acid and ellagic acid in *S.apetala* fruit extract (SAM)

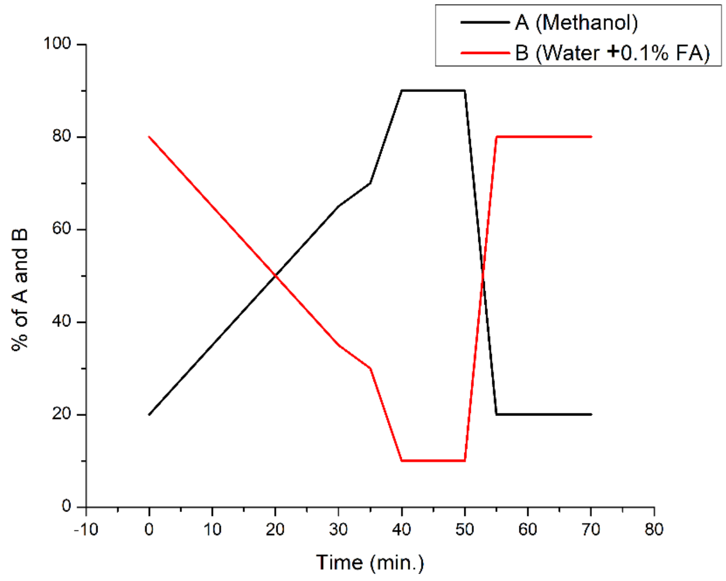


### 2.1 Linearity and sensitivity (Gallic acid)

HPLC chromatograms of Gallic acid in concentration ranging from 50 µg/ml, 100 µg/ml, 250 µg/ml, 500 µg/ml, 750 µg/ml to 1000 µg/ml. (n=3)

**Figure S1:** HPLC chromatogram of GA (50 µg/ml)

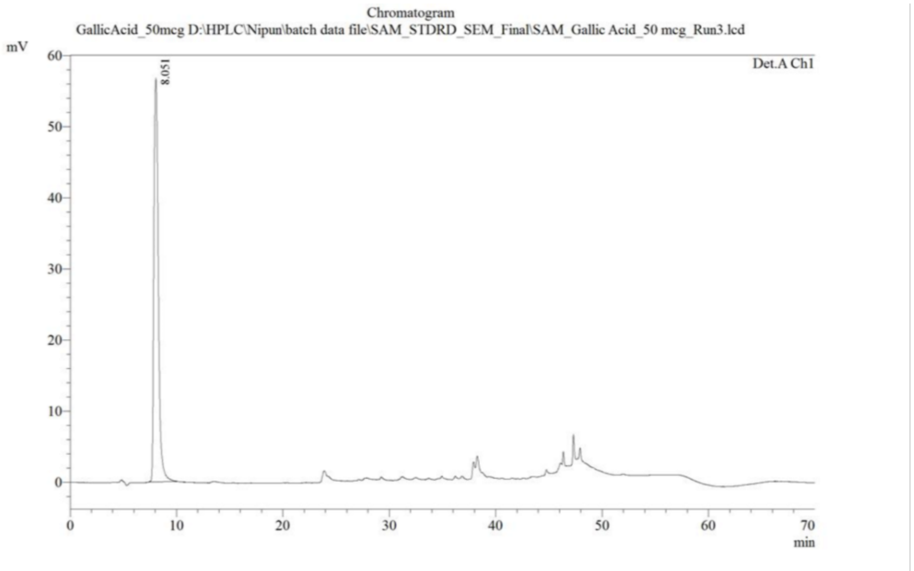


PeakTable D:\HPLC\Nipun\batch data file\SAM_STDRD_SEM_Final\SAM_Gallic Acid_50 mcg_Run3.lcd

Detector A Ch1 260nm

| Peak# | Ret. Time | Area | Height | Area % | Height % |
|---|---|---|---|---|---|
| 1 | 8.051 | 1654914 | 56774 | 100.000 | 100.000 |
| Total | | 1654914 | 56774 | 100.000 | 100.000 |

**Figure S2:** HPLC chromatogram of GA (100 µg/ml)

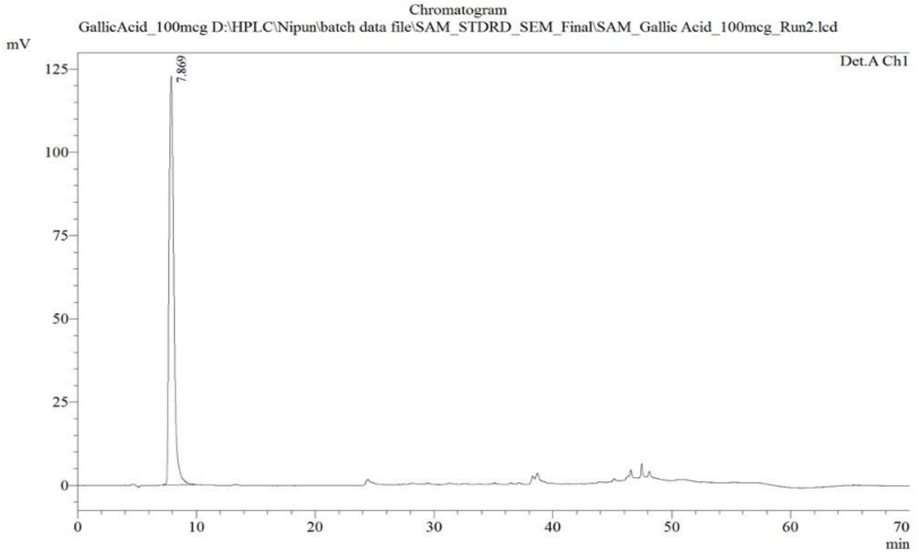


PeakTable D:\HPLC\Nipun\batch data file\SAM_STDRD_SEM_Final\SAM_Gallic Acid_100mcg_Run2.lcd

Detector A Ch1 260nm

| Peak# | Ret. Time | Area | Height | Area % | Height % |
|---|---|---|---|---|---|
| 1 | 7.869 | 3443181 | 122831 | 100.000 | 100.000 |
| Total | | 3443181 | 122831 | 100.000 | 100.000 |

**Figure S3:** HPLC chromatogram of GA (250 µg/ml)

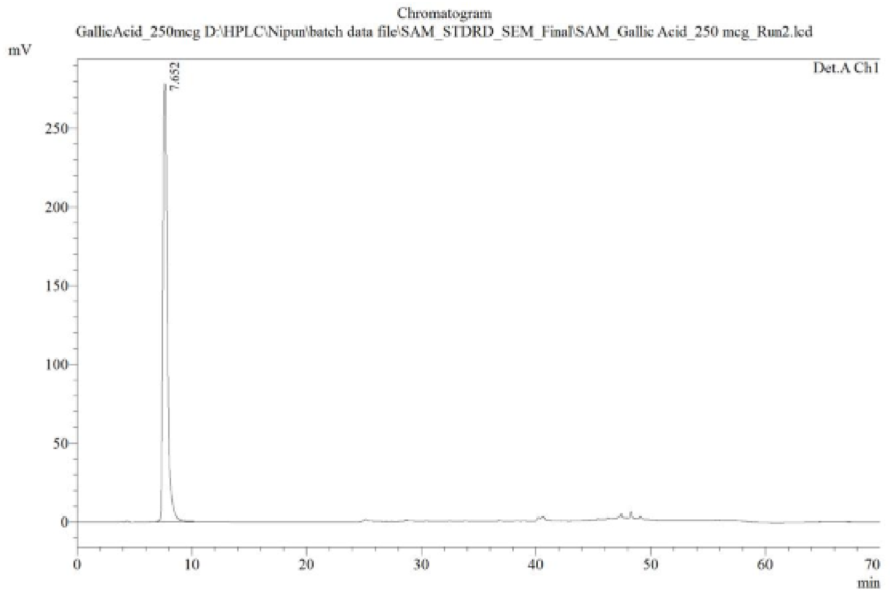


PeakTable D:\HPLC\Nipun\batch data file\SAM_STDRD_SEM_Final\SAM_Gallic Acid_250 mcg_Run2.lcd
Detector A Ch1 260nm

| Peak# | Ret. Time | Area | Height | Area % | Height % |
|---|---|---|---|---|---|
| 1 | 7.652 | 7559733 | 278284 | 100.000 | 100.000 |
| Total | | 7559733 | 278284 | 100.000 | 100.000 |

**Figure S4:** HPLC chromatogram of GA (500 µg/ml)

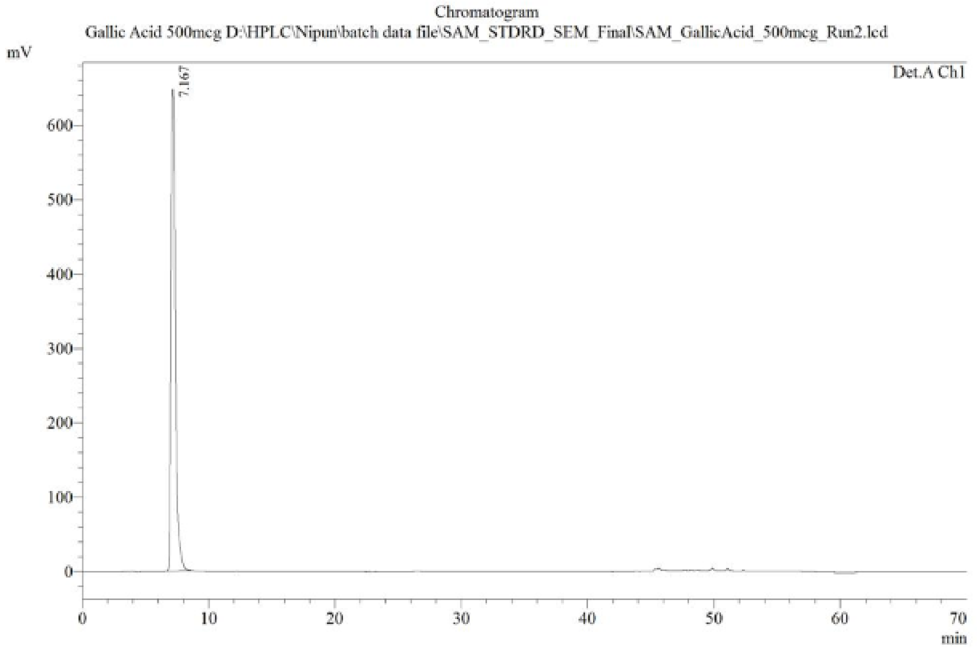


PeakTable D:\HPLC\Nipun\batch data file\SAM_STDRD_SEM_Final\SAM_GallicAcid_500mcg_Run2.lcd
Detector A Ch1 260nm

| Peak# | Ret. Time | Area | Height | Area % | Height % |
|---|---|---|---|---|---|
| 1 | 7.167 | 16003105 | 647669 | 100.000 | 100.000 |
| Total | | 16003105 | 647669 | 100.000 | 100.000 |

**Figure S5:** HPLC chromatogram of GA (750 µg/ml)

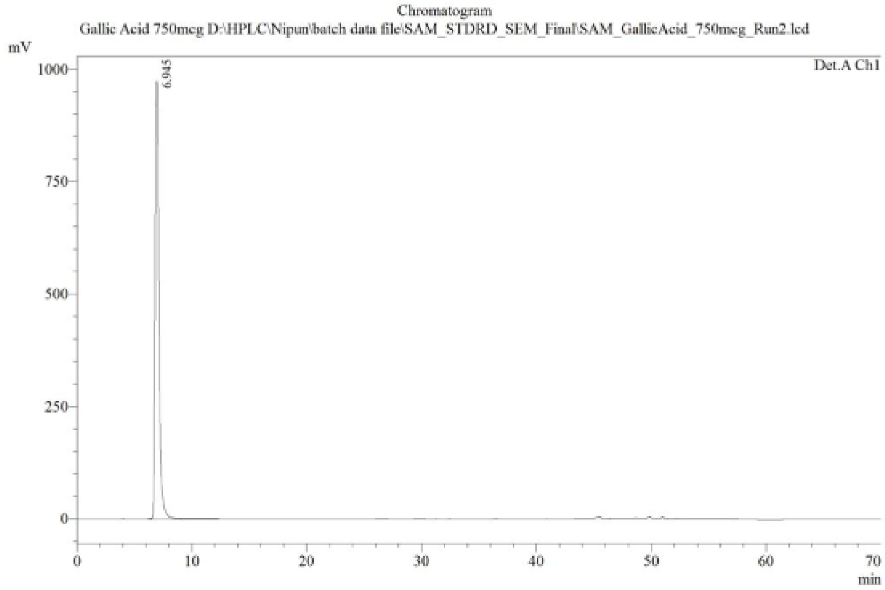


PeakTable D:\HPLC\Nipun\batch data file\SAM_STDRD_SEM_Final\SAM_GallicAcid_750mcg_Run2.lcd
Detector A Ch1 260nm

| Peak# | Ret. Time | Area | Height | Area % | Height % |
|---|---|---|---|---|---|
| 1 | 6.945 | 22934236 | 974611 | 100.000 | 100.000 |
| Total | | 22934236 | 974611 | 100.000 | 100.000 |

**Figure S6:** HPLC chromatogram of GA (1000 µg/ml)

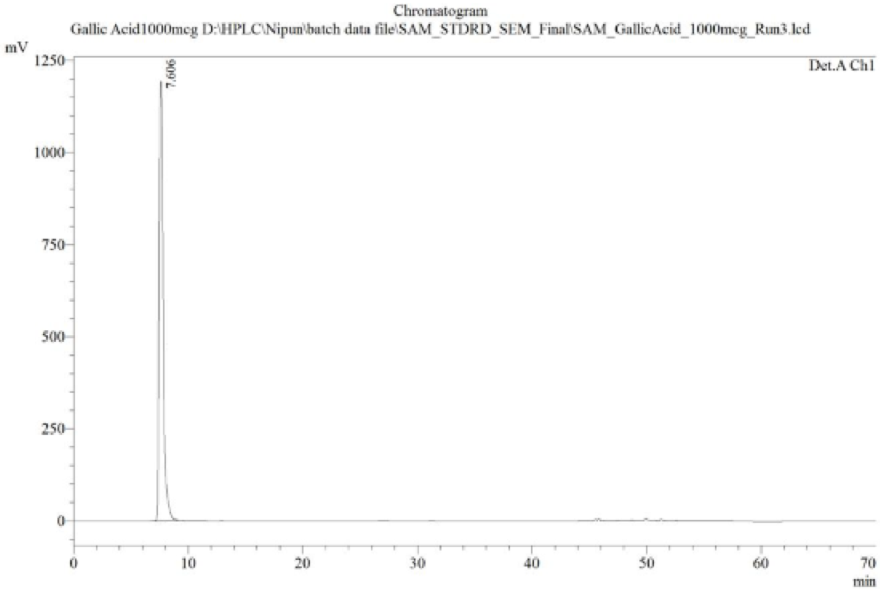


PeakTable D:\HPLC\Nipun\batch data file\SAM_STDRD_SEM_Final\SAM_GallicAcid_1000mcg_Run3.lcd
Detector A Ch1 260nm

| Peak# | Ret. Time | Area | Height | Area % | Height % |
|---|---|---|---|---|---|
| 1 | 7.606 | 29674647 | 1192720 | 100.000 | 100.000 |
| Total | | 29674647 | 1192720 | 100.000 | 100.000 |

## 2.2 Linearity and sensitivity (Ellagic acid)

**Figure S7:** HPLC chromatogram of EA (50 µg/ml)

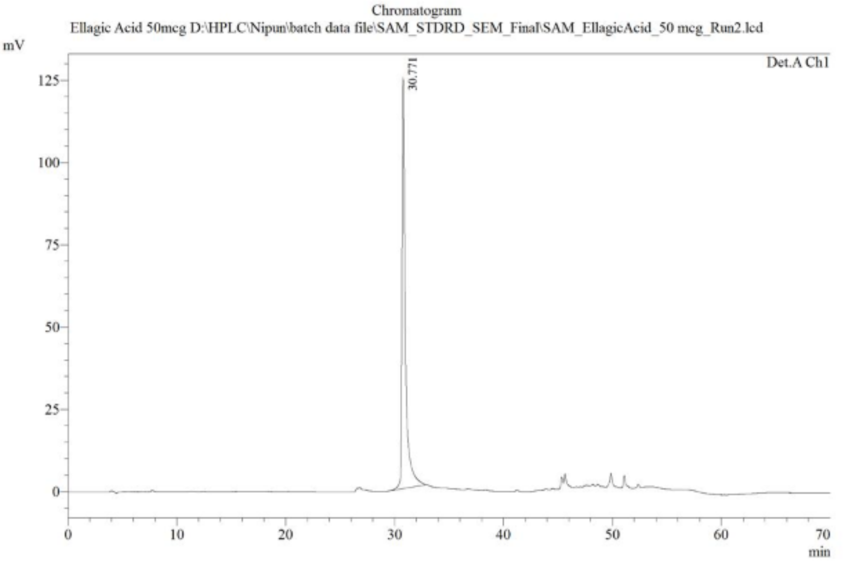


PeakTable D:\HPLC\Nipun\batch data file\SAM_STDRD_SEM_Final\SAM_EllagicAcid_50 mcg_Run2.lcd
Detector A Ch1 260nm

| Peak# | Ret. Time | Area | Height | Area % | Height % |
|---|---|---|---|---|---|
| 1 | 30.771 | 2712005 | 124991 | 100.000 | 100.000 |
| Total | | 2712005 | 124991 | 100.000 | 100.000 |

**Figure S8:** HPLC chromatogram of EA (100 µg/ml)

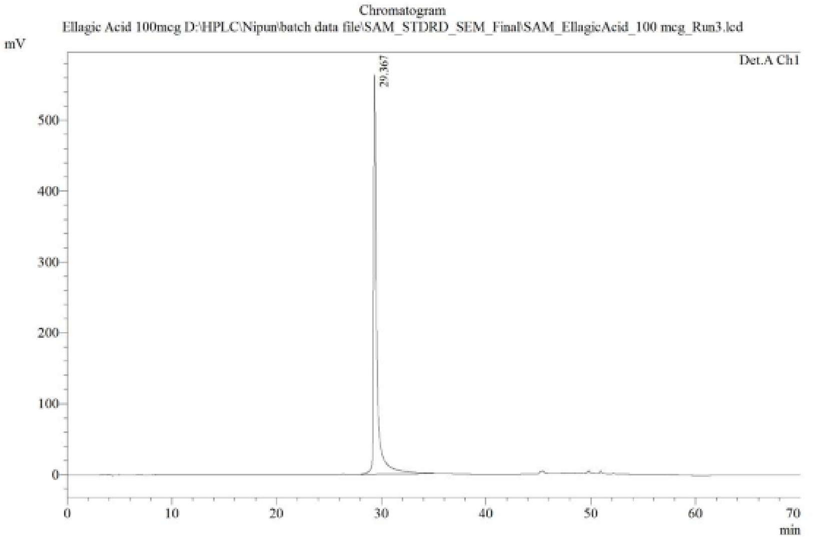


PeakTable D:\HPLC\Nipun\batch data file\SAM_STDRD_SEM_Final\SAM_EllagicAcid_100 mcg_Run3.lcd
Detector A Ch1 260nm

| Peak# | Ret. Time | Area | Height | Area % | Height % |
|---|---|---|---|---|---|
| 1 | 29.367 | 12297027 | 563934 | 100.000 | 100.000 |
| Total | | 12297027 | 563934 | 100.000 | 100.000 |

**Figure S9:** HPLC chromatogram of EA (250 µg/ml)

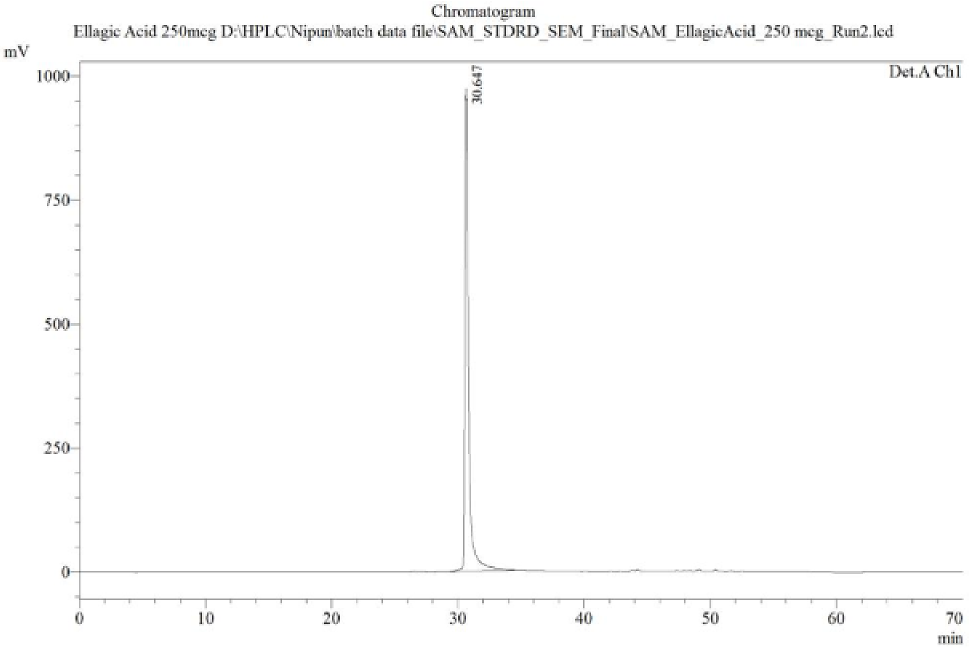


PeakTable D:\HPLC\Nipun\batch data file\SAM_STDRD_SEM_Final\SAM_EllagicAcid_250 mcg_Run2.lcd
Detector A Ch1 260nm

| Peak# | Ret. Time | Area | Height | Area % | Height % |
|---|---|---|---|---|---|
| 1 | 30.647 | 19647227 | 973303 | 100.000 | 100.000 |
| Total | | 19647227 | 973303 | 100.000 | 100.000 |

**Figure S10:** HPLC chromatogram of EA (500 µg/ml)

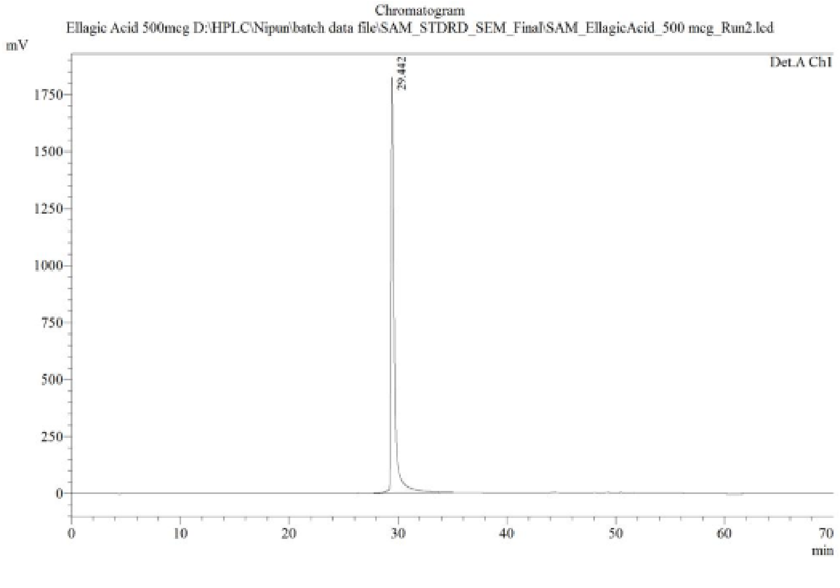


PeakTable D:\HPLC\Nipun\batch data file\SAM_STDRD_SEM_Final\SAM_EllagicAcid_500 mcg_Run2.lcd
Detector A Ch1 260nm

| Peak# | Ret. Time | Area | Height | Area % | Height % |
|---|---|---|---|---|---|
| 1 | 29.442 | 40529195 | 1825171 | 100.000 | 100.000 |
| Total | | 40529195 | 1825171 | 100.000 | 100.000 |

**Figure S11:** HPLC chromatogram of EA (750 µg/ml)

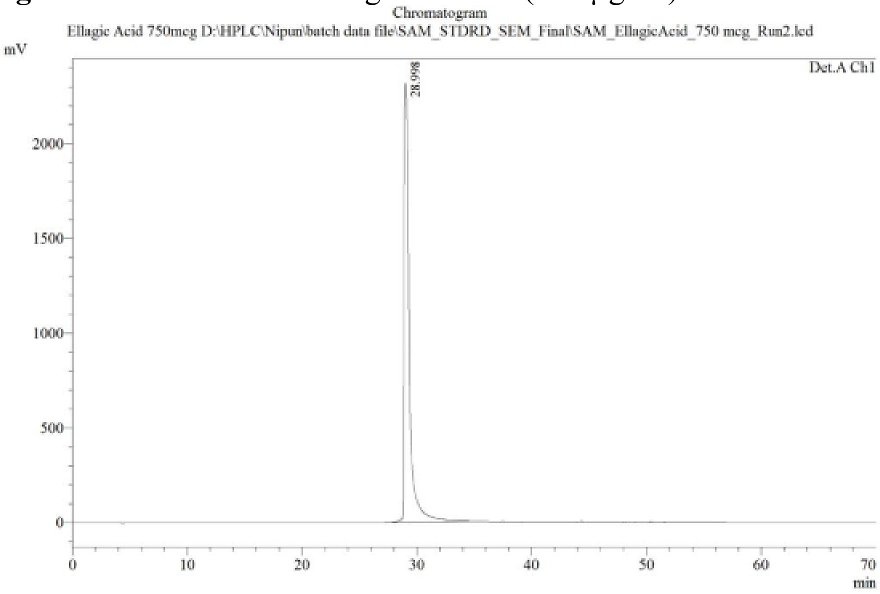


PeakTable D:\HPLC\Nipun\batch data file\SAM_STDRD_SEM_Final\SAM_EllagicAcid_750 mcg_Run2.lcd

Detector A Ch1 260nm

| Peak# | Ret. Time | Area | Height | Area % | Height % |
|---|---|---|---|---|---|
| 1 | 28.998 | 75799922 | 2316745 | 100.000 | 100.000 |
| Total | | 75799922 | 2316745 | 100.000 | 100.000 |

**Figure S12:** HPLC chromatogram of EA (1000 µg/ml)

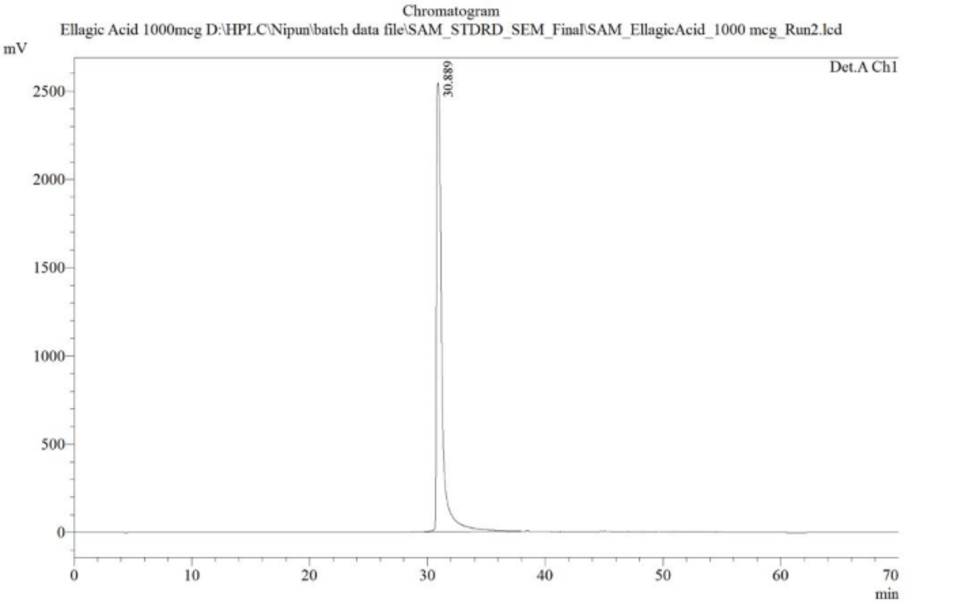


PeakTable D:\HPLC\Nipun\batch data file\SAM_STDRD_SEM_Final\SAM_EllagicAcid_1000 mcg_Run2.lcd

Detector A Ch1 260nm

| Peak# | Ret. Time | Area | Height | Area % | Height % |
|---|---|---|---|---|---|
| 1 | 30.889 | 82109656 | 2542801 | 100.000 | 100.000 |
| Total | | 82109656 | 2542801 | 100.000 | 100.000 |

### 2.3 Accuracy (Gallic acid)

SAM extract 5 mg/ml (20µl) was spiked with different concentration of Gallic acid 125 µg/ml, 500 µg/ml and 1000µg/ml (injection volume equivalent to 20µl) (n=3).

**Figure S13:** SAM+ 125 µg/ml GA

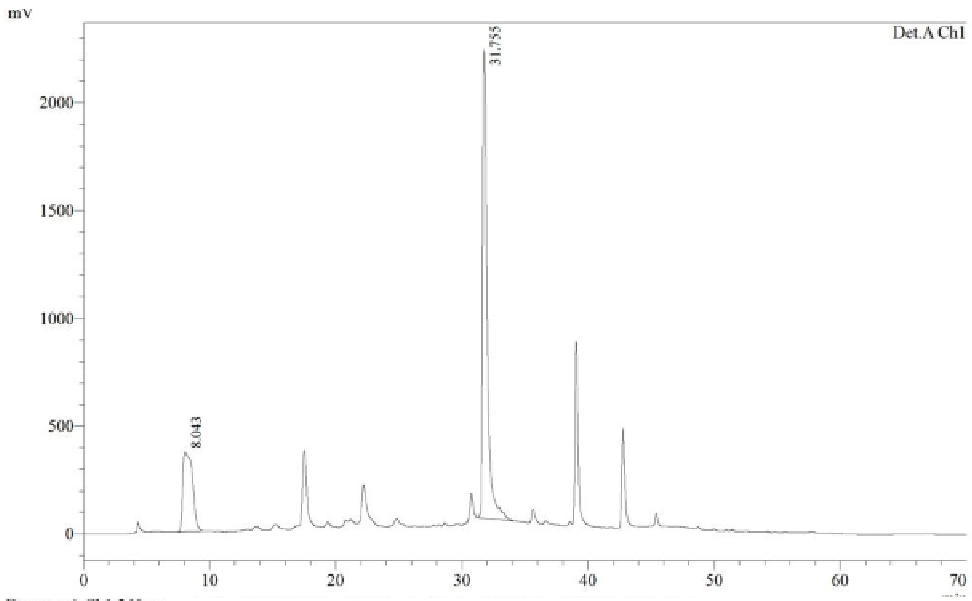


Detector A Ch1 260nm

| Peak# | Ret. Time | Area | Height | Area % | Height % |
|---|---|---|---|---|---|
| 1 | 8.043 | 19841222 | 369748 | 25.840 | 14.547 |
| 2 | 31.755 | 56943317 | 2172027 | 74.160 | 85.453 |
| Total | | 76784540 | 2541775 | 100.000 | 100.000 |

**Figure S13:** SAM+ 500 µg/ml GA

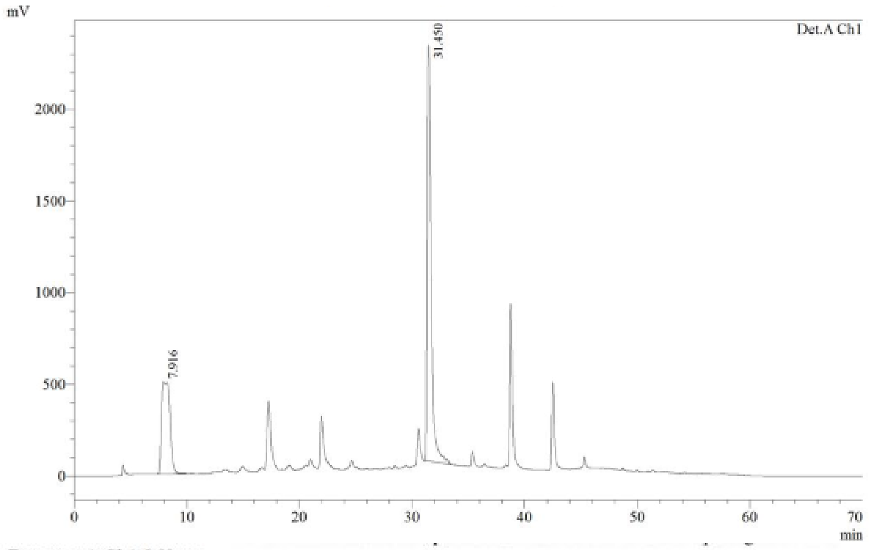


Detector A Ch1 260nm

| Peak# | Ret. Time | Area | Height | Area % | Height % |
|---|---|---|---|---|---|
| 1 | 7.916 | 26387439 | 501986 | 31.212 | 18.100 |
| 2 | 31.450 | 58156443 | 2271424 | 68.788 | 81.900 |
| Total | | 84543883 | 2773410 | 100.000 | 100.000 |

**Figure S14:** SAM+ 1000 µg/ml GA

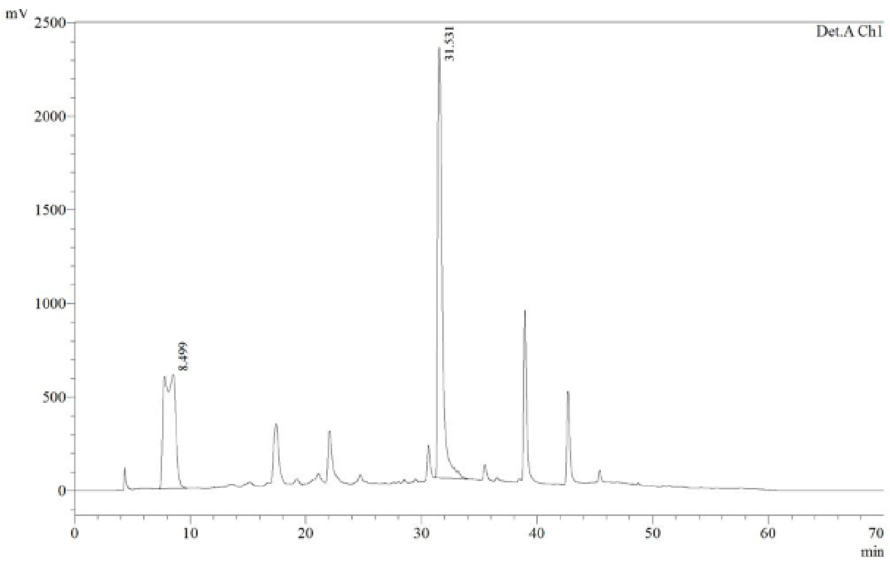


Detector A Ch1 260nm

| Peak# | Ret. Time | Area | Height | Area % | Height % |
|---|---|---|---|---|---|
| 1 | 8.499 | 42631179 | 608812 | 40.269 | 20.929 |
| 2 | 31.531 | 63235846 | 2300103 | 59.731 | 79.071 |
| Total | | 105867025 | 2908916 | 100.000 | 100.000 |

## 2.4 Accuracy (Ellagic acid)

SAM extract 5 mg/ml (20µl) was spiked with different concentration of Ellagic acid (EA) 125 µg/ml, 250 µg/ml and 500 µg/ml (injection volume equivalent to 20µl) (n=3).

**Figure S15:** SAM+ 125 µg/ml EA

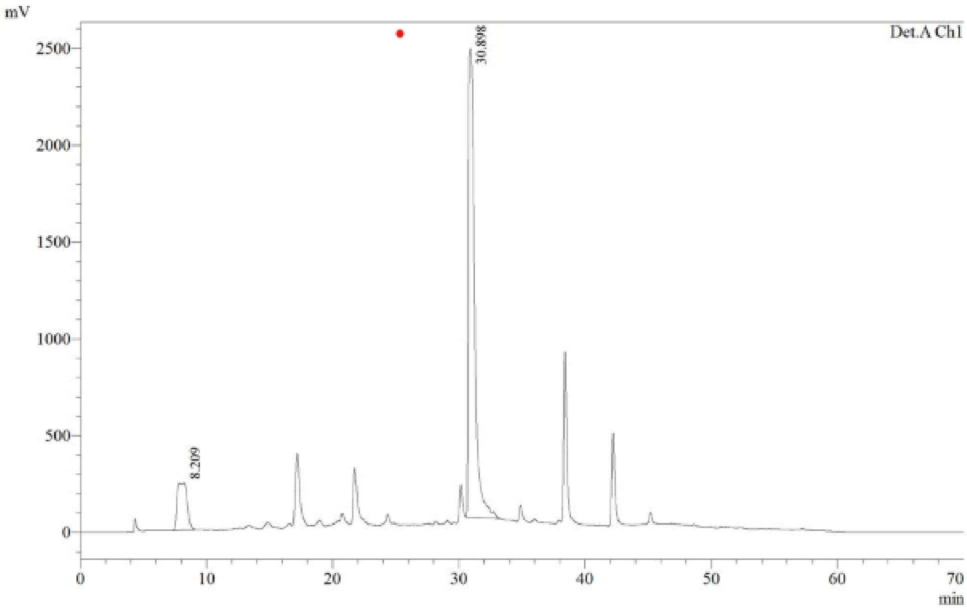


Detector A Ch1 260nm

| Peak# | Ret. Time | Area | Height | Area % | Height % |
|---|---|---|---|---|---|
| 1 | 7.999 | 14001883 | 280026 | 15.437 | 10.318 |
| 2 | 31.458 | 76704353 | 2433905 | 84.563 | 89.682 |
| Total | | 90706236 | 2713931 | 100.000 | 100.000 |

**Figure S16:** SAM+ 250 µg/ml EA

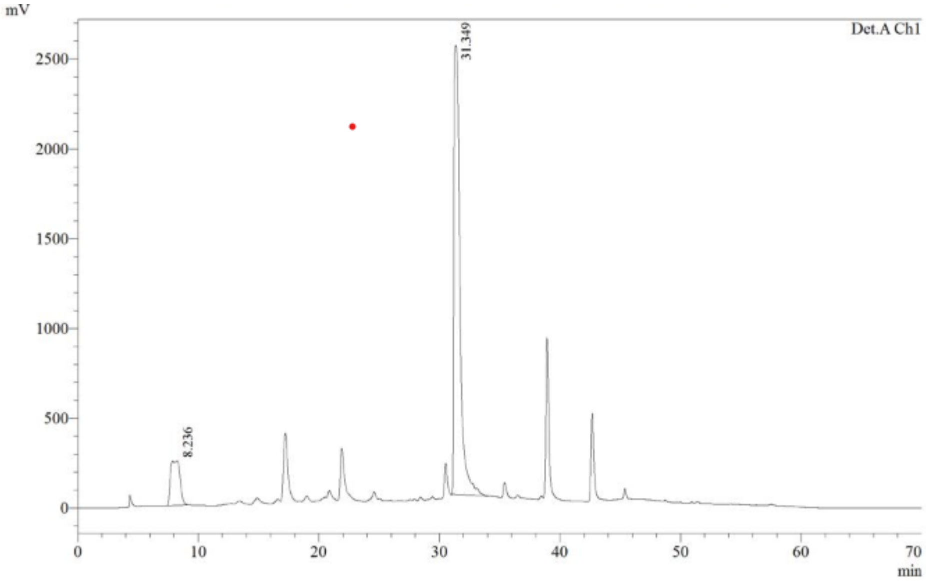


Detector A Ch1 260nm

| Peak# | Ret. Time | Area | Height | Area % | Height % |
|---|---|---|---|---|---|
| 1 | 8.236 | 13484065 | 247549 | 13.281 | 8.999 |
| 2 | 31.349 | 88048063 | 2503448 | 86.719 | 91.001 |
| Total | | 101532129 | 2750996 | 100.000 | 100.000 |

**Figure S17:** SAM+ 500 µg/ml EA

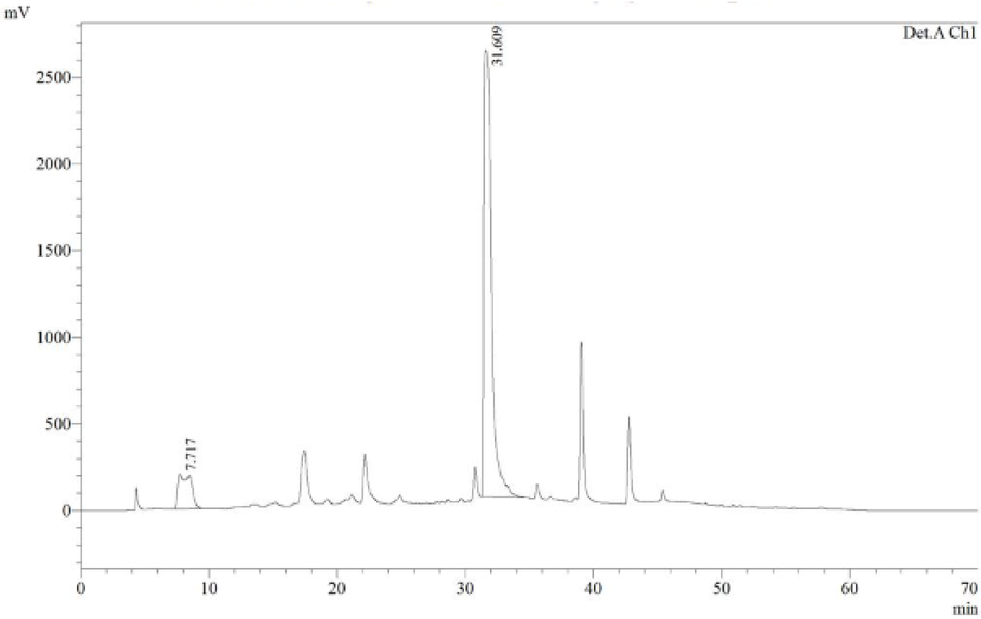


Detector A Ch1 260nm

| Peak# | Ret. Time | Area | Height | Area % | Height % |
|---|---|---|---|---|---|
| 1 | 7.717 | 14011571 | 197625 | 11.528 | 7.120 |
| 2 | 31.609 | 107535390 | 2577942 | 88.472 | 92.880 |
| Total | | 121546960 | 2775567 | 100.000 | 100.000 |

## 2.5 Intra-day and Inter-day Precision (Gallic acid)

Peak area of Gallic acid (500 µg/ml) the morning and evening (n=3) three consecutive days.

**Figure S18:** Gallic acid (500 µg/ml) Day 1, Morning

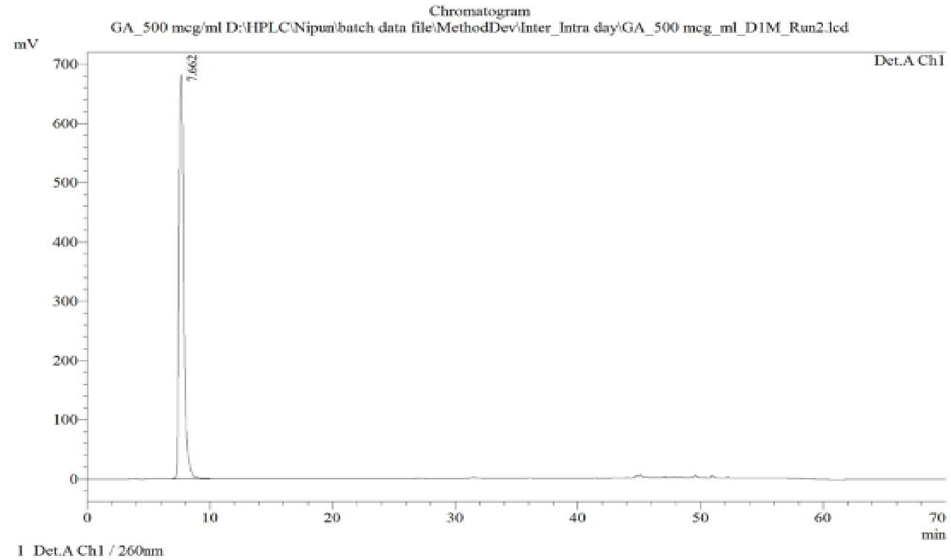


PeakTable D:\HPLC\Nipun\batch data file\MethodDev\Inter_Intra day\GA_500 mcg_ml_D1M_Run2.lcd
Detector A Ch1 260nm

| Peak# | Ret. Time | Area | Height | Area % | Height % |
|---|---|---|---|---|---|
| 1 | 7.662 | 19436159 | 681214 | 100.000 | 100.000 |
| Total | | 19436159 | 681214 | 100.000 | 100.000 |

**Figure S19:** Gallic acid (500 µg/ml) Day 1, Evening

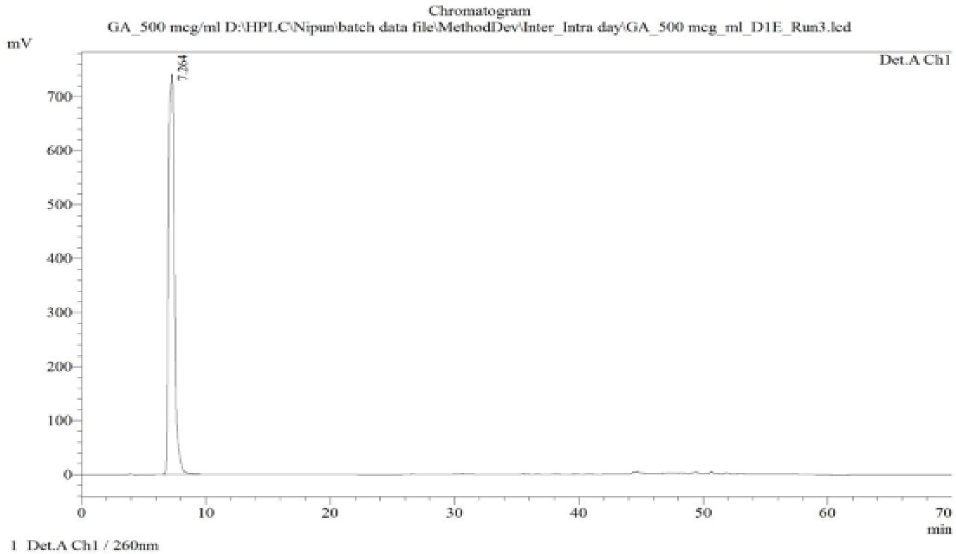


PeakTable D:\HPLC\Nipun\batch data file\MethodDev\Inter_Intra day\GA_500 mcg_ml_D1E_Run3.lcd
Detector A Ch1 260nm

| Peak# | Ret. Time | Area | Height | Area % | Height % |
|---|---|---|---|---|---|
| 1 | 7.264 | 24775682 | 741170 | 100.000 | 100.000 |
| Total | | 24775682 | 741170 | 100.000 | 100.000 |

**Figure S20:** Gallic acid (500 µg/ml) Day 2, Morning

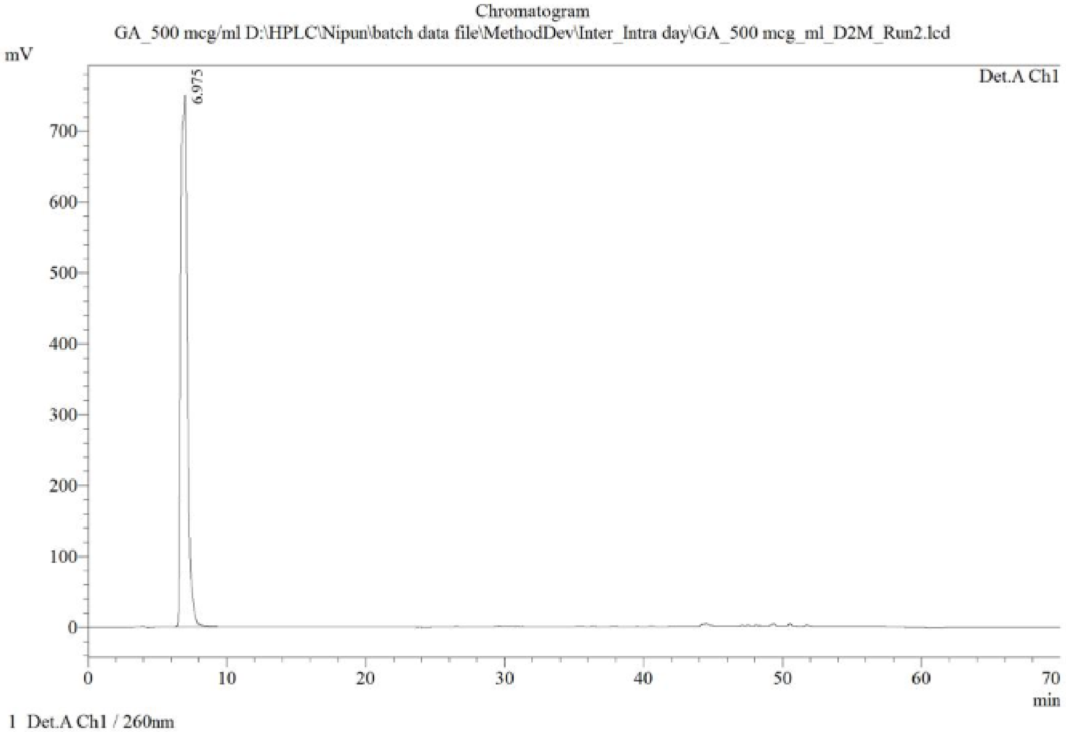


PeakTable D:\HPLC\Nipun\batch data file\MethodDev\Inter_Intra day\GA_500 mcg_ml_D2M_Run2.lcd
Detector A Ch1 260nm

| Peak# | Ret. Time | Area | Height | Area % | Height % |
|---|---|---|---|---|---|
| 1 | 6.975 | 25408066 | 750408 | 100.000 | 100.000 |
| Total | | 25408066 | 750408 | 100.000 | 100.000 |

**Figure S21:** Gallic acid (500 µg/ml) Day 2, Evening

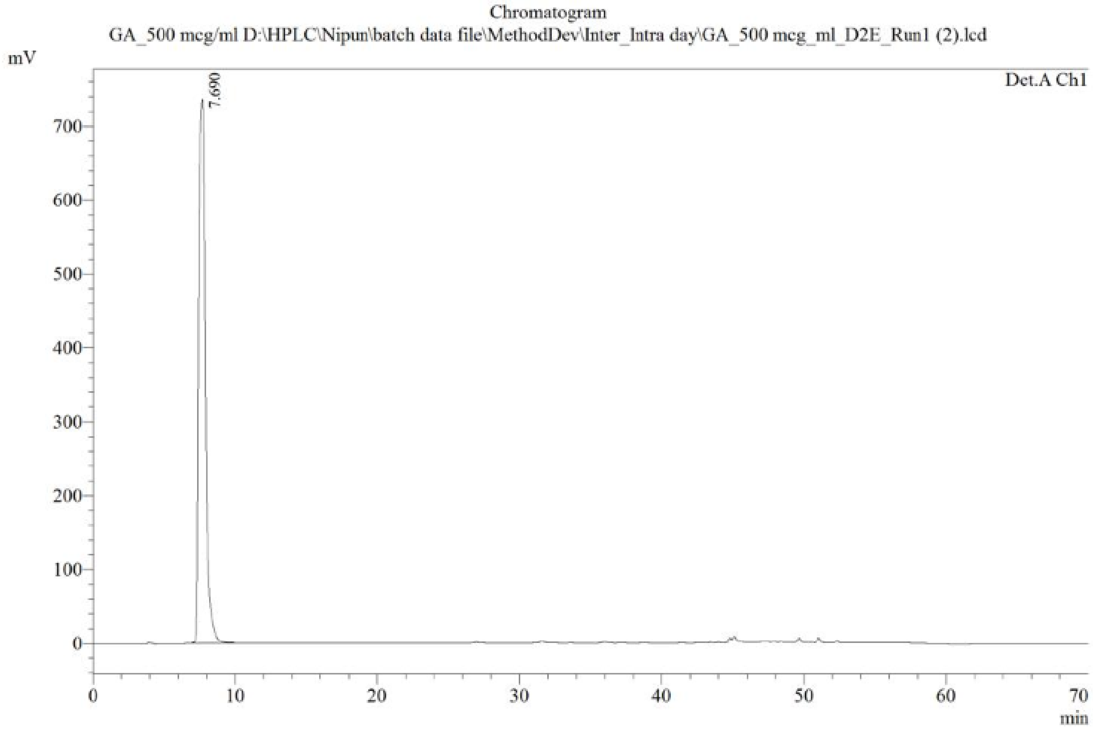


PeakTable D:\HPLC\Nipun\batch data file\MethodDev\Inter_Intra day\GA_500 mcg_ml_D2E_Run1 (2).lcd

Detector A Ch1 260nm

| Peak# | Ret. Time | Area | Height | Area % | Height % |
|---|---|---|---|---|---|
| 1 | 7.690 | 24341070 | 735801 | 100.000 | 100.000 |
| Total | | 24341070 | 735801 | 100.000 | 100.000 |

**Figure S22:** Gallic acid (500 µg/ml) Day 3, Morning

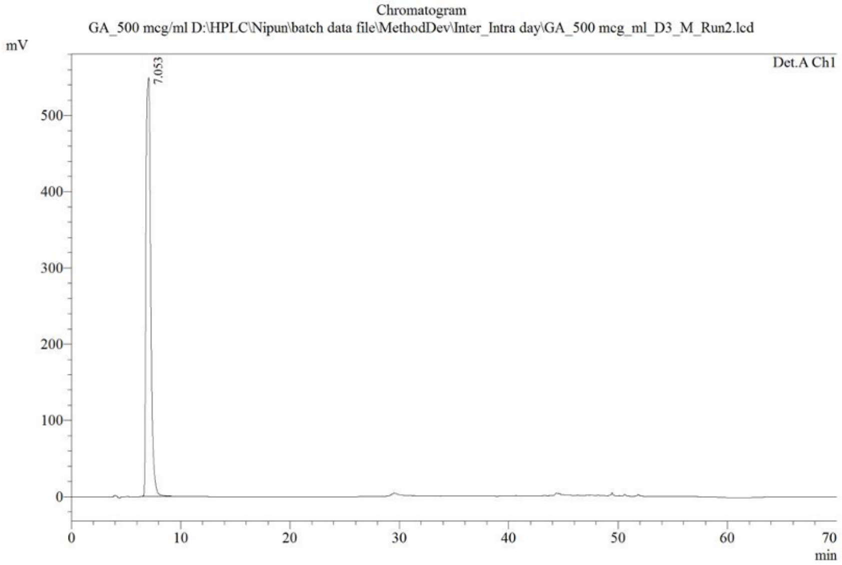


PeakTable D:\HPLC\Nipun\batch data file\MethodDev\Inter_Intra day\GA_500 mcg_ml_D3_M_Run2.lcd

Detector A Ch1 255nm

| Peak# | Ret. Time | Area | Height | Area % | Height % |
|---|---|---|---|---|---|
| 1 | 7.053 | 16513257 | 549455 | 100.000 | 100.000 |
| Total | | 16513257 | 549455 | 100.000 | 100.000 |

**Figure S23:** Gallic acid (500 µg/ml) Day 3, Evening

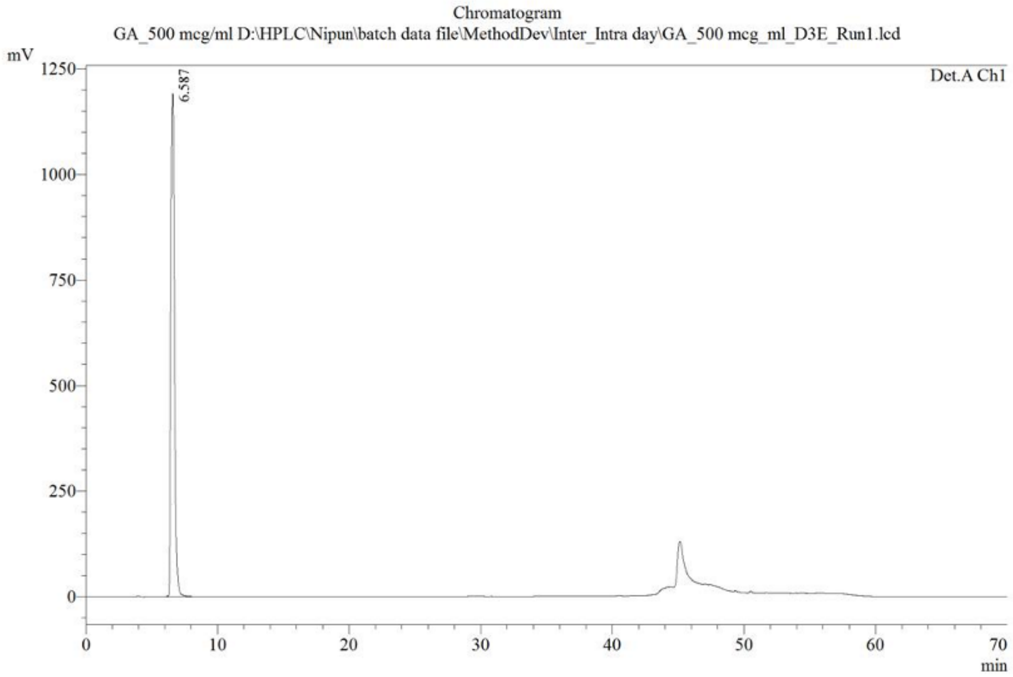


PeakTable D:\HPLC\Nipun\batch data file\MethodDev\Inter_Intra day\GA_500 mcg_ml_D3E_Run1.lcd

Detector A Ch1 260nm

| Peak# | Ret. Time | Area | Height | Area % | Height % |
|---|---|---|---|---|---|
| 1 | 6.587 | 22856990 | 1191446 | 100.000 | 100.000 |
| Total | | 22856990 | 1191446 | 100.000 | 100.000 |

## 2.6 Intra-day and Inter-day Precision (Ellagic acid)

Peak area of Ellagic acid (750 µg/ml) the morning and evening (n=3) three consecutive days.

**Figure S25:** Ellagic acid (750 µg/ml) Day 1, Morning

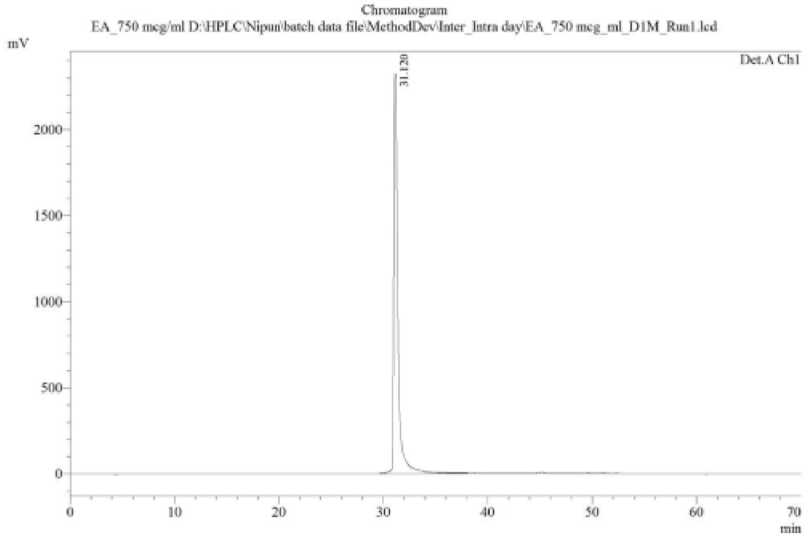


PeakTable D:\HPLC\Nipun\batch data file\MethodDev\Inter_Intra day\EA_750 mcg_ml_D1M_Run1.lcd
Detector A Ch1 260nm

| Peak# | Ret. Time | Area | Height | Area % | Height % |
|---|---|---|---|---|---|
| 1 | 31.120 | 64495726 | 2320927 | 100.000 | 100.000 |
| Total | | 64495726 | 2320927 | 100.000 | 100.000 |

**Figure S26:** Ellagic acid (750 µg/ml) Day 1, Evening

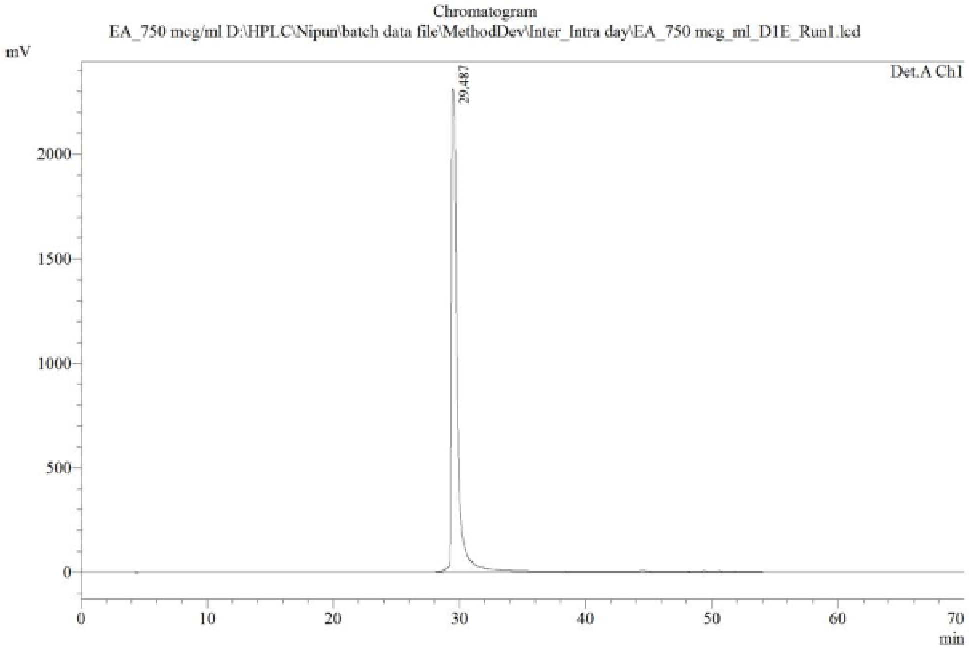


PeakTable D:\HPLC\Nipun\batch data file\MethodDev\Inter_Intra day\EA_750 mcg_ml_D1E_Run1.lcd
Detector A Ch1 260nm

| Peak# | Ret. Time | Area | Height | Area % | Height % |
|---|---|---|---|---|---|
| 1 | 29.487 | 78486258 | 2312668 | 100.000 | 100.000 |
| Total | | 78486258 | 2312668 | 100.000 | 100.000 |

**Figure S27:** Ellagic acid (750 µg/ml) Day 2, Morning

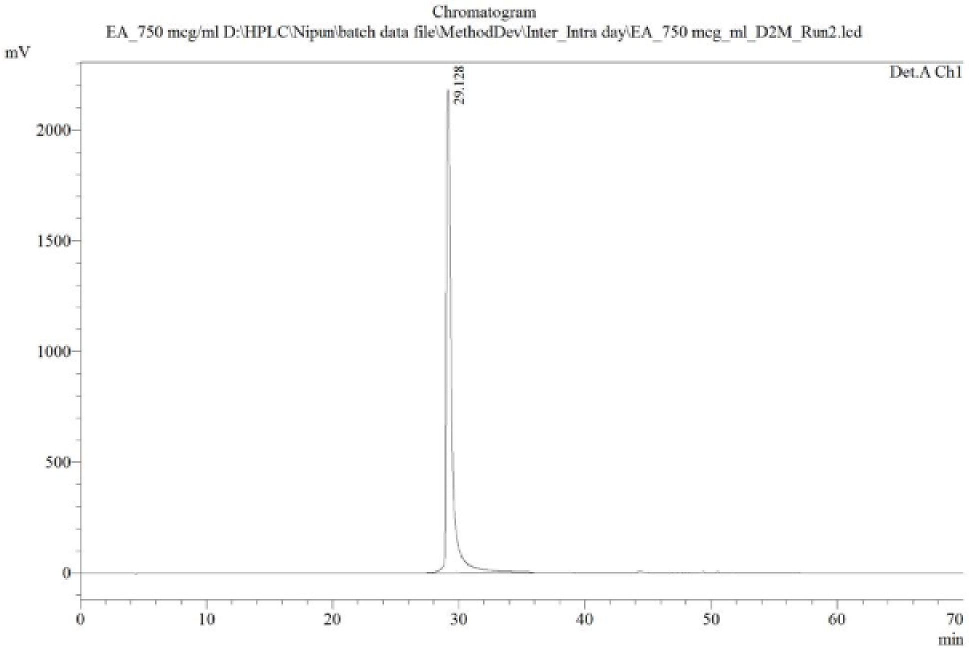


PeakTable D:\HPLC\Nipun\batch data file\MethodDev\Inter_Intra day\EA_750 mcg_ml_D2M_Run2.lcd
Detector A Ch1 260nm

| Peak# | Ret. Time | Area | Height | Area % | Height % |
|---|---|---|---|---|---|
| 1 | 29.128 | 65046760 | 2181918 | 100.000 | 100.000 |
| Total | | 65046760 | 2181918 | 100.000 | 100.000 |

**Figure S28:** Ellagic acid (750 µg/ml) Day 2, Evening

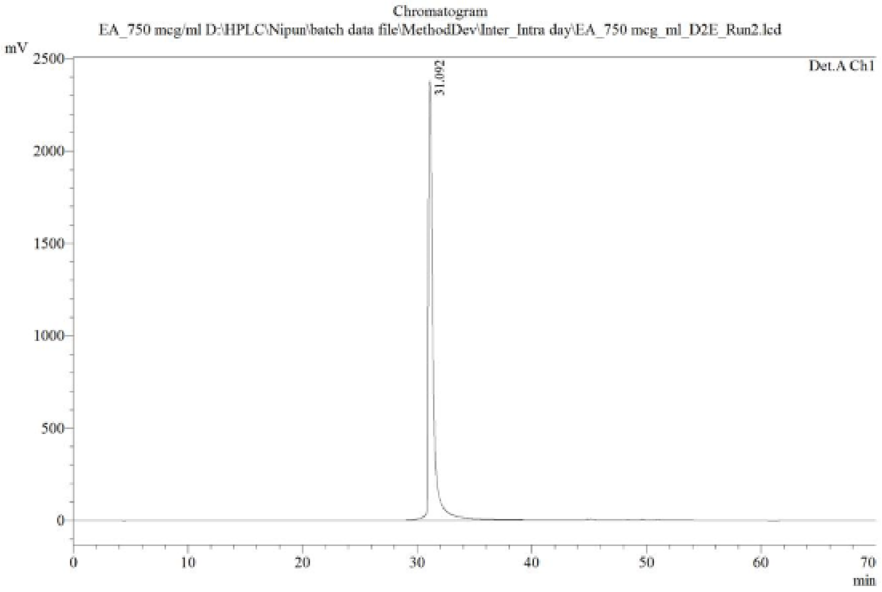


PeakTable D:\HPLC\Nipun\batch data file\MethodDev\Inter_Intra day\EA_750 mcg_ml_D2E_Run2.lcd
Detector A Ch1 260nm

| Peak# | Ret. Time | Area | Height | Area % | Height % |
|---|---|---|---|---|---|
| 1 | 31.092 | 71136669 | 2373957 | 100.000 | 100.000 |
| Total | | 71136669 | 2373957 | 100.000 | 100.000 |

**Figure S29:** Ellagic acid (750 µg/ml) Day 3, Morning

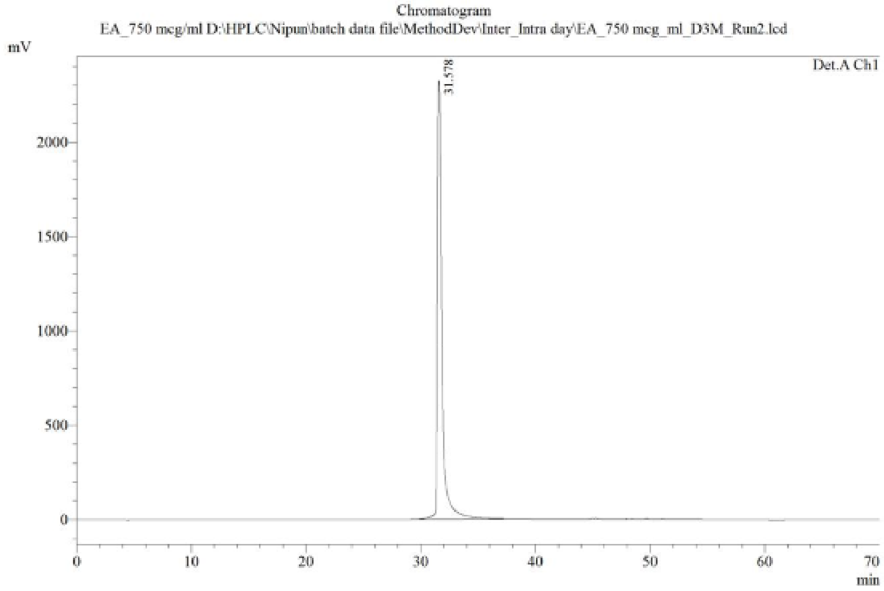


PeakTable D:\HPLC\Nipun\batch data file\MethodDev\Inter_Intra day\EA_750 mcg_ml_D3M_Run2.lcd
Detector A Ch1 260nm

| Peak# | Ret. Time | Area | Height | Area % | Height % |
|---|---|---|---|---|---|
| 1 | 31.578 | 65541036 | 2321143 | 100.000 | 100.000 |
| Total | | 65541036 | 2321143 | 100.000 | 100.000 |

**Figure S30:** Ellagic acid (750 µg/ml) Day 3, Evening

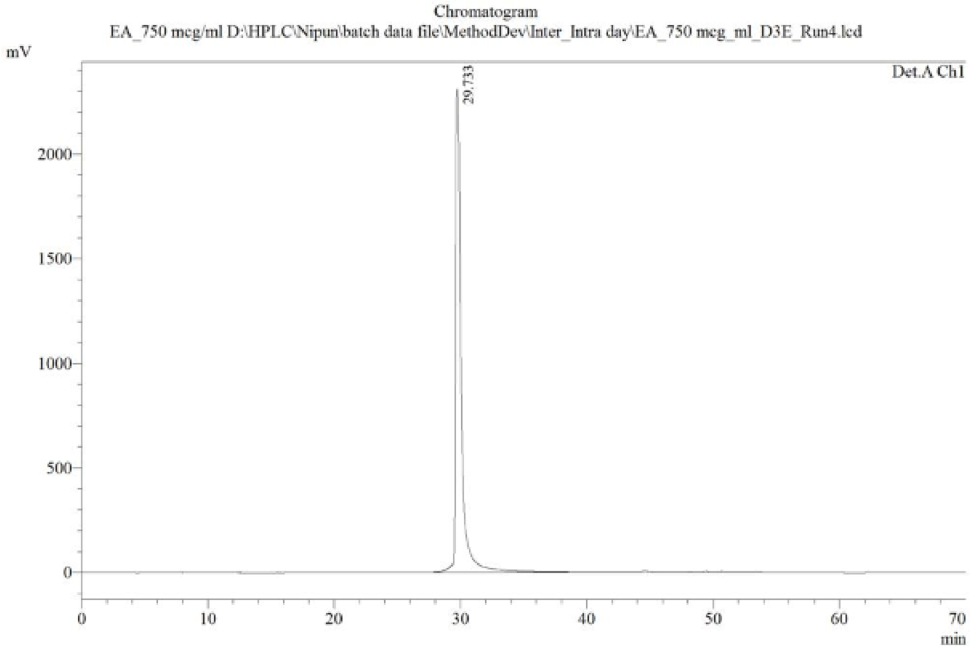


PeakTable D:\HPLC\Nipun\batch data file\MethodDev\Inter_Intra day\EA_750 mcg_ml_D3E_Run4.lcd
Detector A Ch1 260nm

| Peak# | Ret. Time | Area | Height | Area % | Height % |
|---|---|---|---|---|---|
| 1 | 29.733 | 74405484 | 2310060 | 100.000 | 100.000 |
| Total | | 74405484 | 2310060 | 100.000 | 100.000 |

## 2.7 Robustness

**Figure S31:** UV/Vis detector wavelength changed from originally 260 nm to 255 nm Gallic acid (500 µg/ml) (n=3)

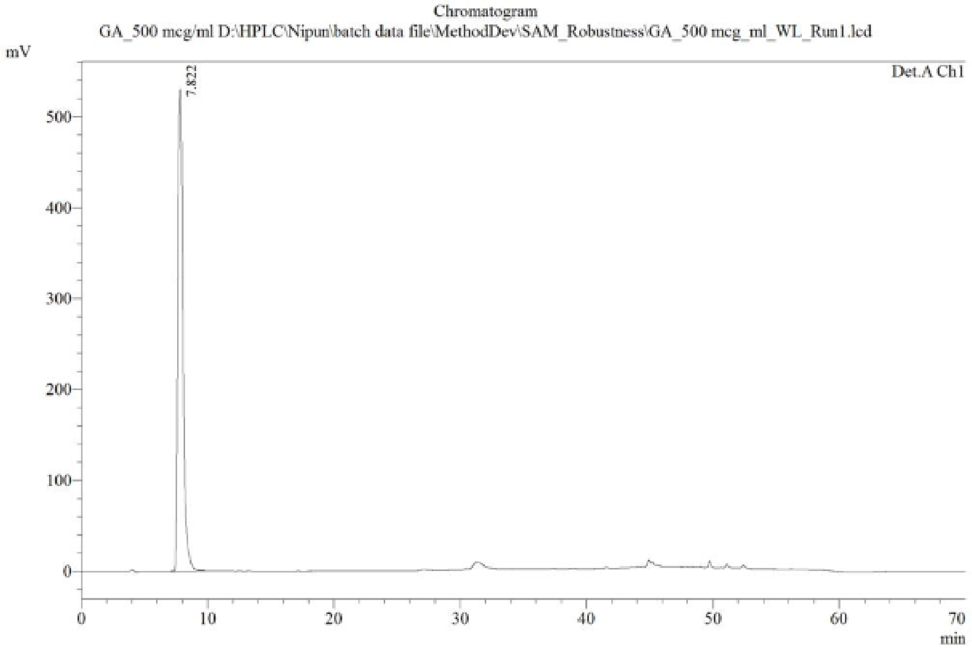


PeakTable D:\HPLC\Nipun\batch data file\MethodDev\SAM_Robustness\GA_500 mcg_ml_WL_Run1.lcd

Detector A Ch1 255nm

| Peak# | Ret. Time | Area | Height | Area % | Height % |
|---|---|---|---|---|---|
| 1 | 7.822 | 15581397 | 530646 | 100.000 | 100.000 |
| Total | | 15581397 | 530646 | 100.000 | 100.000 |

**Figure S32:** UV/Vis detector wavelength at originally 260nm, Gallic acid (500 µg/ml) (n=3)

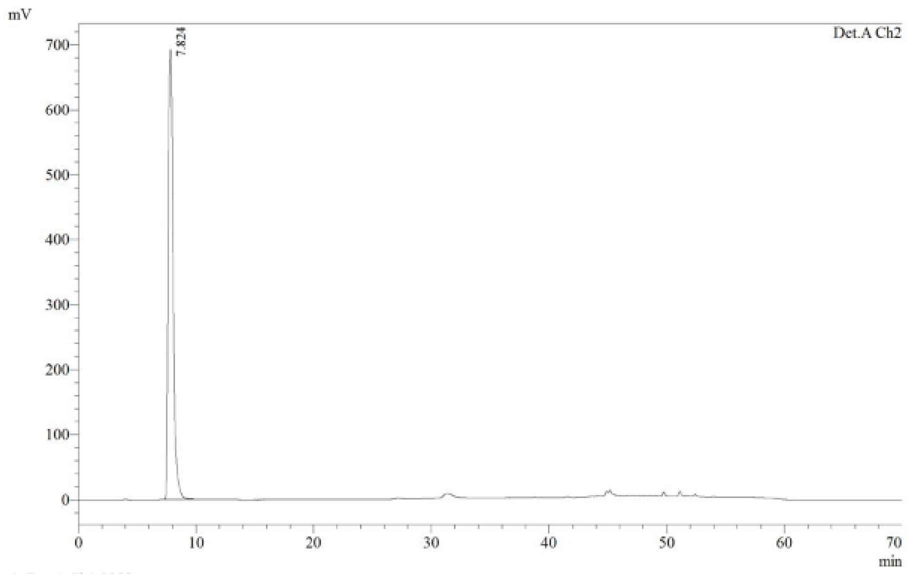


PeakTable

Detector A Ch2 260nm

| Peak# | Ret. Time | Area | Height | Area % | Height % |
|---|---|---|---|---|---|
| 1 | 7.824 | 20314583 | 693205 | 100.000 | 100.000 |
| Total | | 20314583 | 693205 | 100.000 | 100.000 |

**Figure S33:** UV/Vis detector wavelength from originally 260 nm to 255 nm Ellagic acid (750 µg/ml) (n=3)

Chromatogram

EA_750 mcg/ml D:\HPLC\Nipun\batch data file\MethodDev\SAM_Robustness\EA_750 mcg_ml_WL_Run2.lcd

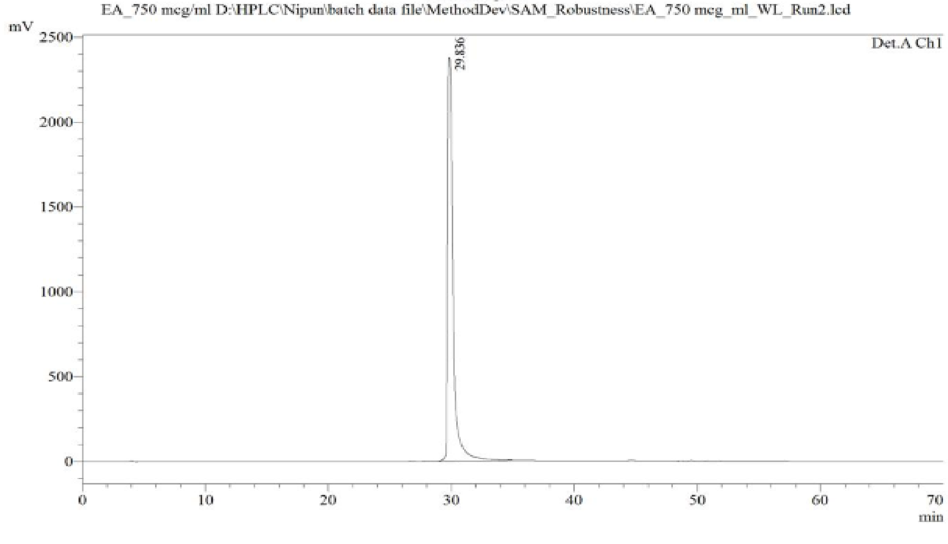


PeakTable D:\HPLC\Nipun\batch data file\MethodDev\SAM_Robustness\EA_750 mcg_ml_WL_Run2.lcd

Detector A Ch1 255nm

| Peak# | Ret. Time | Area | Height | Area % | Height % |
|---|---|---|---|---|---|
| 1 | 29.836 | 73778839 | 2377994 | 100.000 | 100.000 |
| Total | | 73778839 | 2377994 | 100.000 | 100.000 |

**Figure S34:** UV/Vis detector wavelength at originally 260 nm Ellagic acid (750 µg/ml)

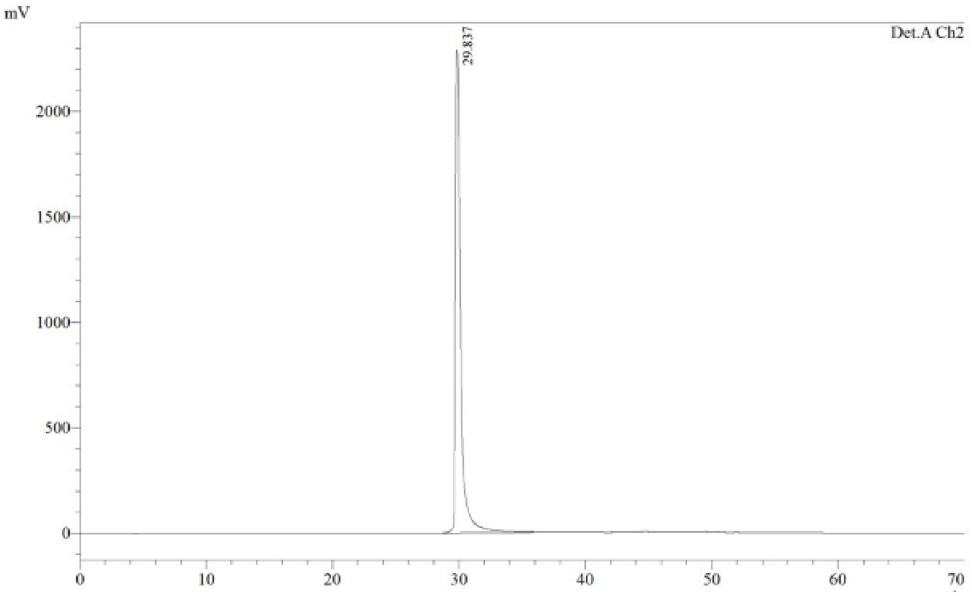


PeakTable

Detector A Ch2 260nm

| Peak# | Ret. Time | Area | Height | Area % | Height % |
|---|---|---|---|---|---|
| 1 | 29.837 | 68179598 | 2289994 | 100.000 | 100.000 |
| Total | | 68179598 | 2289994 | 100.000 | 100.000 |

**Figure S35:** Flow rate from originally 0.80 ml/min to 0.60 ml/min for Gallic acid (500 µg/ml) (n=3)

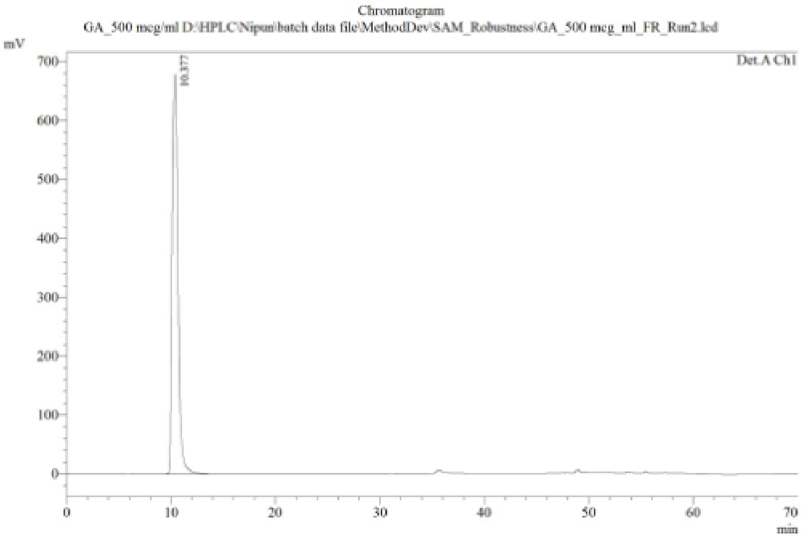


PeakTable D:\HPLC\Nipun\batch data file\MethodDev\SAM_Robustness\GA_500 mcg_ml_FR_Run2.lcd
Detector A Ch1 260nm

| Peak# | Ret. Time | Area | Height | Area % | Height % |
|---|---|---|---|---|---|
| 1 | 10.377 | 26196904 | 677868 | 100.000 | 100.000 |
| Total | | 26196904 | 677868 | 100.000 | 100.000 |

**Figure S36:** Flow rate to 0.80 ml/min to 0.60 ml/min for Ellagic acid (750 µg/ml)

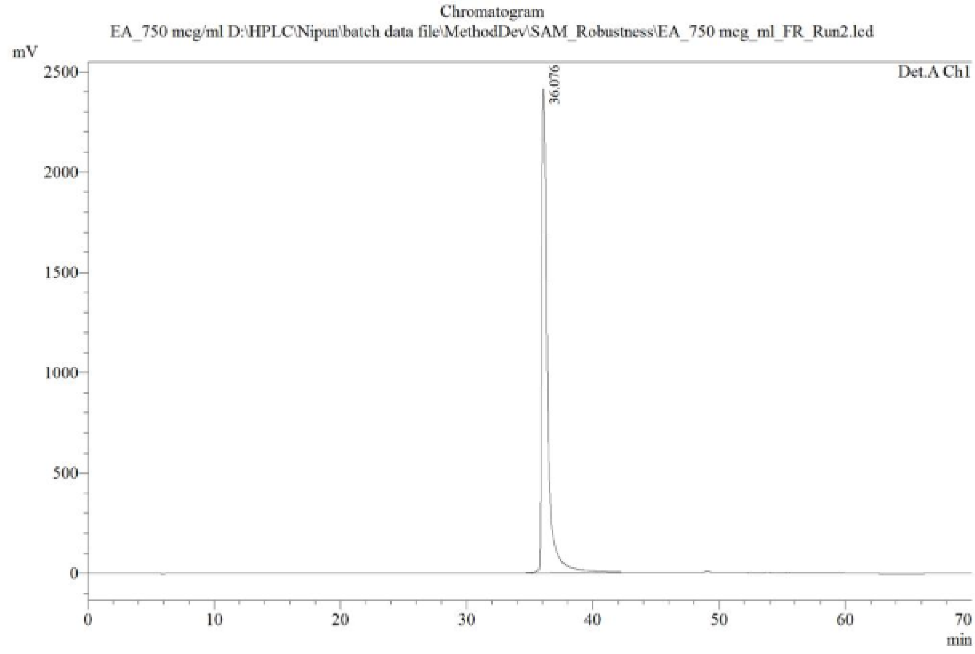


PeakTable D:\HPLC\Nipun\batch data file\MethodDev\SAM_Robustness\EA_750 mcg_ml_FR_Run2.lcd
Detector A Ch1 260nm

| Peak# | Ret. Time | Area | Height | Area % | Height % |
|---|---|---|---|---|---|
| 1 | 36.076 | 78875748 | 2411557 | 100.000 | 100.000 |
| Total | | 78875748 | 2411557 | 100.000 | 100.000 |

## 2.8 Figure S37: HPLC chromatogram of *S. apetala fruit* extract (SAM) 5mg/ml (injection volume= 20µl) (n=3)

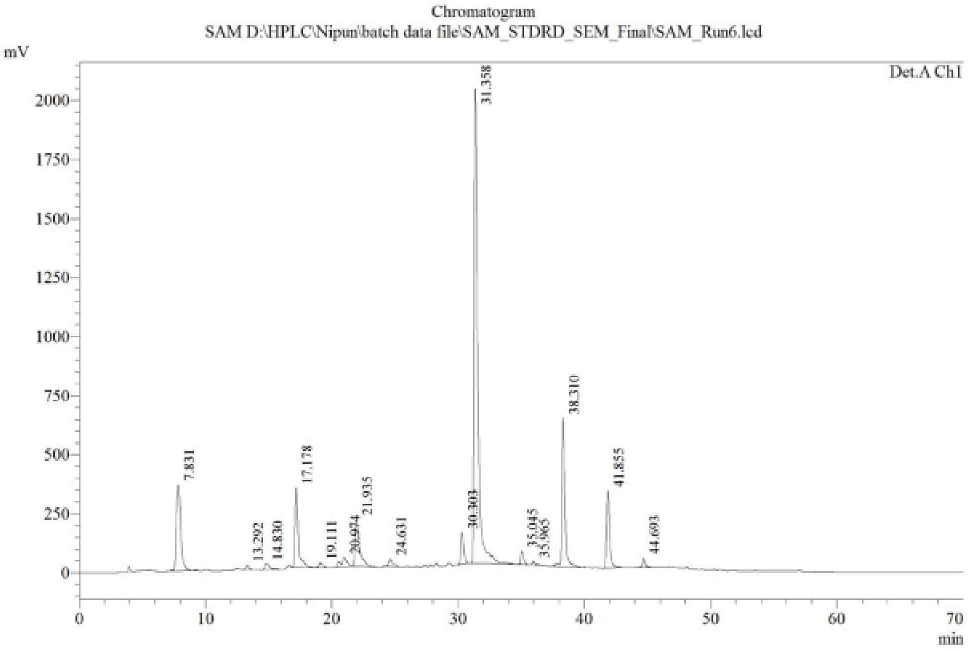


PeakTable D:\HPLC\Nipun\batch data file\SAM_STDRD_SEM_Final\SAM_Run6.lcd

Detector A Ch1 260nm

| Peak# | Ret. Time | Area | Height | Area % | Height % |
|---|---|---|---|---|---|
| 1 | 7.831 | 8943292 | 365948 | 10.416 | 8.609 |
| 2 | 13.292 | 304902 | 18604 | 0.355 | 0.438 |
| 3 | 14.830 | 668791 | 25501 | 0.779 | 0.600 |
| 4 | 17.178 | 6276394 | 338243 | 7.310 | 7.958 |
| 5 | 19.111 | 405779 | 19737 | 0.473 | 0.464 |
| 6 | 20.974 | 1264690 | 39130 | 1.473 | 0.921 |
| 7 | 21.935 | 4828690 | 199281 | 5.624 | 4.688 |
| 8 | 24.631 | 701418 | 31255 | 0.817 | 0.735 |
| 9 | 30.303 | 2106985 | 135322 | 2.454 | 3.184 |
| 10 | 31.358 | 42628133 | 2008889 | 49.648 | 47.262 |
| 11 | 35.045 | 890728 | 55519 | 1.037 | 1.306 |
| 12 | 35.965 | 275636 | 14433 | 0.321 | 0.340 |
| 13 | 38.310 | 10445674 | 630288 | 12.166 | 14.828 |
| 14 | 41.855 | 5554904 | 328914 | 6.470 | 7.738 |
| 15 | 44.693 | 564294 | 39487 | 0.657 | 0.929 |
| Total | | 85860309 | 4250552 | 100.000 | 100.000 |

## 2.9. Figure S39: Mouse treatment protocol

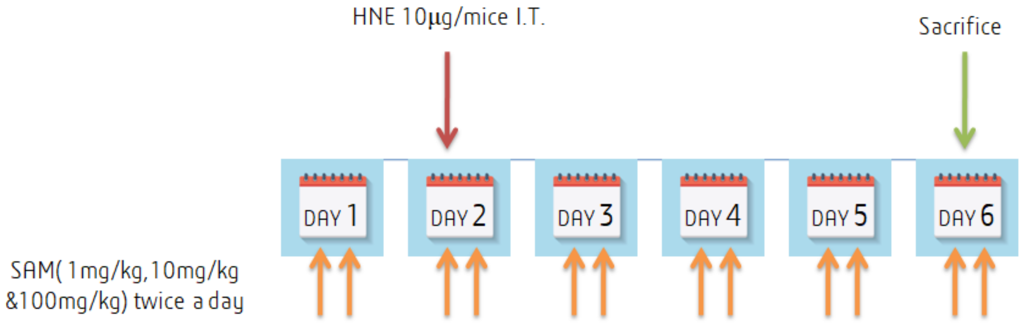

3. **Tables of peak area for HPLC standardisation parameters.**

**3.1 Table S1: Linearity and Sensitivity- Caliberation curve for Gallic acid**

| Gallic acid Concentrationg/ml) | Run 1 Area | Run 2 Area | Run 3 Area |
|---|---|---|---|
| **50** | 1682436 | 1736765 | 1654914 |
| **100** | 3216788 | 3443181 | 3307364 |
| **250** | 8094053 | 7559733 | 7502122 |
| **500** | 17719633 | 16003105 | 16693194 |
| **750** | 25291038 | 22934236 | 21833068 |
| **1000** | 28996343 | 30368882 | 29674647 |

**3.2 Table S2: Linearity and Sensitivity- Caliberation curve for Ellagic acid**

| Ellagic acid Concentration (µg/ml) | Run 1 Peak area | Run 3 Peak area | Run3 Peak area |
|---|---|---|---|
| **50** | 3038561 | 2712005 | 4774086 |
| **100** | 11752761 | 12244928 | 12297027 |
| **250** | 21175410 | 19647227 | 19134710 |
| **500** | 39297875 | 40529195 | 39100909 |
| **750** | 74685725 | 75799922 | 79082024 |
| **1000** | 78675283 | 82109656 | 80568408 |

### 3.3 Table S3: Accuracy: %Recovery study

| | Recovery | |
|---|---|---|
| **(µg/ml)** | **Spiked Peak area** | **Original non spiked peak area** |
| **SAM+ Gallic acid 125 µg Run 1** | 20479754 | 12447294 |
| **SAM+ Gallic acid 125 µg Run 2** | 19841222 | 14001883 |
| **SAM+ Gallic acid 125 µg Run 3** | 19684785 | 19477637 |
| **SAM+ Gallic acid 500 µg Run 1** | 26239882 | 13351543 |
| **SAM+ Gallic acid 500µg Run 2** | 26387439 | 13484065 |
| **SAM+ Gallic acid 500 µg Run 3** | 27551479 | 13746433 |
| **SAM+ Gallic acid 1000 µg Run 1** | 39707804 | 13862985 |
| **SAM+ Gallic acid 1000 µg Run 2** | 42631179 | 14011571 |
| **SAM+ Gallic acid 1000 µg Run 3** | 43901688 | 13518797 |
| **SAM+ Ellagic acid 125 µg Run 1** | 71088370 | 61758563 |
| **SAM+ Ellagic acid 125 µg Run 2** | 76704353 | 56943317 |
| **SAM+ Ellagic acid 125 µg Run 3** | 60860217 | 57441659 |
| **SAM+ Ellagic acid 250 µg Run 1** | 83689571 | 59309107 |
| **SAM+ Ellagic acid 250 µg Run 2** | 88048063 | 58156443 |
| **SAM+ Ellagic acid 250 µg Run 3** | 86827751 | 64232319 |
| **SAM+ Ellagic acid 500 µg Run 1** | 103758260 | 60860217 |
| **SAM+ Ellagic acid 500 µg Run 2** | 107535390 | 69861057 |
| **SAM+ Ellagic acid 500 µg Run 3** | 107057320 | 63235846 |

### 3.4 Table S4: Intra- day and inter-day variation in peak area

| Days | Time | (n=3) | Gallic acid (500µg/ml) | Ellagic acid (750 µg/ml) |
|---|---|---|---|---|
| DAY 1 | Morning | *D1M1* | 15880289 | 64495726 |
| | | *D1M2* | 19436159 | 70724836 |
| | | *D1M3* | 22883764 | 72003695 |
| | Evening | *D1E1* | 26098265 | 78486258 |
| | | *D1E2* | 22574696 | 72263305 |
| | | *D1E3* | 24775682 | 69224310 |
| DAY 2 | Morning | *D2M1* | 22511602 | 74196799 |
| | | *D2M2* | 25408066 | 64461743 |
| | | *D2M3* | 23385571 | 69167815 |
| | Evening | *D2E1* | 24341070 | 56012537 |
| | | *D2E2* | 21380604 | 71136669 |
| | | *D2E3* | 23923683 | 66145241 |
| DAY 3 | Morning | *D3M1* | 16035718 | 68800465 |
| | | *D3M2* | 16513257 | 65541036 |
| | | *D3M3* | 16597530 | 68736873 |
| | Evening | *D3E1* | 22856990 | 74405484 |
| | | *D3E2* | 22087153 | 71339252 |
| | | *D3E3* | 23436351 | 67986969 |

**3.5 Table S5: Robustness: Effect of change in Flow rate (FR) (to 0.6 ml/min) in Wavelength (WL) (to 255 nm) on Peak area of Gallic acid and Ellagic acid (n=3).**

| | ROBUSTNESS | Peak area after modification | Original peak area |
|---|---|---|---|
| **WL** | GA 500 1 | 15581397 | 17719633 |
| | GA 500 2 | 15495803 | 16003105 |
| | GA 500 3 | 15317796 | 16693194 |
| | EA 750 1 | 71143211 | 74685725 |
| | EA 750 2 | 73778839 | 75799922 |
| | EA 750 3 | 75600756 | 79082024 |
| **FR** | GA 500 1 | 24830596 | 17719633 |
| | GA 500 2 | 26196904 | 16003105 |
| | GA 500 3 | 26101216 | 16693194 |
| | EA 750 1 | 80162898 | 74685725 |
| | EA 750 2 | 78875748 | 75799922 |
| | EA 750 3 | 79317452 | 79082024 |

## 5. References

Chang, X., and Kang, W. (2012). Antioxidant and a-glucosidase inhibitory compounds from Pimpinella candolleana wight et arn. *Med. Chem. Res.* 21, 4324–4329. doi: 10.1007/S00044-012-9974-4.

Cuny, E., and Klingler, F. D. (2022). Efficient Isolation and Structure Analysis of (+)-Ranuncoside, a Unique Tricyclic Spiroacetal Glycoside, from Christmas Rose (Helleborus niger L.): *https://doi.org/10.1177/1934578X211069456* 17. doi: 10.1177/1934578X211069456.

Engström, M. T., Pälijärvi, M., and Salminen, J. P. (2015). Rapid fingerprint analysis of plant extracts for ellagitannins, gallic acid, and quinic acid derivatives and quercetin-, kaempferol- and myricetin-based flavonol glycosides by UPLC-QqQ-MS/MS. *J. Agric. Food Chem.* 63, 4068–4079. doi: 10.1021/acs.jafc.5b00595.

Hossain, S. J., Iftekharuzzaman, M., Haque, M. A., Saha, B., Moniruzzaman, M., Rahman, M. M., et al. (2016). Nutrient Compositions, Antioxidant Activity, and Common Phenolics of Sonneratia apetala (Buch.-Ham.) Fruit. *https://doi.org/10.1080/10942912.2015.1055361* 19, 1080–1092. doi: 10.1080/10942912.2015.1055361.

Hossain, S. J., Islam, M. R., Pervin, T., Iftekharuzzaman, M., Hamdi, O. A. A., Mubassara, S., et al.

(2017). Antibacterial, anti-diarrhoeal, analgesic, cytotoxic activities, and gc-ms profiling of sonneratia apetala (Buch.-Ham.) Seed. *Prev. Nutr. Food Sci.* 22, 157–165. doi: 10.3746/pnf.2017.22.3.157.

Jiang, K., Gachumi, G., Poudel, A., Shurmer, B., Bashi, Z., and El-Aneed, A. (2019). The Establishment of Tandem Mass Spectrometric Fingerprints of Phytosterols and Tocopherols and the Development of Targeted Profiling Strategies in Vegetable Oils. *J. Am. Soc. Mass Spectrom.* 30, 1700–1712. doi: 10.1007/s13361-019-02242-2.

Novotny, L., Abdel-Hamid, M. E., Hamza, H., Masterova, I., and Grancai, D. (2003). Development of LC–MS method for determination of ursolic acid: application to the analysis of ursolic acid in Staphylea holocarpa Hemsl. *J. Pharm. Biomed. Anal.* 31, 961–968. doi: 10.1016/S0731-7085(02)00706-9.

Razboršek, M. I., Vončina, D. B., Doleček, V., and Vončina, E. (2008). Determination of oleanolic, betulinic and ursolic acid in Lamiaceae and mass spectral fragmentation of their trimethylsilylated derivatives. *Chromatographia* 67, 433–440. doi: 10.1365/s10337-008-0533-6.

Rozenberg, R., Ruibal-Mendieta, N. L., Petitjean, G., Cani, P., Delacroix, D. L., Delzenne, N. M., et al. (2003). Phytosterol analysis and characterization in spelt (Triticum aestivum ssp. spelta L.) and wheat (T. aestivum L.) lipids by LC/APCI-MS. *J. Cereal Sci.* 38, 189–197. doi: 10.1016/S0733-5210(03)00022-5.

S, Jiang, C.-P, Jiang, P, Cao, et al. (2022). Sonneradon A Extends Lifespan of Caenorhabditis elegans by Modulating Mitochondrial and IIS Signaling Pathways. *Mar. Drugs* 20, 59. doi: 10.3390/md20010059.

Singh, A., Bajpai, V., Kumar, S., Sharma, K. R., and Kumar, B. (2016). Profiling of gallic and ellagic acid derivatives in different plant parts of Terminalia arjuna by HPLC-ESI-QTOF-MS/MS. *Nat. Prod. Commun.* 11, 239–244. doi: 10.1177/1934578X1601100227.

Tahir, N. I., Shaari, K., Abas, F., Parveez, G. K. A., Ishak, Z., and Ramli, U. S. (2012). Characterization of apigenin and luteolin derivatives from oil palm (Elaeis guineensis Jacq.) Leaf using LC-ESI-MS/MS. *J. Agric. Food Chem.* 60, 11201–11210. doi: 10.1021/jf303267e.

Yang, D., Chen, X., Liu, X., Han, N., Liu, Z., Li, S., et al. (2020). Antioxidant and α -Glucosidase Inhibitory Activities Guided Isolation and Identification of Components from Mango Seed Kernel. *Oxid. Med. Cell. Longev.* 2020. doi: 10.1155/2020/8858578.

Yi, X., Jiang, S., Qin, M., Liu, K., Cao, P., Chen, S., et al. (2020). Compounds from the fruits of mangrove Sonneratia apetala: Isolation, molecular docking and antiaging effects using a Caenorhabditis elegans model. *Bioorg. Chem.* 99. doi: 10.1016/j.bioorg.2020.103813.
